# Prostate Cancer Survivorship Essentials for men with prostate cancer on androgen deprivation therapy: protocol for a randomised controlled trial of a tele-based nurse-led survivorship care intervention (PCEssentials Hormone Therapy Study)

**DOI:** 10.1136/bmjopen-2024-084412

**Published:** 2024-03-23

**Authors:** Anna Green, Robert U Newton, David P Smith, Haitham Tuffaha, Daniel A Galvão, Peter Heathcote, Manish I Patel, David Christie, Sam Egger, Sally AM Sara, Nicole Heneka, Suzanne K Chambers, Jeff Dunn

**Affiliations:** 1 Centre for Health Research, University of Southern Queensland, Springfield Central, Queensland, Australia; 2 Exercise Medicine Research Institute, Edith Cowan University, Joondalup, Western Australia, Australia; 3 The Daffodil Centre, a joint venture with Cancer Council NSW, The University of Sydney, Sydney, New South Wales, Australia; 4 The University of Queensland, Saint Lucia, Queensland, Australia; 5 Brisbane Urology Clinic, Brisbane, Queensland, Australia; 6 University of Sydney, Sydney, New South Wales, Australia; 7 Genesiscare, Tugun, Queensland, Australia; 8 Bond University, Robina, Queensland, Australia; 9 Prostate Cancer Foundation of Australia, St Leonards, New South Wales, Australia; 10 Australian Catholic University, Brisbane, Queensland, Australia

**Keywords:** prostatic neoplasms, randomized controlled trial, health education, nursing Care, quality of life

## Abstract

**Introduction:**

Androgen deprivation therapy (ADT) is commonly used to treat men with locally advanced or metastatic prostate cancer. Men receiving ADT experience numerous side effects and frequently report unmet supportive care needs. An essential part of quality cancer care is survivorship care. To date, an optimal effective approach to survivorship care for men with prostate cancer on ADT has not been described. This protocol describes a randomised trial of tele-based nurse-led survivorship that addresses this knowledge gap: (1) determine the effectiveness of a nurse-led survivorship care intervention (PCEssentials), relative to usual care, for improving health-related quality of life (HR-QoL) in men with prostate cancer undergoing ADT and (2) evaluate PCEssentials implementation strategies and outcomes, including cost-effectiveness, compared with usual care.

**Methods and analysis:**

This is an effectiveness-implementation hybrid (type 1) trial with participants randomised to one of two arms: (1) minimally enhanced usual care and (2) nurse-led prostate cancer survivorship essentials (PCEssentials) delivered over four tele-based sessions, with a booster session 5 months after session 1. Eligible participants are Australian men with prostate cancer commencing ADT and expected to be on ADT for a minimum of 12 months. Participants are followed up at 3, 6 and 12 months postrecruitment. Primary outcomes are HR-QoL and self-efficacy. Secondary outcomes are psychological distress, insomnia, fatigue and physical activity. A concurrent process evaluation with participants and study stakeholders will be undertaken to determine effectiveness of delivery of PCEssentials.

**Ethics and dissemination:**

Ethics approval was obtained from the Metro South Health HREC (HREC/2021/QMS/79429). All participants are required to provide written informed consent. Outcomes of this trial will be published in peer-reviewed journals. The findings will be presented at conferences and meetings, local hospital departments, participating organisations/clinical services, and university seminars, and communicated at community and consumer-led forums.

**Trial registration number:**

ACTRN12622000025730.

**STRENGTHS AND LIMITATIONS OF THIS STUDY**

- The effectiveness-implementation design allows for a concurrent process evaluation which will provide immediate implementation data.
- A cost–utility analysis will provide important economic evaluation data.
- Tele-based interventions are highly acceptable to men with prostate cancer and applicable to geographically dispersed and vulnerable populations.
- The pragmatic decision to exclude non-English speaking patients from the trial may influence the generalisability of study findings to patients from linguistically diverse backgrounds.

## Introduction

Prostate cancer (PCa) is the most common cancer diagnosed in Australia.^1^ While men are living longer following diagnosis, longitudinal research has characterised a subgroup of 35%–40% of men who experience long-term decrements in health-related quality of life (HR-QoL).^2^ In particular, men who are on androgen deprivation therapy (ADT) experience consistently poorer physical and mental HR-QoL over the long term.^2–6^

While ADT is effective in treating PCa and increasing survival, it is associated with multiple, often debilitating side effects, which manifest as changes in physical, cognitive, social and sexual functioning.^3 7–9^ Iatrogenic effects may include mood disturbances, increased fat mass, body feminisation, cognitive decline, functional impairment, frailty, fatigue and sexual dysfunction.^3 4 6–10^ ADT also increases the risk of developing new comorbidities, including cardiovascular conditions, diabetes, sarcopenia and osteoporosis.^11^ Compared with men receiving other treatments, those undergoing ADT report poorer HR-QoL and higher levels of psychological distress, including depression, anxiety, relationship changes, cognitive and affective symptoms, and sleep disturbances.^3 4 6–9 12^ The prevalence of psychological distress in PCa survivors is reported to be between 11% and 27%,^13^ and regardless of other treatments, receiving ADT is predictive of higher distress.^12^ Further, men undergoing ADT have an increased risk of suicide compared with those who do not, particularly in older men and in the first 6 months postdiagnosis.^14^ Unmet supportive care needs are highly prevalent in these men, with unmet physical, psychological, sexual, existential and informational^12 15^ needs that persist at 15 years postdiagnosis.^16^ Over one-third (37%) of men with PCa will report at least one long-term unmet supportive care need particularly at the start of treatment when side effects are new or unknown and HR-QoL is first impacted.^16^ This is of particular concern for men receiving ADT who report feeling unprepared to manage substantial treatment side effects that impact on quality of life.^17^ Further, despite routine clinical follow-up, men receiving ADT rarely receive tailored person-centred interventions in a timely manner, adversely impacting HR-QoL with poor management of side effects and self-efficacy.^12 15^ Men treated with ADT are a vulnerable high-need patient group for whom evidence-based survivorship care is crucial.

### Preliminary research on survivorship care for men with PCa

**Figure 1:**
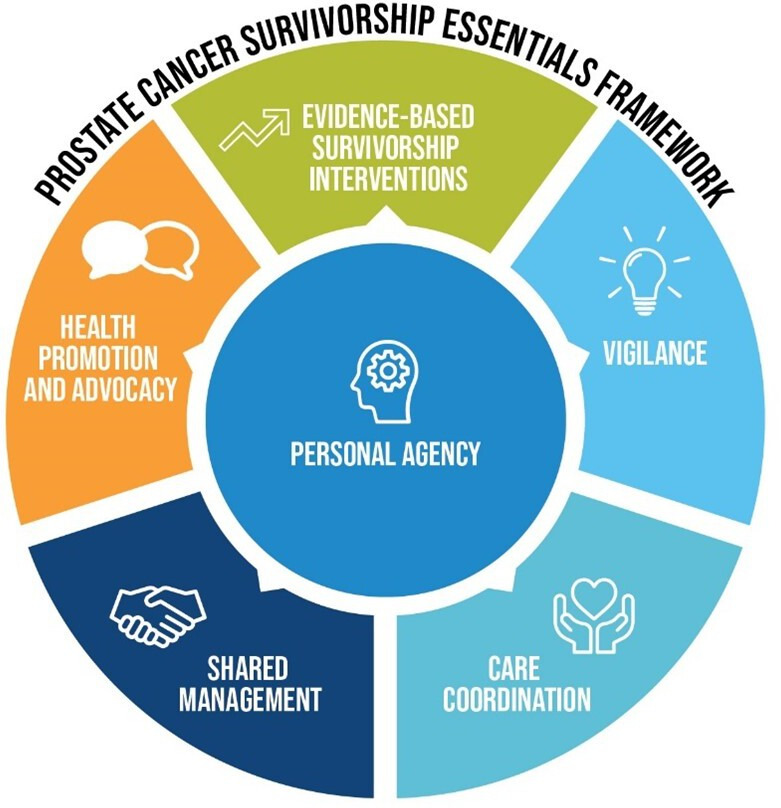
Prostate Cancer Survivorship Essentials Framework.^23^

Previous PCa survivorship guidelines published by the American Cancer Society a decade ago^18^ were limited by an over reliance on expert opinion and lack of a robust evidence base.^19^ Existing survivorship guidelines have also been limited by lack of consumer involvement.^20 21^ Our group has contextualised survivorship care for PCa^20 22 23^ and produced a contemporary survivorship care framework for men with PCa. The resulting survivorship essentials framework (figure 1) proposes holistic survivorship care for men with PCa and was developed by a uniquely inclusive expert clinical and community group.^23^ The framework has been widely endorsed by key PCa and urological groups in Australia and New Zealand. Based on our survivorship framework, we have developed a new model of care, prostate cancer survivorship essentials (PCEssentials), which integrates evidence-based strategies to improve men’s quality of life outcomes after ADT in a men-centred approach, where personal agency intersects with all aspects of care.

We propose an Australian effectiveness-implementation hybrid (type 1) randomised trial^24^ of tele-based nurse-led survivorship care with 236 PCa survivors undergoing ADT. This is the first such study internationally to address this problem. The proposed study will have two arms: (1) minimally enhanced usual care and (2) nurse-led prostate cancer survivorship essentials (PCEssentials) delivered over four tele-based sessions, with a subsequent booster session 5 months after the first session. In accordance with a type 1 hybrid trial, a concurrent process evaluation, guided by the conceptual framework for implementation outcomes,^25^ will be undertaken to determine effectiveness of the PCEssentials intervention delivery, and the potential for implementation of the intervention at scale.

### Aims

Aim 1: Determine the effectiveness of a nurse-led survivorship care intervention (PCEssentials), relative to usual care, for improving HR-QoL in men with PCa undergoing ADT.

Aim 2: Evaluate PCEssentials implementation strategies and outcomes, including cost-effectiveness of PCEssentials, with respect to usual care, as well as acceptability, adoption, appropriateness, feasibility, fidelity, penetration and sustainability.

### Primary hypothesis

We hypothesise that PCEssentials will be more cost-effective than usual care. Furthermore, relative to men receiving usual care at 3, 6 and 12 months after recruitment, men who receive PCEssentials will have: (1) higher HR-QoL, (2) increased self-efficacy, (3) less psychological distress and (4) improved sleep and lower fatigue.

## Methods and analysis

### Study design

A type 1 effectiveness-implementation hybrid randomised trial^24^ of a nurse-led survivorship care intervention (PCEssentials), relative to usual care, for improving HR-QoL in men with PCa undergoing ADT. A concurrent process evaluation will determine the effectiveness of intervention delivery, and the potential for implementation at scale. The study design has been guided by the Consolidated Standards of Reporting Trials (CONSORT) criteria.^26^

There are four key study time points:

- T1—baseline: prior to randomisation.
- T2—3 months postrecruitment.
- T3—6 months postrecruitment.
- T4—12 months postrecruitment.

This study will be undertaken in accordance with the National Statement on Ethical Conduct in Human Research (2007—updated 2018)^27^ and the Australian Code for the Responsible Conduct of Research (2018).^28^ The study commenced in January 2022 on receiving ethics approval, with a planned end date of August 2026.

### Research population

There are two research populations for this study:

1. Patient participants (n=236): Australian men (aged 18 years or over) diagnosed with PCa commencing, or within 3 months of having commenced, ADT.
2. Process evaluation participants (n=148): Study stakeholders (n=30) who are directly involved in study delivery and/or translation into clinical practice, including participating service managers, recruiting clinicians, nurses delivering the intervention, health professionals and patient participants in the intervention group (n=118). While all participants in the intervention group will complete programme acceptability assessments at two study time points (T1 and T3), approximately 20 of these patient participants will be purposively selected/invited to take part in a semistructured interview (T3) to explore their experiences of the intervention. Purposive sampling will ensure a patient subgroup with maximum diversity (eg, based on age, background, location, partnered or unpartnered). We anticipate reaching data saturation for the process evaluation with this number of participants.

### Inclusion criteria

Men recruited to the study will (1) have been diagnosed with PCa and be commencing, or within 3 months of having commenced ADT, and expected (based on clinical information) to be on ADT for a minimum continuous period of 12 months; (2) are able to read and speak English; (3) are able to give written informed consent; (4) have no history of head injury, dementia or psychiatric illness; (5) have no other concurrent cancer and (6) have mobile and/or landline phone access.

### Exclusion criteria

Men with castrate resistant and confirmed metastatic disease are excluded on the basis of having progressive and incurable disease that may rapidly progress and the study doesn’t meet their needs.

### Research project setting/location

There are multiple recruitment settings through clinicians in major treatment centres across Australia and by patient self-referral. Study information for patient self-referral is disseminated through investigator networks.

### Research project procedures

#### Intervention

**Figure 2:**
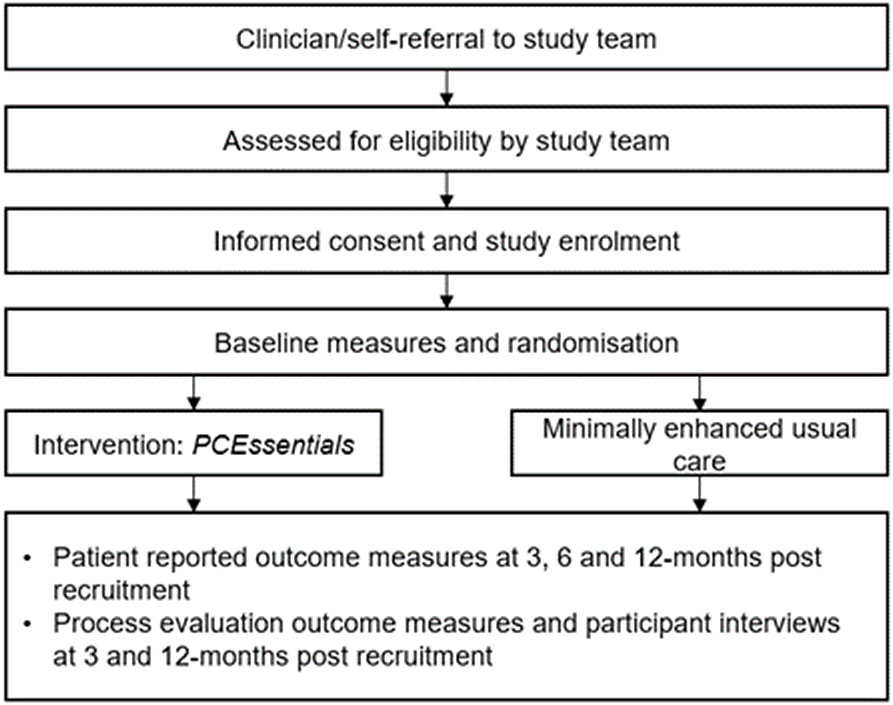
Study diagram.

Following referral (clinician or self) to the study team, research staff screen potential participants for eligibility and conduct an informed consent process (figure 2). Once eligibility is confirmed, and written informed consent received, participants receive the baseline assessments (T1) via mail. On return of T1 assessments, the study team randomises participants into the intervention or minimally enhanced usual care (‘usual care’) group.

Men randomised to the intervention group commence the PCEssentials intervention, a five-session psychoeducation programme delivered by trained PCa specialist nurses via mobile and/or landline telephone. This includes four sessions over 3 months and a booster session at 5 months after the first session. Men in the intervention group are also be offered a home-based exercise programme and encouraged to seek at least one planning session with an accredited exercise physiologist (AEP).

Men in the usual care group receive their standard management, minimally enhanced with a package of evidence-based resources.

Men in both groups will continue to attend their standard PCa related care and complete study assessments at 3, 6 and 12 months postrecruitment.

#### Process evaluation

A mixed-methods approach will examine the elements of the conceptual framework for implementation outcomes^25^ as they relate to the PCEssentials intervention, namely: acceptability, adoption, appropriateness, penetration, feasibility, fidelity and sustainability. To assess programme acceptability and feasibility, clinical stakeholders involved in the delivery or oversight of the programme will be invited by the partner investigator at each site to participate in (1) a short online survey when recruitment commences and ends at the site and (2) a semistructured interview when recruitment ends. Invitations will be sent to eligible clinical stakeholders via email, with written informed consent sought prior to surveys/interviews being undertaken.

### Recruitment

Recruitment is undertaken through clinicians in major treatment centres across Australia. With patient permission, clinicians are asked to directly refer eligible patients to the study team who then proceed with an informed consent process. A two-phase consent process is used for patient participants who are referred by a clinician: (1) written or verbal, where appropriate, permission to provide the patient’s contact details to the study team for follow-up and (2) written informed consent to take part in the study.

Additionally, men may self-refer having identified the study through media promotion and PCa support groups. In this case, potential participants contact the research team directly and provide written informed consent after being screened for eligibility.

Based on our experience with previous interventions in similar cohorts,^29–31^ and active participation of our project partners, we anticipate a recruitment period of 18 months to randomise 236 patients.

### Randomisation

Randomisation to study group condition occurs following receipt of baseline assessments (figure 2). Randomisation occurs in varying block sizes of 4, 6 and 8 (to ensure an unpredictable allocation sequence with equal numbers of men in each treatment group at the completion of each block) with no stratification factors. The randomisation sequence is undertaken by the project manager and concealed from investigators. Project staff tracking assessments (data analysts) will be blinded to condition.

### Research project process

#### Patients

Patient-reported outcomes and experience assessments are completed at each study time point (T1–T4). Following informed consent, participants are sent the T1 assessments for completion. On receipt of completed T1 assessments by the research team, participants are randomised into either (1) minimally enhanced usual care (control) or (2) nurse-led survivorship care: PCEssentials (intervention group).

##### Minimally enhanced usual care

Standard management, minimally enhanced with evidence-based patient education materials about the use of ADT to treat PCa and information about free telephone-based cancer information and support services in the participant’s home state.

##### Nurse-led survivorship care (PCEssentials)

The nurse-led intervention is telephone delivered over five sessions by trained PCa specialist nurses, guided by manualised intervention protocols and supervised by an experienced PCa specialist nurse and a health psychologist with extensive experience in PCa supportive care. The intervention includes five modules covering: psychoeducation with tailored distress management strategies; decision support; treatment education with self-management and skills training for symptom effects, including exercise/physical activity resources and support and communicating with health professionals including a referral pathway to their general practitioner for chronic disease management.

A problem-solving approach that supports personal agency underpins each component,^20^ with the first four sessions to be delivered by telephone over 3 months, and an additional booster session 5 months after the initial session module has been completed. A problem-solving approach^32^ that is responsive to masculine models of coping and life stage was chosen as the underlying mechanism of support to enhance personal agency.

Men with PCa experience improved psychological outcomes when they engage in approach coping that addresses the threats associated with their cancer,^33^ and active problem solving is consistent with male values around strength, self-reliance and action.^34^ Problem-solving therapy (PST) has been found to be effective in reducing depression and disability in older people (>60 years of age) with chronic illness.^32^ Our intervention targets include major challenges identified by men (eg, psychological distress, disease and treatment effects, communicating with health professionals) and applies PST to enhance men’s personal agency in defining and formulating the nature of their specific problems, generating potential solutions, systematically evaluating possible consequences of solutions and selecting an appropriate solution, and monitoring solution outcomes. A self-help survivorship resource that addresses key PCa-related challenges with evidence-based coping strategies is provided and this connects directly to the nurse-led intervention session content.^35^

Distress screening and problem identification occur at each session using the Distress Thermometer and are integrated with distress and symptom management strategies.^36^ The booster session checks participant progress, reinforces self-management skills, and troubleshoots concerns that may have persisted.

A home-based physical exercise programme is offered, where men are encouraged to seek at least one planning session with an AEP within their treatment team, accessed by telephone or internet. The nurse specialist encourages exercise maintenance, including aerobic and resistance training as per the Australian Exercise Medicine for Cancer guidelines with referral to an AEP, if required.^37^

Men have identified that the PCa specialist nurse/clinical nurse is highly acceptable as the provider of survivorship care, an approach described as the most efficient in terms of use and resources and being suitable for most care settings.^38^ Tele-based interventions are also highly acceptable to men with PCa (85% consent rate^22^), are accessible for patients who are very unwell,^39^ have been shown to be an effective delivery method for PST^32^ and in advanced disease show low attrition rates compared with face-to-face delivery.^20^ This delivery method is also applicable to geographically dispersed and vulnerable populations with high potential for population-based translation.

#### Process evaluation

Process assessments are collected via: (1) surveys using the programme acceptability: acceptability of intervention measure (AIM), intervention appropriateness measure (IAM) and feasibility of intervention measure (FIM)^40^ at T1 and T3 (patient participants) and when recruitment commences and ends (other stakeholders), as well as the Working Alliance Inventory-Short Revised (WAI-SR) scale^41^ at T3 (intervention group patient participants only); (2) semistructured interviews with stakeholders at T3 (patient participants) and when recruitment ends (other stakeholders) and (3) intervention fidelity and adherence assessments at multiple study time points, to identify barriers and facilitators to implementation, and determine if high intervention fidelity is achieved.

### Research outcomes and measurement tools

Previously validated and reliable patient-reported outcome assessments are administered by mail to men at four time points: baseline/recruitment (T1), 3 months (T2), 6 months (T3) and 12 months (T4) after recruitment. Primary outcomes are HR-QoL and self-efficacy. Secondary outcomes include global psychological distress, insomnia, fatigue and life satisfaction. Demographic moderators/disease variables (eg, cancer grade, stage, time since diagnosis, time since treatment) and a health service use diary are self-reported. Assessments are self-reports with pen and paper.

#### Primary outcomes

##### Health-related quality of life

The Functional Assessment of Cancer Therapy-Prostate^42^ assesses men’s disease-specific quality of life across five domains: physical, social/family, emotional, functional well-being and PCa-specific concerns.^42^ The Assessment of Quality of Life (AQoL-8D) instrument is used to derive health utility scores and general HR-QoL among patients. This tool has increased measurement sensitivity to psychosocial elements of health compared with other instruments since it comprises five psychosocial dimensions (mental health, happiness, coping, relationships and self-worth) and three physical dimensions (independent living, pain and senses).^43^ The physical function subscale from the Medical Outcomes Study Short-Form-36 questionnaire will be used as an indicator of patient-related physical functioning QoL.^44^ We recently reported improvements in physical function in PCa patients with advanced disease and bone metastases following an exercise intervention using this measure, and in those on ADT with localised disease.^45^

##### Self-efficacy

The 11-item Cancer Survivorship Self-Efficacy Scale^46^ assesses self-efficacy to manage problems arising from cancer and its treatment specifically.

#### Secondary outcomes

##### Psychological distress

The Generalised Anxiety Disorder (GAD-7) scale^47^ and the depression subscale of the Patient Health Questionnaire (PHQ-9)^48^ will measure psychological distress. The seven-item GAD-7 scale screens for, and assesses the severity of, GAD in clinical practice and research. The nine-item PHQ-9 scale screens for, and assesses the severity of, depression and includes a specific item on suicidal ideation.

##### Insomnia

The Insomnia Severity Index is the worldwide standard, seven-item self-report measure to evaluate: (a) severity of sleep-onset, (b) sleep maintenance, (c) early morning awakening problems, (d) satisfaction with current sleep pattern, (e) interference with daily functioning, (f) noticeability of impairment attributed to the sleep problem and (g) level of distress caused by the sleep problem.^49^

##### Fatigue

The Multidimensional Fatigue Symptom Inventory-Short Form^50^ assesses general fatigue, physical fatigue, emotional fatigue, mental fatigue and vigour.

##### Physical activity/exercise

Godin-Shephard Leisure-Time Physical Activity Questionnaire,^51^ modified to include questions on resistance training, reflecting current best practice in exercise intervention trials for men with PCa,^52^ will assess physical activity.

#### Process evaluation

##### Programme acceptability

The AIM, IAM and FIM^33^ are a short self-reported assessment that is collected at T1 and T3 (patient participants) to determine patients’ experiences of the study from recruitment to 6 months postrecruitment. For patient participants, this is, included in the self-reported study assessments mailed to them at T1 and T3. The therapeutic alliance between patients in the intervention group and the nurses delivering the intervention will also be assessed by the 12-item WAI-SR.^41^ This will be included in the self-reported study assessments mailed to patient participants at T3.

All other study stakeholders receive the same assessments as an online survey when recruitment starts and ends to determine their study experience.

##### Interviews

Semistructured interviews exploring the constructs of the conceptual framework for implementation outcomes^25^ will be undertaken to determine effectiveness of the PCEssentials intervention delivery, and the potential for implementation of the intervention at scale. The interview question route informed by the literature is included in online supplemental file 1.

10.1136/bmjopen-2024-084412.supp1 Supplementary data

### Statistical considerations and data analysis

Recent meta-analyses conclude that individually focused psychological interventions should produce improvements in psychological distress of at least a medium effect size (d=0.40) that will be clinically meaningful.^53^ To see an effect of this size or greater in our primary outcome, psychological distress at 12 months, with 80% power and alpha=0.05, we will require 99 participants in each group to complete the intervention. Assuming 15% attrition, we will recruit 236 patients to the study (118 patients per group).

#### Intervention effectiveness

The study is a two-arm randomised controlled trial with repeated assessments across time and with continuous primary outcome variables. Recruitment bias will be assessed by comparing sociodemographic and clinical variables for consenters with non-consenters using t-tests (or Mann-Whitney U tests) for continuous variables and χ^2^ tests for categorical variables. Possible differential attrition will be assessed by comparing baseline characteristics of drop-outs and continuing participants using t-tests (or Mann-Whitney U tests if appropriate) for continuous variables and χ^2^ tests for categorical variables. Intention-to-treat analyses will be conducted. Between-group mean differences in change from baseline outcome scores at 3, 6 and 12 months will be analysed by fitting mixed effects regression models. Intervention (intervention/usual care) will be included as the main effect. Indicators for participants will be included as a random effect to account for the non-independence of repeated observations from the same individual. Sensitivity analysis will assess the effects of attrition. Mixed effects models with maximum likelihood estimation minimise bias that may arise from ignoring missing observations, and use all available data, thereby maximising statistical power to detect effects. The mean and 95% CI will be calculated for satisfaction with the intervention. Missing data will be examined for patterns of missingness and addressed with the appropriate multiple imputation methods, if required. The investigator team includes a dedicated biostatistician who will undertake analyses.

#### Process evaluation

Process evaluation assessments will be analysed using a combination of descriptive statistics (measures of programme acceptability) and deductive directed content analysis (semistructured interviews).^54^ Joint display tables will facilitate the data integration process and facilitate the drawing of inferences from the integrated data.^55^

#### Cost–utility analysis

A cost–utility analysis of the intervention relative to minimally enhanced usual care from both healthcare payer and societal perspectives will be conducted alongside the PCEssentials trial. Costs will be obtained by identifying, measuring and valuing the health resources used. At baseline, participants are given a health service use diary to record direct health resources utilised (eg, general practitioner visits, treatments and hospitalisations), as well as out-of-pocket expenses and indirect costs (eg, productivity loss). The diaries will also be collected during the T2, T3 and T4 assessments. Healthcare resources will be valued using unit prices from standard costing resources such as the Medicare Benefits Schedule and relevant Australian award wages. Quality-adjusted life-years (QALYs) gained will be estimated, which is a measure of a patient’s life expectancy, weighted by his HR-QoL (ie, utility score) measured using the AQoL-8D at baseline, 3, 6 and 12 months. A multivariate generalised linear model will be used to adjust for differences in baseline AQoL-8D scores, demographics and disease classifications. The incremental cost-effectiveness ratio (ICER) will be calculated, which is the difference in mean costs divided by the difference in mean QALYs. Non-parametric bootstrapping will be used to characterise uncertainty around the ICER. If the intervention appears to be cost-effective, we will calculate the expected value of implementation, which is the net monetary benefit of the intervention (ie, monetary benefits—costs) multiplied by the population of PCa patients expected to benefit from the intervention and adjusted by various patients’ adherence and clinicians’ uptake rates. Uptake rates will be obtained from a formal elicitation exercise and will inform a Bass model to forecast diffusion (ie, implementation over time).^56^

### Patient and public involvement

This research project was developed through a collaboration between the University of Southern Queensland and the Prostate Cancer Foundation of Australia as the co-lead organisations. The Prostate Cancer Foundation of Australia is a broad-based community organisation and the peak national body for PCa in Australia. Patient/public involvement in the research has been carried through the conceptualisation and design of the study and PCEssentials intervention, to recruitment and delivery of the intervention through this partnership. Consumer and clinical representatives have contributed to project steering committees and development of the intervention. The Prostate Cancer Foundation of Australia will assist with dissemination of study results through their consumer and clinical stakeholder network ensuring future patient/public engagement.

## Ethics and dissemination

Ethics approval for this study was obtained from the Metro South Health Human Research Ethics Committee (HREC/2021/QMS/79429).

### Safety considerations

Experienced PCa specialist nurses (‘intervention nurses’) are responsible for the delivery of the intervention. Intervention nurses receive: (1) additional training in the study-specific protocol and PCEssentials intervention; (2) an intervention manual detailing session content and activities and (3) weekly supervision and debriefing by study investigators with extensive experience in the delivery of the PCa supportive care. All other study staff will also receive protocol specific and research processes training.

### Data management and monitoring

Written, informed consent is obtained from each patient and clinical stakeholder prior to study enrolment and any study activities being undertaken (online supplemental file 2 and online supplemental file 3). Patient participants are given a unique participant identification code (ID). This ensures that all identifying data can be removed before data analysis commences. This project ID enables the research team to manage the data in a confidential manner. The master list linking identifying participant information and ID number is maintained in a locked cabinet, separate from the participant database at the Prostate Cancer Foundation of Australia. All data collected for each participant are kept in a participant file (identified by ID number only) which contains the case report forms, any corrected and amended data, copies of adverse event reports, file notes, etc. All study files are stored in accordance with Good Clinical Practice guidelines.

10.1136/bmjopen-2024-084412.supp2 Supplementary data

10.1136/bmjopen-2024-084412.supp3 Supplementary data

Form tracking is via participant ID number only. The participant database is stored on a password-protected hard drive maintained by the study investigators. Data will be analysed by ID number only. All information presented in dissemination will be deidentified group data that will not allow the identification of individual participants.

### Treatment fidelity

The intervention is manualised and intervention nurses complete a checklist of components delivered at each session. Throughout the study, sessions are audiotaped and 15% of sessions will be reviewed to assess adherence to protocol. The intervention nurses are supervised by an investigator who is a qualified psychologist with oversight on treatment fidelity monitoring according to National Institutes of Health (NIH) guidelines.^57^

### Ethical considerations

There are two potential risks for participants related to the intervention: (1) minor psychological distress may be experienced by some participants while discussing issues relating to treatment, side effects and psychosocial impact during the intervention; (2) side effects arising from changes in physical activity (such as muscle soreness) if participants choose to take part in the exercise component of the intervention. However, the psychological distress that may be experienced by some participants will be no greater than that experienced when discussing issues related to PCa management with their doctor. Similarly, the side effects that may be experienced by some participants while in the process of the exercise component are likely to be no greater than the risks of day-to-day living as people can undertake changes in their level of physical activity.

Adverse events will be recorded by the research team immediately on their notification. Should any adverse or serious adverse events occur, the research team will report to the governing ethics committee, review relevant risk assessments, aim to mitigate future risk of adverse events and provide the appropriate duty of care to the participant/s concerned.

#### Risk mitigation

Psychological distress will be minimised by identifying those individuals who are experiencing high distress and tailoring the intervention to specifically manage stress in these individuals. The intervention specialist nurses are trained to assess psychological distress and to manage this during the nurse-led intervention. Participants who request additional psychological support beyond the intervention will be referred to additional sources including the Prostate Cancer Foundation of Australia Telenursing Service (direct referral to the telenursing service manager who is not an intervention nurse), Beyond Blue, Lifeline and/or other relevant local services. Medical management of participants will be managed as per their usual care.

### Dissemination

Outcomes of this trial will be published in peer-reviewed journals, and the findings presented at national and international conferences and meetings. Findings will also be communicated at community and consumer-led forums and presented at local hospital departments, participating organisations/clinical services and university seminars. This study is designed so that outputs are translatable into practice to improve the health and well-being of men with PCa receiving ADT. Should it prove effective, our intervention may be used in a range of settings, including broad-reach tele-based support programmes; and through support services across Australia that are conducted by state Cancer Councils and the Prostate Cancer Foundation of Australia, as well as through similar support service infrastructures internationally.

## Conclusion

Men with PCa receiving ADT are a vulnerable high-need patient group. As yet an effective way to deliver holistic survivorship care to improve HR-QoL in this patient population has not yet been identified. The study will provide effectiveness and implementation data to address this knowledge gap and inform the potential for implementation of PCEssentials at scale.

## Supplementary Material

Reviewer comments

Author's manuscript
