# Supplementary material for: Prostate Cancer Survivorship Essentials for men with prostate cancer on androgen deprivation therapy: protocol for a randomised controlled trial of a tele-based nurse-led survivorship care intervention (PCEssentials Hormone Therapy Study)
Source: BMJ Open. 2024 Mar 23;14(3):e084412. doi: 10.1136/bmjopen-2024-084412 (PMC10961537; doi:10.1136/bmjopen-2024-084412)
Supplement: Supplementary data [file bmjopen-2024-084412supp001.pdf]

Supplementary File 1. Question route for interviews

Patient Question Route – Semi-Structured interviews; based on relevant Conceptual Framework for Implementation Outcomes[1]

Italics: Question prompts

Construct/Outcome	Questions
How an individual feels about taking part in an intervention	How did you feel about taking part in *PCEssentials*?<br>• *When you first heard about it*<br>• *While you were taking part*
The extent to which the participant understands the intervention, and how the intervention works	How would you describe what *PCEssentials* was about?
The participant's confidence that they can perform the behaviour(s) required to participate in the intervention	How confident were you that you could do what you needed to take part in *PCEssentials*?<br>• *Access and use resources*<br>• *Contact the intervention nurse*<br>• *Complete the homework*
The perceived amount of effort that is required to participate in the intervention	Do you think *PCEssentials* is easier or harder than coming to the hospital/clinic for care?<br>• *In what way is it easier/harder?*
The extent to which benefits, profits, or values must be given up to engage in an intervention	Did you feel you had to give anything up/miss out on anything to take part in *PCEssentials*? (out of pocket expenses, quality of care)<br>• *Can you give some examples?*<br>• *Do you think the quality of care you received/costs was the same as coming to the hospital/clinic for care?*
The extent to which the intervention has good fit with an individual's value system	Does this type of virtual care meet your needs?<br>• *Why or why not?*<br>• *What could be changed to meet your needs?*<br>• *What was it that really helped meet your needs?*
The extent to which the intervention is perceived as likely to achieve its purpose	Looking back at *PCEssentials* since you started, how effective do you think it is overall?<br>• *In what way is it effective/not effective?*<br>Can you give me an example of something you really liked/disliked about *PCEssentials*?<br>How could *PCEssentials* be improved?<br>Is there anything else you wanted to say about *PCEssentials*?

Clinical Stakeholder Question Route – Semi-Structured interviews; based on relevant Conceptual Framework for Implementation Outcomes[1]

Italics: Question prompts

Construct/Outcome	Questions
How an individual feels about conducting/taking part in an intervention	What were your thoughts about the *PCEssentials* study?<br>• *When you first heard about it*<br>• *While you were recruiting*
The extent to which the participant understands the intervention, and how the intervention works	What is your understanding about how *PCEssentials* works?
The participant's confidence that they can perform the behaviour(s) required to deliver/take part in the intervention	How confident were you that you could do what you needed to deliver/take part in *PCEssentials*?<br>• *Recruitment*<br>• *Conducting the intervention sessions*<br>• *Identifying triggers for care escalation/managing deterioration*
The perceived amount of effort that is required to deliver/take part in the intervention	How burdensome is *PCEssentials* to deliver/take part in compared to usual care?<br>• *In what way is it less/more burdensome?*
The extent to which benefits, profits, or values must be given up to deliver/take part in an intervention	As a clinician do you feel you had to give anything up to deliver/take part in *PCEssentials*?<br>• *Can you give some examples?*<br>Do you think the quality of care delivered in *PCEssentials* differs from usual care?<br>• *Can you give some examples?*<br>From a cost perspective to your service, are there any advantages/disadvantages to the *PCEssentials* model compared to usual care?
The extent to which the intervention has good fit with an individual's value system	Does *PCEssentials* meet your needs as a clinician?<br>• *Why or why not?*<br>• *What could be changed to meet your needs?*<br>• *What was it that helped meet your needs?*
The extent to which the intervention is perceived as likely to achieve its purpose	Looking back at the program since it started, how effective do you think *PCEssentials* is overall?<br>• *In what way is it effective/not effective?*<br>• *Clinician perspective*<br>• *Patient needs*

	Can you give me an example of something you really liked/disliked about *PCEssentials?*<br>How could *PCEssentials* be improved?<br>Is there anything else you wanted to say about *PCEssentials*?

1. Proctor E, Silmere H, Raghavan R, Hovmand P, Aarons G, Bunger A, et al. Outcomes for implementation research: conceptual distinctions, measurement challenges, and research agenda. Administration and policy in mental health. 2011;38(2):65-76.
