# Supplementary material for: Prostate Cancer Survivorship Essentials for men with prostate cancer on androgen deprivation therapy: protocol for a randomised controlled trial of a tele-based nurse-led survivorship care intervention (PCEssentials Hormone Therapy Study)
Source: BMJ Open. 2024 Mar 23;14(3):e084412. doi: 10.1136/bmjopen-2024-084412 (PMC10961537; doi:10.1136/bmjopen-2024-084412)
Supplement: Supplementary data [file bmjopen-2024-084412supp002.pdf]

Prostate Cancer Survivorship Essentials for Men with Prostate Cancer on Androgen Deprivation Therapy (PCEssentials Hormone Therapy Study)

Participant Information Sheet - Patients

**Title**	Prostate Cancer Survivorship Essentials for Men with Prostate Cancer on Androgen Deprivation Therapy: An Effectiveness-Implementation Hybrid (Type 1) Trial of a Tele-Based Nurse-Led Survivorship Care Intervention
**Short Title**	*PCEssentials Hormone Therapy Study*
**Coordinating Principal Investigator**	Professor Jeff Dunn AO

1. Would you like to take part in this study?

You are invited to take part in this research study because you have prostate cancer and are starting, or are planning to start, Androgen Deprivation Therapy (ADT)/hormone therapy. We want to implement and test a new survivorship care intervention delivered by a Prostate Cancer Specialist Nurse via tele-health to identify if it improves the quality of life for men on ADT/hormone therapy and their ability to support their own health and wellbeing. We have called this the *PCEssentials* Intervention. This Participant Information Sheet and Consent Form tells you about the research study to help you decide if you want to take part. It explains the *PCEssentials* Intervention, the study surveys, and the data collection involved. Knowing what is involved, and the potential benefits and risks to you, will help you decide if you want to take part in the research. Please read this information carefully. Ask questions about anything that you don't understand or want to know more about. You might also want to talk to a relative, a friend or your GP before you make up your mind. If you decide to go ahead, we will ask you to sign the 'Participant Consent Form' at the end of this document.

2. What is the purpose of this research?

In this study we test a Prostate Cancer Specialist Nurse-led survivorship intervention for men on ADT/hormone therapy called *PCEssentials*. The study has been designed to fill the survivorship care gap for men on ADT/hormone therapy. An essential part of any quality cancer care is survivorship care. Survivorship care starts at the time of cancer diagnosis and continues throughout the lifespan. The goal of survivorship care is to provide personalised care and support self-management with a strong focus on the patients' needs and experiences. This includes supporting a person through the cancer diagnosis, making decisions about treatment, managing side effects, and maintaining health and wellbeing during and after treatment. Unfortunately survivorship care is often not delivered well, or easily accessible, especially for people living in regional and remote areas, and there is currently no survivorship care model for men on ADT/hormone therapy.

The *PCEssentials* Intervention is a survivorship care model for men on ADT/hormone therapy that will provide one-to-one psychological support, treatment education, tailored strategies to help manage distress, decision making and self-management. It will also include a home-based exercise activity program. The research will identify if this way of providing survivorship care to men on ADT/hormone therapy improves their quality of life and ability to support their own health and wellbeing.

This research has been initiated by Professor Jeff Dunn AO –Chief of Mission and Head of Research, Prostate Cancer Foundation of Australia and Professor and Chair of Cancer Survivorship at the University of Southern Queensland, and

Green A, et al. BMJ Open 2024; 14:e084412. doi: 10.1136/bmjopen-2024-084412

has been funded by the National Health and Medical Research Council (NHMRC) which is administered by the federal Department of Health.

3. Your participation is voluntary

Your participation in this study is completely voluntary. If you do not want to take part in this study, you do not have to. You should feel under no obligation to participate in this study. If you decide to take part and later change your mind, you are free to withdraw at any stage. Choosing not to take part in this study, or if you choose to take part and then later withdraw, will not affect your current and future medical care in any way. Your choice will not affect your relationship with those treating you, or with any institutions involved in this research.

4. Your withdrawal from the study

If you decide to withdraw from the study, you will be offered the usual care delivered by your specialist team. You can choose to withdraw from:

- the whole study: where we stop collecting any data about you **OR**
- part of the study involving your active participation (i.e., completing questionnaires, participating in the interview)

After you have started your participation in this research study, you are under no obligation to continue, and can change your mind at any time about participating in the research. People withdraw from studies for various reasons, and you do not need to provide a reason. You can withdraw at any time by contacting the research team or completing and signing the 'Participant Withdrawal of Consent Form'. This form is located at the end of this document. If you withdraw from the study, you will be able to choose whether the study will destroy or be-able to retain the information collected about you. You should only choose **one** of these options. Where both boxes are ticked in error or neither box is ticked, the study will destroy all information it has collected about you.

5. What does participation in this research involve?

Sometimes we need to compare different models of care to find out which is the best. To do this, we put people into groups and give each group a different model of care. We can then compare the groups to see if one model of care is better than the other. To make sure the groups are the same, each participant is put into a group by chance (random): like flipping a coin. This is called a randomised controlled study and it is designed to make sure we can interpret the results in a fair and appropriate way and to avoid doctors or participants jumping to conclusions about what is best.

In the *PCEssentials Hormone Therapy* Study, we will randomly allocate about 236 men with prostate cancer who are starting, or are planning to start, ADT/hormone therapy from treatment centres across Australia to receive the *PCEssentials* Intervention or usual care at the discretion of a patients specialist team (the Usual Care group). You will have a 50% chance of being in either the *PCEssentials* Intervention group or the Usual Care group. We will follow everyone up to 12 months after recruitment to the study.

If you decide you want to take part in the research study, you will be asked to sign the Consent Form. By signing it you are telling us that you:

- ✓ Understand what participation in *PCEssentials Hormone Therapy* Study will involve
- ✓ Consent to take part in the study as described
- ✓ Give permission for the *PCEssentials Hormone Therapy* Study team to access your personal information during the study
- ✓ Consent to the use of your personal and health information as described.

Green A, *et al. BMJ Open* 2024; 14:e084412. doi: 10.1136/bmjopen-2024-084412

- ✓ Understand that you will be randomly allocated to one of the two models of survivorship care.

There are no costs associated with participating in this research study, nor will you be paid.

5.1 What do I need to do?

If you agree to participate, a member of the *PCEssentials Hormone Therapy* Study team will provide you with detailed information about the study. You will first be asked to complete the study surveys to tell us about yourself. You will then be randomly allocated to one of two study groups: *PCEssentials* Intervention (the new model of care) or specialist-led model of survivorship care (the current practice). It is important for you to understand that we do not know which model of care will be better for you or other men with prostate cancer on ADT/hormone therapy, which is the reason we are conducting this research, thus it is important to follow the model of care that you are randomly assigned. This will help us answer important research questions to improve cancer survivorship care for men with prostate cancer on ADT/hormone therapy across Australia.

If you are allocated to the *PCEssentials* Intervention, you will receive a five-session psychoeducation program delivered by a Prostate Cancer Specialist Nurse via tele-health which includes four sessions over three months and a booster session at six months after the first session. The nurse-led intervention will include five modules covering:

- ✓ Psycho-education with tailored distress management strategies;
- ✓ Decision support;
- ✓ Treatment education with self-management and skills training for symptom effects, including exercise/physical activity resources and support;
- ✓ Communicating with health professionals including a referral pathway to your general practitioner for a Chronic Disease Management plan (CDM)

You will also be provided with a home-based exercise activity program and be encouraged to seek at least one planning session with an Accredited Exercise Physiologist (AEP) within your treatment team, which may be by tele-health as appropriate.

If you are allocated to Usual Care, you will continue to be cared for by your specialist team as usual minimally enhanced by a package of resources containing patient education materials about the use of ADT/hormone therapy to treat prostate cancer; and advice about referral to support services.

5.2 Complete study surveys (20-30 minutes each)

Whether you receive the *PCEssentials* Intervention or Usual Care, a member of the *PCEssentials Hormone Therapy* Study team will contact you at four points during the active study period (12 months from when you begin the study) to ask you to complete the study surveys so that we can find out more about you, your health, and your healthcare experience before you start the study, and at 3 months, 6 months, and 12 months after you start the study. The *PCEssentials Hormone Therapy* Study team will send paper surveys delivered to you through Australia Post. The surveys will take about 20-30 minutes each to complete (depending on how you choose to complete them) and a little longer at the first time point. The *PCEssentials Hormone Therapy* Study team may send reminders to you via post, phone call, text, or email, as required.

5.3 Additional opportunity

There is an additional opportunity for involvement in this research study, <u>which is optional:</u>

- **Interview:** A member of the *PCEssentials Hormone Therapy* Study team may contact you during the research study to invite you to participate in a one-off, individual interview to find out about your experience of participating in this research study. This interview is completely voluntary and can be stopped at any time. It

Green A, *et al. BMJ Open* 2024; 14:e084412. doi: 10.1136/bmjopen-2024-084412

will be audio-recorded to allow the research team to reflect and analyse the interview data later. The interview should take no longer than about 30 minutes.

6. What are the alternatives to participation?

You do not have to take part in this research to receive care. Other options are available. Whether or not you choose to participate in this research, you will still be offered the usual care delivered by your specialist team. The *PCEssentials Hormone Therapy* Study team will discuss these options with you before you decide whether to take part in this research study. You can also discuss the options with your local doctor.

7. What are the possible benefits of taking part?

We cannot guarantee that you will receive any benefits from this research; however, possible benefits may include an improvement in your health and experience of care from the survivorship care approach (i.e., the intervention we are trialling). Your taking part in this project will provide us with important information about survivorship care for men on ADT/hormone therapy, which will be helpful to patients in the future.

8. What are the possible risks and disadvantages of taking part?

There are minimal risks associated with your participation in this study. There is a very small possibility that you might experience some distress because the study surveys cover personal questions relating to possible symptoms, your cancer, and your experience of care. If you receive the *PCEssentials* Intervention, you may experience some distress while discussing issues relating to treatment, side-effects and psychosocial impact during the intervention. You may also experience side-effects arising from changes in physical activity (such as muscle soreness) if you choose to take part in the exercise component of the intervention. If you do become upset because of the research study, you should contact the *PCEssentials Hormone Therapy* Study team, or talk to your Prostate Cancer Specialist Nurse or doctors who will be able to arrange for counselling or other appropriate support.

9. How will you use any tissues or samples you take from me?

We will not collect any tissues or samples from you in this study.

9.1 Will you be doing any genetic tests?

There are no genetic tests in this study.

10. What if new information arises during this research study?

Sometimes during a research study, new information becomes available about the treatment that is being studied. If this happens, the *PCEssentials Hormone Therapy* Study team will tell you about it and discuss with you whether you want to continue in the research study. If you decide to withdraw, the *PCEssentials Hormone Therapy* Study team will arrange for your regular health care to continue. If you decide to continue in the research study, you will be asked to sign an updated consent form.

11. What happens when the research study ends?

We will not contact you again after your 12-month active participation period (which is the 12 months after you begin the study). If you would like to receive a copy of the results at the end of the *PCEssentials Hormone Therapy* Study, please indicate this on the Consent Form or contact the *PCEssentials Hormone Therapy* Study team and we will send this with our compliments.

12. Could the researchers stop the study early?

Yes, if it does, the *PCEssentials Hormone Therapy* Study team will let you know and explain the reason behind the decision. If the study stops early, you will continue to be cared for by your specialist team as usual.

Green A, *et al. BMJ Open* 2024; 14:e084412. doi: 10.1136/bmjopen-2024-084412

13. Privacy, Confidentiality and Disclosure of Information

We will keep all personal information confidential and securely stored. The electronic data we collect about you will be stored on a secure server hosted by the University of Southern Queensland. Hard copies of research data will be stored securely at the Prostate Cancer Foundation of Australia (PCFA) St Leonards office. Any information obtained in connection with this project that could identify you will remain confidential. Only authorised study staff will have access to these materials. It will only be disclosed with your permission, except as required by law. In any publication, information will be provided in such a way that you cannot be identified. Data will be stored for 25 years in accordance with the National Statement (2007) and institutional policy.

Australian privacy law gives you the right to request access to your information that the researchers have collected and stored. The law also gives you the right to request corrections to any information about you that you disagree with. Please contact the study team (see page 5 of this document) if you would like to access your information.

So that we can contact you to take part in an interview, we will ask you to provide an email address or a phone number. This will not be linked to any information we have about you in connection to the project.

14. Who is organising and funding the research?

This research study is being conducted by Professor Jeff Dunn AO, University of Southern Queensland, in partnership with the Prostate Cancer Foundation Australia (PCFA), Cancer Council Queensland (CCQ), Australian Prostate Centre (APC), Ipswich West Moreton Hospital Health Service (WMHHS) GenesisCare, Icon Group, Healthy Male, and the Union for International Cancer Control (UICC). The University of Southern Queensland will receive a payment from the NHMRC administered by the federal Department of Health for undertaking this research. No member of the *PCEssentials Hormone Therapy* Study team will receive any financial benefit from your involvement in this research study (other than their ordinary wages).

15. Who has reviewed the research study?

All research in Australia involving humans is reviewed by an independent group of people called a Human Research Ethics Committee (HREC). The ethical aspects of this research study have been approved by the Metro South HREC. This research study will be carried out according to the National Statement on Ethical Conduct in Human Research (2007). This statement has been developed to protect the interests of people who agree to participate in human research studies.

16. What if I have a question or need to make a complaint?

We have included several contacts for you below. The person you may need to contact will depend on the nature of your query.

For questions about the *PCEssentials Hormone Therapy* Study, you can contact the *PCEssentials Hormone Therapy* Study team:

- Coordinating Principal Investigator: Professor Jeff Dunn AO, *[phone TBA]*
- Central Management Team: Dr Anna Green, *[phone, email TBA]*

To talk to someone at your treatment centre:

- Principal Investigator *[Study Site]*: *[Name & contact number]*

Green A, *et al. BMJ Open* 2024; 14:e084412. doi: 10.1136/bmjopen-2024-084412

If you wish to discuss the study with someone who is not directly involved, particularly in relation to matters concerning complaints about the conduct of the study, or your rights as a participant, you can contact:

Lead HREC Office	Metro South Health and Hospital Services (MSHHS)
Contact Person	HREC Coordinator
Telephone	+61 7 3443 8049
Email	MSH-Ethics@health.qld.gov.au
HREC Reference Number	*[HREC approval number]*

Site HREC Office	*[Institute Ethics Office]*
Contact Person	*[Contact Person]*
Telephone	*[Telephone]*
Email	*[Email]*
HREC Reference Number	*[HREC approval number]*

17. The Participant Consent Form

Sign the consent form only after you have made up your mind to take part in this study. You must be provided with a signed and dated copy of the participant information and consent form for your personal record.

Green A, *et al. BMJ Open* 2024; 14:e084412. doi: 10.1136/bmjopen-2024-084412

Prostate Cancer Survivorship Essentials for Men with Prostate Cancer on Androgen Deprivation Therapy (PCEssentials Hormone Therapy Study)

Participant Consent Form

**Title**	Prostate Cancer Survivorship Essentials for Men with Prostate Cancer on Androgen Deprivation Therapy: An Effectiveness-Implementation Hybrid (Type 1) Trial of a Tele-Based Nurse-Led Survivorship Care Intervention
**Short Title**	*PCEssentials Hormone Therapy Study*
**Coordinating Principal Investigator**	Professor Jeff Dunn AO

Declaration by Participant

I have read, or have had read to me, and I understand the Participant Information Sheet and Consent Form.

I understand the purposes, procedures and risks of the research described in the Participant Information Sheet.

I have had an opportunity to ask questions and I am satisfied with the answers I have received.

I freely agree to participate in this research study as described and understand that I am free to withdraw at any time during the study without affecting my future health care.

I consent to my treating doctor/s being notified of my participation in this study and any clinically relevant information noted by the *PCEssentials Hormone Therapy* Study team in the conduct of the study.

I understand that I will be given a signed copy of this document to keep for my own records. We may ask you to participate in a future related study, or to obtain additional information or clarification related to your participation in this study. **Please indicate below** whether you are willing to be contacted about any future research studies.

I agree to the research team using, reproducing, and disclosing audio-recordings as explained in the Participant Information Sheet/Consent Form for Patients.

I agree to be audio-recorded and understand that, subject to any constraints requested below, recordings may be used in presentations and publications for educational and research purposes.

30-minute Interview (Optional)

☐ Yes, I agree to be contacted and invited to participate in the interview

☐ No, I do not want to be contacted to be invited to participate in the interview

Future Studies

☐ Yes, I agree to be contacted about future research studies

☐ No, I do not want to be contacted about future research studies

Green A, *et al. BMJ Open* 2024; 14:e084412. doi: 10.1136/bmjopen-2024-084412

Prostate Cancer Foundation of Australia | PROSTATE CANCER SURVIVORSHIP ESSENTIALS | University of Southern Queensland

Study Results

☐ Yes, I would like to receive a copy of the study results and acknowledge that these will be provided in aggregate as a summary (individual results will not be available)

☐ No, I do not want a copy of the study results

Participant

Signature ________________________________ Date _________________________

Name (please print) ________________________________

Declaration by senior researcher

I have given a verbal explanation of the study, its procedures and risks and I believe that the participant has understood that explanation.

Signature ________________________________ Date _________________________

Name (please print) ________________________________

Green A, *et al. BMJ Open* 2024; 14:e084412. doi: 10.1136/bmjopen-2024-084412
