# Supplementary material for: Prostate Cancer Survivorship Essentials for men with prostate cancer on androgen deprivation therapy: protocol for a randomised controlled trial of a tele-based nurse-led survivorship care intervention (PCEssentials Hormone Therapy Study)
Source: BMJ Open. 2024 Mar 23;14(3):e084412. doi: 10.1136/bmjopen-2024-084412 (PMC10961537; doi:10.1136/bmjopen-2024-084412)
Supplement: Supplementary data [file bmjopen-2024-084412supp003.pdf]

Prostate Cancer Survivorship Essentials for Men with Prostate Cancer on Androgen Deprivation Therapy (PCEssentials Hormone Therapy Study)

Participant Information Sheet – Clinical Stakeholders

**Title**	Prostate Cancer Survivorship Essentials for Men with Prostate Cancer on Androgen Deprivation Therapy (ADT): An Effectiveness-Implementation Hybrid (Type 1) Trial of a Tele-Based Nurse-Led Survivorship Care Intervention
**Short Title**	*PCEssentials Hormone Therapy Study*
**Coordinating Principal Investigator**	Professor Jeff Dunn AO

1. Would you like to take part in this study?

You have been invited to take part in this study because you are part of the clinical and/or administrative teams involved in recruitment or delivery of the *PCEssentials* Intervention. We want to implement, test and evaluate a new survivorship care intervention delivered by a Prostate Cancer Specialist Nurse via tele-health (*PCEssentials)* to identify if it improves the quality of life for men on androgen deprivation therapy (ADT) and their ability to support their own health and wellbeing.

This Participant information and consent handout contains information about the *PCEssentials Hormone Therapy* study. Its purpose is to explain to you as openly and clearly as possible all the procedures involved in this project before you decide whether or not to take part.

Please read this Participant information and consent handout carefully. Please free to ask questions about any information in the handout or about the project. You will be asked to sign the Consent Form if you agree to participate. By signing the Consent Form, you indicate that you understand the information and that you give your consent to participate in the research project.

2. What is the purpose of this research?

In this study we test and evaluate a Prostate Cancer Specialist Nurse-led survivorship intervention for men on ADT (*PCEssentials*). The study has been designed to fill the survivorship care gap for men on ADT. An essential part of any quality cancer care is survivorship care. Survivorship care starts at the time of cancer diagnosis and continues throughout the lifespan. The goal of survivorship care is to provide personalised care and support self-management with a strong focus on the patients' needs and experiences. This includes supporting a person through the cancer diagnosis, making decisions about treatment, managing side effects, and maintaining health and wellbeing during and after treatment. Unfortunately survivorship care is often not delivered well, or easily accessible, especially for people living in regional and remote areas, and there is currently no survivorship care model for men on ADT.

The *PCEssentials* Intervention is a survivorship care model for men on ADT that will provide one-to-one psychological support, treatment education, tailored strategies to help manage distress, decision making and self-management. It will also include a home-based exercise activity program. The research will identify if this way of providing survivorship care to men on ADT improves their quality of life and ability to support their own health and wellbeing.

This research has been initiated by Professor Jeff Dunn AO –Chief of Mission and Head of Research, Prostate Cancer Foundation of Australia and Professor and Chair of Cancer Survivorship at the University of Southern Queensland, and has been funded by the National Health and Medical Research Council (NHMRC) which is administered by the federal Department of Health.

Green A, *et al. BMJ Open* 2024; 14:e084412. doi: 10.1136/bmjopen-2024-084412

3. Your participation is voluntary

Your participation in this study is completely voluntary. If you do not want to take part in this study, you do not have to, and are not obliged to. If you decide to take part and later change your mind, you are free to withdraw at any stage. Choosing not to take part in this study, or if you choose to take part and then later withdraw, will not affect your current employment or relationship with any institutions involved in this research.

4. Your withdrawal from the study

After you have started your participation in this research study, you are under no obligation to continue, and can change your mind at any time about participating in the research. People withdraw from studies for various reasons, and you do not need to provide a reason. You can withdraw at any time by notifying the research team or completing and signing the 'Participant Withdrawal of Consent Form'. This form is located at the end of this document. If you withdraw from the study, you will be able to choose whether the study will destroy or be-able to retain the information collected about you. You should only choose **one** of these options. Where both boxes are ticked in error or neither box is ticked, the study will destroy all information it has collected about you.

5. What does participation in this research involve?

If you agree to participate in this study, you will be asked to complete two short online surveys (approximately five minutes to complete) and take part in a semi-structured interview. Interviews will be conducted by phone or videoconference by a member of the research team to explore your perceptions of the acceptability and feasibility of *PCEssentials*. It will be audio-recorded to allow the research team to reflect and analyse the interview data later. This interview will take 15-30 minutes to complete.

There are no costs associated with participating in this research study, nor will you be paid.

6. What are the possible benefits of taking part?

We cannot guarantee that you will receive any direct benefits from this research; however, your participation in this project will provide us with important information about survivorship care for men on ADT, which will be helpful to clinicians, health services and patients in the future.

7. What are the possible risks and disadvantages of taking part?

There are minimal risks associated with your participation in this study. The primary risk is the inconvenience related to the time it takes to complete the online surveys and take part in the interview.

8. Privacy, Confidentiality and Disclosure of Information

We will keep all personal information confidential and securely stored. The electronic data we collect about you will be stored on a secure server hosted by the University of Southern Queensland. Hard copies of research data will be stored securely at the Prostate Cancer Foundation of Australia (PCFA) St Leonards office. Any information obtained in connection with this project that could identify you will remain confidential. Only authorised study staff will have access to these materials. It will only be disclosed with your permission, except as required by law. In any publication, information will be provided in such a way that you cannot be identified. Data will be stored for 25 years in accordance with the National Statement (2007) and institutional policy.

So that we can contact you to take part in an interview, we will ask you to provide an email address or a phone number. This will not be linked to any information we have about you in connection to the project.

Green A, *et al. BMJ Open* 2024; 14:e084412. doi: 10.1136/bmjopen-2024-084412

Australian privacy law gives you the right to request access to your information that the researchers have collected and stored. The law also gives you the right to request corrections to any information about you that you disagree with. Please contact the study team (see page 3 of this document) if you would like to access your information.

9. Who is organising and funding the research?

This research study is being conducted by Professor Jeff Dunn AO, University of Southern Queensland, in partnership with the Prostate Cancer Foundation Australia (PCFA), Cancer Council Queensland (CCQ), Australian Prostate Centre (APC), Ipswich West Moreton Hospital Health Service (WMHHS) GenesisCare, Icon Group, Healthy Male, and the Union for International Cancer Control (UICC). The University of Southern Queensland will receive a payment from the NHMRC administered by the federal Department of Health for undertaking this research. No member of the *PCEssentials Hormone Therapy* Study team will receive any financial benefit from your involvement in this research study (other than their ordinary wages).

10. Who has reviewed the research study?

All research in Australia involving humans is reviewed by an independent group of people called a Human Research Ethics Committee (HREC). The ethical aspects of this research study have been approved by the Metro South HREC. This research study will be carried out according to the National Statement on Ethical Conduct in Human Research (2007). This statement has been developed to protect the interests of people who agree to participate in human research studies.

11. What if I have a question or need to make a complaint?

We have included several contacts for you below. The person you may need to contact will depend on the nature of your query.

For questions about the *PCEssentials Hormone Therapy* Study, you can contact the *PCEssentials Hormone Therapy* Study team:

- Coordinating Principal Investigator: Professor Jeff Dunn AO, 02 9428 7060
- Central Management Team: Dr Anna Green, 02 9428 7060, pcessentials@pcfa.org.au

To talk to someone at your participating site:

- Principal Investigator *[Study Site]*: *[Name & contact number]*

If you wish to discuss the study with someone who is not directly involved, particularly in relation to matters concerning complaints about the conduct of the study, or your rights as a participant, you can contact:

Lead HREC Office	Metro South Health and Hospital Services (MSHHS)
Contact Person	HREC Coordinator
Telephone	+61 7 3443 8049
Email	MSH-Ethics@health.qld.gov.au
HREC Reference Number	HREC/2021/QMS/79429

12. The Participant Consent Form

Sign the consent form only after you have made up your mind to take part in this study. You must be provided with a signed and dated copy of the participant information and consent form for your personal record.

Prostate Cancer Survivorship Essentials for Men with Prostate Cancer on Androgen Deprivation Therapy (PCEssentials Hormone Therapy Study)

Participant Consent Form – Clinical Stakeholders

**Title**	Prostate Cancer Survivorship Essentials for Men with Prostate Cancer on Androgen Deprivation Therapy (ADT): An Effectiveness-Implementation Hybrid (Type 1) Trial of a Tele-Based Nurse-Led Survivorship Care Intervention
**Short Title**	*PCEssentials Hormone Therapy Study*
**Coordinating Principal Investigator**	Professor Jeff Dunn AO

Declaration by Participant

- I have read, or have had read to me, and I understand the Participant Information Sheet and Consent Form.
- I understand the purposes, procedures and risks of the research described in the Participant Information Sheet.
- I have had an opportunity to ask questions and I am satisfied with the answers I have received.
- I freely agree to participate in this research study as described and understand that I am free to withdraw at any time during the study without affecting my future health care.
- I understand that I will be given a signed copy of this document to keep for my own records. We may ask you to participate in a future related study, or to obtain additional information or clarification related to your participation in this study. **Please indicate below** whether you are willing to be contacted about any future research studies.
- I agree to the research team using, reproducing, and disclosing audio-recordings as explained in the Participant Information Sheet/Consent Form for Patients.
- I agree to be audio-recorded and understand that, subject to any constraints requested below, recordings may be used in presentations and publications for educational and research purposes.

30-minute Interview

☐ Yes, I agree to be contacted and invited to participate in the interview

☐ No, I do not want to be contacted to be invited to participate in the interview

Future Studies

☐ Yes, I agree to be contacted about future research studies

☐ No, I do not want to be contacted about future research studies

Study Results

☐ Yes, I would like to receive a copy of the study results and acknowledge that these will be provided in aggregate as a summary (individual results will not be available)

☐ No, I do not want a copy of the study results

Participant

Signature ______________________________ Date ________________________

Name (please print) ______________________________

Declaration by senior researcher

I have given a verbal explanation of the study, its procedures and risks and I believe that the participant has understood that explanation.

Signature ______________________________ Date ________________________

Name (please print) ______________________________

Green A, *et al. BMJ Open* 2024; 14:e084412. doi: 10.1136/bmjopen-2024-084412
