# Supplementary material for: Prostate Cancer Survivorship Essentials for men with prostate cancer on androgen deprivation therapy: protocol for a randomised controlled trial of a tele-based nurse-led survivorship care intervention (PCEssentials Hormone Therapy Study)
Source: BMJ Open. 2024 Mar 23;14(3):e084412. doi: 10.1136/bmjopen-2024-084412 (PMC10961537; doi:10.1136/bmjopen-2024-084412)
Supplement: Author's manuscript [file bmjopen-2024-084412.draft_revisions.pdf]

BMJ Open

BMJ Open is committed to open peer review. As part of this commitment we make the peer review history of every article we publish publicly available.

When an article is published we post the peer reviewers' comments and the authors' responses online. We also post the versions of the paper that were used during peer review. These are the versions that the peer review comments apply to.

The versions of the paper that follow are the versions that were submitted during the peer review process. They are not the versions of record or the final published versions. They should not be cited or distributed as the published version of this manuscript.

BMJ Open is an open access journal and the full, final, typeset and author-corrected version of record of the manuscript is available on our site with no access controls, subscription charges or pay-per-view fees (http://bmjopen.bmj.com).

If you have any questions on BMJ Open's open peer review process please email info.bmjopen@bmj.com

BMJ Open

Prostate Cancer Survivorship Essentials for men with prostate cancer on androgen deprivation therapy: Protocol for a randomised controlled trial of a tele-based nurse-led survivorship care intervention (PCEssentials Hormone Therapy Study)

Journal:	*BMJ Open*
Manuscript ID	bmjopen-2024-084412
Article Type:	Protocol
Date Submitted by the Author:	18-Jan-2024

Complete List of Authors:	Green, Anna; University of Southern Queensland, Centre for Health Research<br>Newton, Robert; Edith Cowan University, Exercise Medicine Research Institute<br>Smith, David; The University of Sydney, The Daffodil Centre, a joint venture with Cancer Council NSW<br>Tuffaha, Haitham; The University of Queensland<br>Galvão, Daniel; Edith Cowan University, Exercise Medicine Research Institute<br>Heathcote, Peter; Brisbane Urology Clinic<br>Patel, Manish; University of Sydney<br>Christie, David; Genesiscare; Bond University<br>Egger, Sam; The University of Sydney, The Daffodil Centre, a joint venture with Cancer Council NSW<br>Sara, Sally; Prostate Cancer Foundation of Australia, Nursing Programs<br>Heneka, Nicole; University of Southern Queensland, Centre for Health Research<br>Chambers, Suzanne K; Australian Catholic University<br>Dunn, Jeff; University of Southern Queensland, Centre for Health Research; Prostate Cancer Foundation of Australia

Keywords:	Prostatic Neoplasms, Randomized Controlled Trial, Health Education, Nursing Care, Quality of Life

SCHOLARONE™ Manuscripts

Title: Prostate Cancer Survivorship Essentials for men with prostate cancer on androgen deprivation therapy: Protocol for a randomised controlled trial of a tele-based nurse-led survivorship care intervention (PCEssentials Hormone Therapy Study)

ABSTRACT

Introduction

Androgen deprivation therapy (ADT) is commonly used to treat men with locally advanced or metastatic prostate cancer. Men receiving ADT experience numerous side effects and frequently report unmet supportive care needs. An essential part of quality cancer care is survivorship care. To date an optimal effective approach to survivorship care for men with prostate cancer on ADT has not been described. This protocol describes a randomised trial of tele-based nurse-led survivorship that addresses this knowledge gap:

1. Determine the effectiveness of a nurse-led survivorship care intervention (*PCEssentials*), relative to usual care, for improving health-related quality of life (HR-QoL) in men with prostate cancer undergoing ADT.
2. Evaluate *PCEssentials* implementation strategies and outcomes, including cost-effectiveness, compared to usual care.

Methods and analysis

This is an effectiveness-implementation hybrid (Type 1) trial with participants randomised to one of two arms: i) minimally enhanced usual care; and ii) nurse-led Prostate Cancer Survivorship Essentials (*PCEssentials*) delivered over four tele-based sessions, with a booster session five months after session one. Eligible participants are Australian men with prostate cancer commencing ADT and expected to be on ADT for a minimum of 12 months. Participants are followed-up at 3-, 6-, and 12-months post-recruitment. Primary outcomes are HR-QoL and self-efficacy. Secondary outcomes are psychological distress, insomnia, fatigue, and physical activity. A concurrent process evaluation with participants and study stakeholders will be undertaken to determine effectiveness of delivery of *PCEssentials*.

Ethics and dissemination

Ethics approval was obtained from the Metro South Health HREC (HREC/2021/QMS/79429). All participants are required to provide written informed consent. Outcomes of this trial will be published in peer-reviewed journals. The findings will be presented at conferences and meetings, local hospital departments, participating organisations/clinical services, and university seminars, and communicated at community and consumer-led forums.

Clinical Trial Registration: Australian New Zealand Clinical Trials Registry (ANZCTR) ACTRN12622000025730

Protocol version: 1.6

Protocol date: 16.08.2023

Funding: This work is supported by the National Health and Medical Research Council (NH&MRC) grant number APP2006528.

Word count: 4533

Key words: prostatic neoplasms; randomized controlled trial; health education; nursing care; quality of life

For peer review only - http://bmjopen.bmj.com/site/about/guidelines.xhtml

ARTICLE SUMMARY

Strengths and limitations of this study

- A key strength is the effectiveness-implementation design that allows for a concurrent process evaluation. This will provide immediate implementation data from patient and clinical stakeholders to inform real-world scaling-up of *PCEssentials* if proven effective.
- A cost-utility analysis will provide important economic evaluation data to inform implementation decision-making if the intervention is effective.
- Tele-based interventions are highly acceptable to men with prostate cancer and applicable to geographically dispersed and vulnerable populations with high potential for population-based translation.
- The exclusion of non-English speaking patients, while a pragmatic decision for the trial, will influence the generalisability of study findings to patients from linguistically diverse backgrounds. Should *PCEssentials* be proven effective in this patient population future research addressing this need in non-English speaking populations would be an important next step.
- The intervention takes a preventative health focus to support men at the start of ADT and enhance their personal agency to manage side-effects as treatment progresses.

For peer review only

INTRODUCTION

Prostate cancer (PCa) is the most common cancer diagnosed in Australia (1). While men are living longer following diagnosis, longitudinal research has characterised a subgroup of 35%-40% of men who experience long-term decrements in health-related quality of life (HR-QoL) (2). In particular, men who are on androgen deprivation therapy (ADT) experience consistently poorer physical and mental HR-QoL over the long term (2-6).

While ADT is effective in treating PCa and increasing survival, it is associated with multiple, often debilitating side effects, which manifest as changes in physical, cognitive, social, and sexual functioning (3, 7-9). Iatrogenic effects may include mood disturbances, increased fat mass, body feminisation, cognitive decline, functional impairment, frailty, fatigue, and sexual dysfunction (3, 4, 6-10). ADT also increases the risk of developing new co-morbidities, including cardiovascular conditions, diabetes, sarcopenia, and osteoporosis (11). Compared with men receiving other treatments, those undergoing ADT report poorer HR-QoL and higher levels of psychological distress, including depression, anxiety, relationship changes, cognitive and affective symptoms, and sleep disturbances (3, 4, 6-9, 12). The prevalence of psychological distress in PCa survivors is reported to be between 11%-27% (13), and regardless of other treatments, receiving ADT is predictive of higher distress (12). Further, men undergoing ADT have an increased risk of suicide compared to those who do not, particularly in older men and in the first six-months post diagnosis (14). Unmet supportive care needs are highly prevalent in these men, with unmet physical, psychological, sexual, existential, and informational (12, 15) needs that persist at 15-years post-diagnosis (16). Over one-third (37%) of men with PCa will report at least one long-term unmet supportive care need particularly at the start of treatment when side effects are new or unknown and HR-QoL is first impacted (16). This is of particular concern for men receiving ADT who report feeling unprepared to manage substantial treatment side effects that impact on quality of life (17). Further, despite routine clinical follow-up, men receiving ADT rarely receive tailored person-centred interventions in a timely manner, adversely impacting HR-QoL with poor management of side effects and self-efficacy (12, 15). Men treated with ADT are a vulnerable high-need patient group for whom evidence-based survivorship care is crucial.

Preliminary research on survivorship care for men with PCa

Previous prostate cancer survivorship guidelines published by the American Cancer Society a decade ago (18) were limited by an over reliance on expert opinion and lack of a robust evidence-base (19). Existing survivorship guidelines have also been limited by lack of consumer involvement (20, 21). Our group has contextualised survivorship care for PCa (20, 22, 23) and produced a contemporary survivorship care framework for men with prostate cancer. The resulting *Survivorship Essentials Framework* (Figure 1) proposes holistic survivorship care for men with PCa and was developed by a uniquely inclusive expert clinical and community group (23). The framework has been widely endorsed by key PCa and urological groups in Australia and New Zealand. Based on our survivorship framework, we have developed a new model of care, Prostate Cancer Survivorship Essentials (*PCEssentials*), which integrates evidence-based strategies to improve men's quality of life outcomes after ADT in a men-centred approach, where personal agency intersects with all aspects of care.

We propose an Australian effectiveness-implementation hybrid (Type 1) randomised trial (24) of tele-based nurse-led survivorship care with 236 PCa survivors undergoing ADT. This is the

For peer review only

first such study internationally to address this problem. The proposed study will have two arms: i) minimally enhanced usual care; and ii) nurse-led Prostate Cancer Survivorship Essentials (*PCEssentials*) delivered over four tele-based sessions, with a subsequent booster session five months after the first session. In accordance with a Type 1 hybrid trial, a concurrent process evaluation, guided by the Conceptual Framework for Implementation Outcomes (25), will be undertaken to determine effectiveness of the *PCEssentials* intervention delivery, and the potential for implementation of the intervention at scale.

Aims

Aim 1: Determine the effectiveness of a nurse-led survivorship care intervention (*PCEssentials*), relative to usual care, for improving HR-QoL in men with PCa undergoing ADT.

Aim 2: Evaluate *PCEssential*s implementation strategies and outcomes, including cost-effectiveness of *PCEssentials*, with respect to usual care, as well as acceptability, adoption, appropriateness, feasibility, fidelity, penetration, and sustainability.

Primary hypothesis

We hypothesise that *PCEssentials* will be more cost-effective than usual care. Furthermore, relative to men receiving usual care at 3-, 6-, and 12-months after recruitment, men who receive *PCEssentials* will have: i) higher HR-QoL; ii) increased self-efficacy; iii) less psychological distress; and iv) improved sleep and lower fatigue.

METHODS AND ANALYSIS

Study design

A Type 1 effectiveness-implementation hybrid randomised trial (24) of a nurse-led survivorship care intervention (*PCEssentials*), relative to usual care, for improving HR-QoL in men with PCa undergoing ADT. A concurrent process evaluation will determine the effectiveness of intervention delivery, and the potential for implementation at scale. The study design has been guided by the CONSORT criteria (26).

There are four key study time-points:

- T1 – Baseline: prior to randomisation
- T2 – 3 months post-recruitment
- T3 – 6 months post-recruitment
- T4 – 12 months post-recruitment

This study will be undertaken in accordance with the National Statement on Ethical Conduct in Human Research (2007 – updated 2018) (27) and the Australian Code for the Responsible Conduct of Research (2018) (28).

Research population

There are two research populations for this study:

1. Patient participants (n=236): Australian men (aged 18 years or over) diagnosed with PCa commencing, or within 3 months of having commenced, ADT.

For peer review only

2. Process evaluation participants (n=148): Study stakeholders (n=30) who are directly involved in study delivery and/or translation into clinical practice, including participating service managers, recruiting clinicians, nurses delivering the intervention, health professionals; and patient participants in the intervention group (n=118). While all participants in the intervention group will complete program acceptability assessments at two study time-points (T1 and T3), approximately 20 of these patient participants will be purposively selected/invited to take part in a semi-structured interview (T3) to explore their experiences of the intervention. Purposive sampling will ensure a patient subgroup with maximum diversity (e.g., based on age, background, location, partnered or un-partnered). We anticipate reaching data saturation for the process evaluation with this number of participants.

Inclusion criteria

Men recruited to the study will: i) have been diagnosed with PCa and be commencing, or within 3 months of having commenced ADT, and expected (based on clinical information) to be on ADT for a minimum continuous period of 12 months; ii) are able to read and speak English; iii) are able to give written informed consent; iv) have no previous history of head injury, dementia, or psychiatric illness; v) have no other concurrent cancer; and vi) have mobile and/or landline phone access.

Exclusion criteria

Men with castrate resistant and confirmed metastatic disease are excluded on the basis of having progressive and incurable disease that may rapidly progress and the study doesn't meet their needs.

Research project setting/location

There are multiple recruitment settings through clinicians in major treatment centres across Australia and by patient self-referral. Study information for patient self-referral is disseminated through investigator networks.

Research project procedures

1. Intervention

Following referral (clinician or self) to the study team, research staff screen potential participants for eligibility and conduct an informed consent process (Figure 2). Once eligibility is confirmed, and written informed consent received, participants receive the baseline assessments (T1) via mail. Upon return of T1 assessments, the study team randomises participants into the intervention or minimally enhanced usual care ('usual care') group.

Men randomised to the intervention group commence the *PCEssentials* intervention, a five-session psychoeducation program delivered by trained Prostate Cancer Specialist Nurses via mobile and/or landline telephone. This includes four sessions over three months and a booster session at five months after the first session. Men in the intervention group are also

For peer review only

be offered a home-based exercise program and encouraged to seek at least one planning session with an Accredited Exercise Physiologist (AEP).

Men in the usual care group receive their standard management, minimally enhanced with a package of evidence-based resources.

Men in both groups will continue to attend their standard PCa related care, and complete study assessments at 3-, 6-, and 12-months post recruitment.

2. Process evaluation

A mixed methods approach will examine the elements of the Conceptual Framework for Implementation Outcomes (25) as they relate to the *PCEssentials* intervention, namely: acceptability, adoption, appropriateness, penetration, feasibility, fidelity, and sustainability. To assess program acceptability and feasibility, clinical stakeholders involved in the delivery or oversight of the program will be invited by the partner investigator at each site to participate in: i) a short online survey when recruitment commences and ends at the site; and ii) a semi-structured interview when recruitment ends. Invitations will be sent to eligible clinical stakeholders via email, with written informed consent sought prior to surveys/interviews being undertaken.

Recruitment

Recruitment is undertaken through clinicians in major treatment centres across Australia. With patient permission, clinicians are asked to directly refer eligible patients to the study team who then proceed with an informed consent process. A two-phase consent process is used for patient participants who are referred by a clinician: i) written, or verbal, where appropriate, permission to provide the patient's contact details to the study team for follow-up; and ii) written informed consent to take part in the study.

Additionally, men may self-refer having identified the study through media promotion and PCa support groups. In this case, potential participants contact the research team directly and provide written informed consent after being screened for eligibility.

Based on our experience with previous interventions in similar cohorts (29-31), and active participation of our project partners, we anticipate a recruitment period of 18 months to randomise 236 patients.

Randomisation

Randomisation to study group condition occurs following receipt of baseline assessments (Figure 2). Randomisation occurs in varying block sizes of four, six and eight (to ensure an unpredictable allocation sequence with equal numbers of men in each treatment group at the completion of each block) with no stratification factors. The randomisation sequence is undertaken by the project manager and concealed from investigators. Project staff tracking assessments (data analysts) will be blinded to condition.

Research project process

1. Patients

Patient-reported outcomes and experience assessments are completed at each study time-point (T1-T4). Following informed consent, participants are sent the T1 assessments for completion. Upon receipt of completed T1 assessments by the research team, participants are randomised into either i) minimally enhanced usual care (control); or ii) nurse-led survivorship care: *PCEssentials* (intervention group).

i) Minimally enhanced usual care

Standard management, minimally enhanced with evidence-based patient education materials about the use of ADT to treat PCa, and information about free telephone-based cancer information and support services in the participant's home state.

ii) Nurse-led survivorship care (*PCEssentials*)

The nurse-led intervention is telephone delivered over five sessions by trained Prostate Cancer Specialist Nurses, guided by manualised intervention protocols, and supervised by an experienced prostate cancer specialist nurse and a health psychologist with extensive experience in prostate cancer supportive care. The intervention includes five modules covering: psycho-education with tailored distress management strategies; decision support; treatment education with self-management and skills training for symptom effects, including exercise/physical activity resources and support; and communicating with health professionals including a referral pathway to their general practitioner for chronic disease management.

A problem solving approach that supports personal agency underpins each component (20), with the first four sessions to be delivered by telephone over three months, and an additional booster session five months after the initial session module has been completed. A problem-solving approach (32) that is responsive to masculine models of coping and life stage was chosen as the underlying mechanism of support to enhance personal agency.

Men with PCa experience improved psychological outcomes when they engage in approach coping that addresses the threats associated with their cancer (33), and active problem solving is consistent with male values around strength, self-reliance and action (34). Problem-solving therapy (PST) has been found to be effective in reducing depression and disability in older people ($\geq$60 years of age) with chronic illness (32). Our intervention targets include major challenges identified by men (e.g., psychological distress, disease and treatment effects, communicating with health professionals) and applies PST to enhance men's personal agency in defining and formulating the nature of their specific problems, generating potential solutions, systematically evaluating possible consequences of solutions and selecting an appropriate solution, and monitoring solution outcomes. A self-help survivorship resource that addresses key PCa-related challenges with evidence-based coping strategies is provided and this connects directly to the nurse-led intervention session content (35).

Distress screening and problem identification occurs at each session using the Distress Thermometer and is integrated with distress and symptom management strategies (36). The booster session checks participant progress, reinforces self-management skills, and troubleshoots concerns that may have persisted.

For peer review only

A home-based physical exercise program is offered, where men are encouraged to seek at least one planning session with an Accredited Exercise Physiologist (AEP) within their treatment team, accessed by telephone or internet. The nurse specialist encourages exercise maintenance, including aerobic and resistance training as per the Australian Exercise Medicine for Cancer guidelines with referral to an AEP, if required (37).

Men have identified that the Prostate Cancer Specialist Nurse/clinical nurse is highly acceptable as the provider of survivorship care, an approach described as the most efficient in terms of use and resources and being suitable for most care settings (38). Tele-based interventions are also highly acceptable to men with PCa (85% consent rate (22)), are accessible for patients who are very unwell (39), have been shown to be an effective delivery method for problem solving therapy (32), and in advanced disease show low attrition rates compared to face-to-face delivery (20). This delivery method is also applicable to geographically dispersed and vulnerable populations with high potential for population-based translation.

2. Process evaluation

Process assessments are collected via: i) surveys using the Program Acceptability: Acceptability of Intervention Measure (AIM), Intervention Appropriateness Measure (IAM) and Feasibility of Intervention Measure (FIM) (40) at T1 and T3 (patient participants) and when recruitment commences and ends (other stakeholders), as well as the Working Alliance Inventory – Short Revised (WAI-SR) scale (41) at T3 (intervention group patient participants only); ii) semi-structured interviews with stakeholders at T3 (patient participants) and when recruitment ends (other stakeholders); and iii) intervention fidelity and adherence assessments at multiple study time-points, to identify barriers and facilitators to implementation, and determine if high intervention fidelity is achieved.

Research outcomes and measurement tools

Previously validated and reliable patient-reported outcome assessments are administered by mail to men at four-time points: baseline/recruitment (T1), 3 months (T2), 6 months (T3), and 12 months (T4) after recruitment. Primary outcomes are HR-QoL and self-efficacy. Secondary outcomes include global psychological distress, insomnia, fatigue, and life satisfaction. Demographic moderators/disease variables (e.g., cancer grade, stage, time since diagnosis, time since treatment) and a health service use diary are self-reported. Assessments are self-report pen and paper.

Primary outcomes

Health-Related Quality of Life: The *Functional Assessment of Cancer Therapy – Prostate (FACT-P)* (42) assesses men's disease-specific quality of life across five domains: physical, social/family, emotional, functional well-being, and PCa specific concerns (42). The *Assessment of Quality of Life* (AQoL-8D) instrument is used to derive health utility scores and general HR-QoL among patients. This tool has increased measurement sensitivity to psychosocial elements of health compared to other instruments, since it comprises five psychosocial dimensions (mental health, happiness, coping, relationships, and self-worth) and

For peer review only

three physical dimensions (independent living, pain, and senses) (43). The physical function subscale from the *Medical Outcomes Study Short-Form-36* (SF-36) questionnaire will be used as an indicator of patient-related physical functioning QoL (44). We recently reported improvements in physical function in PCa patients with advanced disease and bone metastases following an exercise intervention using this measure, and in those on ADT with localised disease (45).

Self-efficacy: The 11-item *Cancer Survivorship Self-Efficacy Scale* (CS-SES) (46) assesses self-efficacy to manage problems arising from cancer and its treatment specifically.

Secondary outcomes

Psychological distress: The Generalized Anxiety Disorder (GAD-7) scale (47) and the depression subscale of the Patient Health Questionnaire (PHQ-9) (48) will measure psychological distress. The seven item GAD-7 scale screens for, and assesses the severity of, generalised anxiety disorder in clinical practice and research. The nine item PHQ-9 scale screens for, and assesses the severity of, depression and includes a specific item on suicidal ideation.

Insomnia: The *Insomnia Severity Index* (ISI) is the worldwide standard, seven-item self-report measure to evaluate: (a) severity of sleep-onset, (b) sleep maintenance, (c) early morning awakening problems, (d) satisfaction with current sleep pattern, (e) interference with daily functioning, (f) noticeability of impairment attributed to the sleep problem, and (g) level of distress caused by the sleep problem (49).

Fatigue: The *Multidimensional Fatigue Symptom Inventory-Short Form* (MFSI-SF) (50) assesses general fatigue, physical fatigue, emotional fatigue, mental fatigue, and vigour.

Physical activity/exercise: Godin-Shephard Leisure-Time Physical Activity Questionnaire (GSLTPAQ) (51), modified to include questions on resistance training, reflecting current best practice in exercise intervention trials for men with PCa (52), will assess physical activity.

Process evaluation

Program acceptability: The Acceptability of Intervention Measure (AIM), Intervention Appropriateness Measure (IAM) and Feasibility of Intervention Measure (FIM) (33) is a short self-reported assessment that is collected at T1 and T3 (patient participants) to determine patients' experiences of the study from recruitment to six months post recruitment. For patient participants, this is included in the self-reported study assessments mailed to them at T1 and T3. The therapeutic alliance between patients in the intervention group and the nurses delivering the intervention will also be assessed by the 12 item Working Alliance Inventory – Short Revised (WAI-SR) (41). This will be included in the self-reported study assessments mailed to patient participants at T3.

All other study stakeholders receive the same assessments as an online survey when recruitment starts and ends to determine their study experience.

For peer review only

Interviews: Semi-structured interviews exploring the constructs of the Conceptual Framework for Implementation Outcomes (25) will be undertaken to determine effectiveness of the *PCEssentials* intervention delivery, and the potential for implementation of the intervention at scale. The interview question route informed by the literature is included in Supplementary File 1.

Statistical considerations and data analysis

Recent meta-analyses conclude that individually focussed psychological interventions should produce improvements in psychological distress of at least a medium effect size (d=0.40) that will be clinically meaningful (53). To see an effect of this size or greater in our primary outcome, psychological distress at 12 months, with 80% power and alpha=0.05, we will require 99 participants in each group to complete the intervention. Assuming 15% attrition, we will recruit 236 patients to the study (118 patients per group).

1. Intervention effectiveness

The study is a two-arm randomised controlled trial with repeated assessments across time and with continuous primary outcome variables. Recruitment bias will be assessed by comparing sociodemographic and clinical variables for consenters with non-consenters using t-tests (or Mann-Whitney U tests) for continuous variables and chi-square tests for categorical variables. Possible differential attrition will be assessed by comparing baseline characteristics of drop-outs and continuing participants using t-tests (or Mann-Whitney U tests if appropriate) for continuous variables and chi-square tests for categorical variables. Intention-to-treat analyses will be conducted. Between-group mean differences in change from baseline outcome scores at 3, 6 and 12 months will be analysed by fitting mixed effects regression models. Intervention (intervention/usual care) will be included as the main effect. Indicators for participants will be included as a random effect to account for the non-independence of repeated observations from the same individual. Sensitivity analysis will assess the effects of attrition. Mixed effects models with maximum likelihood estimation minimise bias that may arise from ignoring missing observations, and use all available data, thereby maximising statistical power to detect effects. The mean and 95% confidence interval will be calculated for satisfaction with the intervention. Missing data will be examined for patterns of missingness and addressed with the appropriate multiple imputation methods, if required. The investigator team includes a dedicated biostatistician who will undertake analyses.

2. Process evaluation

Process evaluation assessments will be analysed using a combination of descriptive statistics (measures of program acceptability), and deductive directed content analysis (semi-structured interviews) (54). Joint display tables will facilitate the data integration process and facilitate the drawing of inferences from the integrated data (55).

3. Cost-utility analysis

A cost-utility analysis of the intervention relative to minimally enhanced usual care from both healthcare payer and societal perspectives will be conducted alongside the *PCEssentials* trial.

For peer review only

Costs will be obtained by identifying, measuring and valuing the health resources used. At baseline, participants are given a health service use diary to record direct health resources utilised (e.g., GP visits, treatments, and hospitalisations), as well as out-of-pocket expenses and indirect costs (e.g., productivity loss). The diaries will also be collected during the T2, T3 and T4 assessments. Healthcare resources will be valued using unit prices from standard costing resources such as the Medicare Benefits Schedule and relevant Australian award wages. Quality adjusted life years (QALY) gained will be estimated, which is a measure of a patient's life expectancy, weighted by his health-related quality of life (i.e., utility score) measured using the AQoL-8D at baseline, 3, 6 and 12 months. A multivariate generalised linear model will be used to adjust for differences in baseline AQoL-8D scores, demographics and disease classifications. The incremental cost-effectiveness ratio (ICER) will be calculated, which is the difference in mean costs divided by the difference in mean QALYs. Non-parametric bootstrapping will be used to characterise uncertainty around the ICER. If the intervention appears to be cost-effective, we will calculate the expected value of implementation, which is the net monetary benefit of the intervention (i.e., monetary benefits – costs) multiplied by the population of PCa patients expected to benefit from the intervention and adjusted by various patients' adherence and clinicians' uptake rates. Uptake rates will be obtained from a formal elicitation exercise and will inform a Bass model to forecast diffusion (i.e., implementation over time) (56).

Patient and public involvement statement

This research project was developed through a collaboration between the University of Southern Queensland and the Prostate Cancer Foundation of Australia as the co-lead organisations. The Prostate Cancer Foundation of Australia is a broad-based community organisation and the peak national body for PCa in Australia. Patient/public involvement in the research has been carried through the conceptualisation and design of the study and *PCEssentials* intervention, to recruitment and delivery of the intervention through this partnership. Consumer and clinical representatives have contributed to project steering committees and development of the intervention. The Prostate Cancer Foundation of Australia will assist with dissemination of study results through their consumer and clinical stakeholder network ensuring future patient/public engagement.

ETHICS AND DISSEMINATION

Ethics approval for this study was obtained from the Metro South Health Human Research Ethics Committee (HREC/2021/QMS/79429).

Safety considerations

Experienced Prostate Cancer Specialist Nurses ('intervention nurses') are responsible for the delivery of the intervention. Intervention nurses receive: i) additional training in the study-specific protocol and *PCEssentials* intervention; ii) an intervention manual detailing session content and activities; and iii) weekly supervision and debriefing by study investigators with extensive experience in the delivery of the prostate cancer supportive care. All other study staff will also receive protocol specific and research processes training.

Data management and monitoring

For peer review only

Written, informed consent is obtained from each patient and clinical stakeholder prior to study enrolment and any study activities being undertaken. Patient participants are given a unique participant identification code (ID). This ensures that all identifying data can be removed before data analysis commences. This project ID enables the research team to manage the data in a confidential manner. The master list linking identifying participant information and ID number is maintained in a locked cabinet, separate from the participant database at the Prostate Cancer Foundation of Australia. All data collected for each participant is kept in a participant file (identified by ID number only) which contains the Case Report Forms, any corrected and amended data, copies of adverse event reports, file notes etc. All study files are stored in accordance with Good Clinical Practice guidelines.

Form tracking is via participant ID number only. The participant database is stored on a password-protected hard drive maintained by the study investigators. Data will be analysed by ID number only. All information presented in dissemination will be de-identified group data that will not allow the identification of individual participants.

Treatment fidelity

The intervention is manualised and intervention nurses complete a checklist of components delivered at each session. Throughout the study, sessions are audiotaped and 15% of sessions will be reviewed to assess adherence to protocol. The intervention nurses are supervised by an investigator who is a qualified psychologist with oversight on treatment fidelity monitoring according to NIH guidelines (57).

Ethical considerations

There are two potential risks for participants related to the intervention: (i) minor psychological distress may be experienced by some participants while discussing issues relating to treatment, side-effects, and psychosocial impact during the intervention; (ii) side-effects arising from changes in physical activity (such as muscle soreness) if participants choose to take part in the exercise component of the intervention. However, the psychological distress that may be experienced by some participants will be no greater than that experienced when discussing issues related to PCa management with their doctor. Similarly, the side-effects that may be experienced by some participants while in the process of the exercise component are likely to be no greater than the risks of day-to-day living as people can undertake changes in their level of physical activity.

Adverse events will be recorded by the research team immediately upon their notification. Should any adverse or serious adverse events occur, the research team will report to the governing ethics committee, review relevant risk assessments, aim to mitigate future risk of adverse events and provide the appropriate duty of care to the participant/s concerned.

Risk mitigation

Psychological distress will be minimised by identifying those individuals who are experiencing high distress and tailoring the intervention to specifically manage stress in these individuals. The intervention specialist nurses are trained to assess psychological distress and to manage this during the nurse-led intervention. Participants who request additional psychological

For peer review only

support beyond the intervention will be referred to additional sources including the Prostate Cancer Foundation of Australia Telenursing Service (direct referral to the telenursing service manager who is not an intervention nurse), Beyond Blue, Lifeline and/or other relevant local services. Medical management of participants will be managed as per their usual care.

Dissemination

Outcomes of this trial will be published in peer-reviewed journals, and the findings presented at national and international conferences and meetings. Findings will also be communicated at community and consumer-led forums and presented at local hospital departments, participating organisations/clinical services, and university seminars. This study is designed so that outputs are translatable into practice to improve the health and well-being of men with PCa receiving ADT. Should it prove effective, our intervention may be utilised in a range of settings, including broad-reach tele-based support programs; and through support services across Australia that are conducted by state Cancer Councils and the Prostate Cancer Foundation of Australia, as well as through similar support service infrastructures internationally.

CONCLUSION

Men with PCa receiving ADT are a vulnerable high-need patient group. As yet an effective way to deliver holistic survivorship care to improve HR-QoL in this patient population has not yet been identified. The study will provide effectiveness and implementation data to address this knowledge gap and inform the potential for implementation of *PCEssentials* at scale.

For peer review only

Author contributions

JD, SC, NH, AG, RN, DS, HT, DG, PH, MP, DC, SE and SS contributed to study conception or study design. SS, SC, DG, RN, NH, JD and AG were on the steering committee that developed the intervention. HT designed the economic component of the study. JD, SC, NH, AG, RN, DS, HT, DG, PH, MP, DC, SE, and SS provided substantial input into the development of the protocol or revising it critically for important intellectual content. AG drafted the manuscript with contributions from RN, DS, HT, DG, PH, MP, DC, SE, SS, NH, SC, JD. All authors contributed to, read and approved the final manuscript.

Funding statement

This work is supported by the National Health and Medical Research Council (NH&MRC) grant number APP2006528.

Competing interests statement

No conflicts of interest.

For peer review only

Figure 1. Prostate Cancer Survivorship Essentials Framework(23)

Figure 2. Study Diagram

For peer review only

References

1. Australian Institute of Health and Welfare. Cancer data in Australia [online]. 2023. https://www.aihw.gov.au/reports/cancer/cancer-data-in-australia/contents/overview-of-cancer-in-australia-2023 (accessed 13 December 2023).

2. Ralph N, Ng SK, Zajdlewicz L, et al. Ten-year quality of life outcomes in men with prostate cancer. Psycho-Oncology 2020;29(2):444-9.

3. Chipperfield K, Fletcher J, Millar J, et al. Predictors of depression, anxiety and quality of life in patients with prostate cancer receiving androgen deprivation therapy. Psycho-Oncology 2013;22(10):2169-76.

4. Nguyen C, Lairson DR, Swartz MD, et al. Risks of Major Long-Term Side Effects Associated with Androgen-Deprivation Therapy in Men with Prostate Cancer. Pharmacotherapy 2018;38(10):999-1009.

5. Mazariego CG, Egger S, King MT, et al. Fifteen year quality of life outcomes in men with localised prostate cancer: population based Australian prospective study. BMJ 2020;371:m3503.

6. Smith DP, King MT, Egger S, et al. Quality of life three years after diagnosis of localised prostate cancer: population based cohort study. BMJ 2009;339:B4817.

7. Casey RG, Corcoran NM, Goldenberg SL. Quality of life issues in men undergoing androgen deprivation therapy: a review. Asian Journal of Andrology 2012;14(2):226-31.

8. Edmunds K, Tuffaha H, Galvão DA, et al. Incidence of the adverse effects of androgen deprivation therapy for prostate cancer: a systematic literature review. Supportive Care in Cancer 2020;28(5):2079-93.

9. Rhee H, Gunter JH, Heathcote P, et al. Adverse effects of androgen-deprivation therapy in prostate cancer and their management. BJU International 2015;115(Suppl 5):3-13.

10. Australian Cancer Network Management of Metastatic Prostate Cancer Working Party. *Clinical Practice Guidelines for the Management of Locally Advanced and Metastatic Prostate Cancer*. Sydney: Cancer Council Australia and Australian Cancer Network, 2010.

11. Ng HS, Koczwara B, Roder D, et al. Development of comorbidities in men with prostate cancer treated with androgen deprivation therapy: an Australian population-based cohort study. Prostate Cancer and Prostatic Diseases 2018;21(3):403-10.

12. Paterson C, Primeau C, Howard N, et al. Experiences of Unmet Need and Access to Supportive Care for Men Receiving Androgen Depriviation Therapy for Prostate Cancer: A Bi-national Study. Seminars in Oncology Nursing 2020;36(4):151049.

13. Watts S, Leydon G, Birch B, et al. Depression and anxiety in prostate cancer: a systematic review and meta-analysis of prevalence rates. BMJ Open 2014;4(3):e003901.

14. Carlsson S, Sandin F, Fall K, et al. Risk of suicide in men with low-risk prostate cancer. European Journal of Cancer 2013;49(7):1588-99.

15. Paterson C, Kata SG, Nandwani G, et al. Unmet Supportive Care Needs of Men With Locally Advanced and Metastatic Prostate Cancer on Hormonal Treatment: A Mixed Methods Study. Cancer Nursing 2017;40(6):497-507.

16. Mazariego CG, Juraskova I, Campbell R, et al. Long-term unmet supportive care needs of prostate cancer survivors: 15-year follow-up from the NSW Prostate Cancer Care and Outcomes Study. Supportive Care in Cancer 2020;28(11):5511-20.

For peer review only

17. Gray RE, Wassersug RJ, Sinding C, et al. The experiences of men receiving androgen deprivation treatment for prostate cancer: a qualitative study. The Canadian Journal of Urology 2005;12(4):2755-63.

18. Skolarus TA, Wolf AMD, Erb NL, et al. American Cancer Society prostate cancer survivorship care guidelines. CA Cancer J Clin 2014;64(4):225-49.

19. Narayan V, Harrison M, Cheng H, et al. Improving research for prostate cancer survivorship: A statement from the Survivorship Research in Prostate Cancer (SuRECaP) working group. Urol Oncol 2020;38(3):83-93.

20. Dunn J, Ralph N, Green A, et al. Contemporary consumer perspectives on prostate cancer survivorship: Fifty voices. Psycho-Oncology 2020;29(3):557-63.

21. Armstrong MJ, Bloom JA. Patient involvement in guidelines is poor five years after institute of medicine standards: review of guideline methodologies. Res Involv Engagem 2017;3:19.

22. Crawford-Williams F, March S, Goodwin BC, et al. Interventions for prostate cancer survivorship: A systematic review if reviews. Psycho-Oncology 2018;27(10):2339-48.

23. Dunn J, Green A, Ralph N, et al. Prostate Cancer Survivorship Essentials Framework: Guidelines for Practitioners. BJU Int 2021;128(Suppl 3):18-29.

24. Curran GM, Bauer M, Mittman B, et al. Effectiveness-implementation hybrid designs: combining elements of clinical effectiveness and implementation research to enhance public health impact. Med Care 2012;50(3):217-26.

25. Proctor E, Silmere H, Raghavan R, et al. Outcomes for implementation research: Conceptual distinctions, measurement challenges, and research agenda. Administration and Policy in Mental Health Services Research 2011;38(2):65-76.

26. Altman DG, Schulz KF, Moher D, et al. The Revised CONSORT Statement for Reporting Randomized Trials: Explanation and Elaboration. Annals of Internal Medicine 2001;134(8):663-94.

27. The National Health and Medical Research Council, The Australian Research Council, Universities Australia. *National Statement in Ethical Conduct in Human Research 2007*. Canberra: Commonwealth of Australia, 2007.

28. The National Health and Medical Research Council, The Australian Research Council, Universities Australia. *Australian Code for the Responsible Conduct of Research 2018*. Canberra: Commonwealth of Australia, 2018.

29. Chambers SK, Ferguson M, Gardiner R, et al. ProsCan for Men: Randomised controlled trial of a decision support intervention for men with localised prostate cancer. BMC Cancer 2008;8(1):207.

30. Chambers SK, Occhipinti S, Schover L, et al. A randomised controlled trial of a couples-based sexuality intervention for men with localised prostate cancer and their female partners. Psycho-Oncology 2015;24(7):748-56.

31. Zajdlewicz L, Hyde M, Lepore S, et al. Health-related quality of life after diagnosis of locally-advanced or advanced prostate cancer: A longitudinal study. Cancer Nursing 2017;40(5):412-9.

32. Kiosses DN, Alexopoulos GS. Problem-Solving Therapy in the Elderly. Current Treatment Options in Psychiatry 2014;1(1):15-26.

33. Roesch SC, Adams L, Hines A, et al. Coping with prostate cancer: a meta-analytic review. Journal of Behavioral Medicine 2005;28(3):281-93.

For peer review only

34. Chambers S, Hyde M, Oliffe J, et al. Measuring Masculinity in the Context of Chronic Disease. Psychology of Men & Masculinity 2015;17(3):228-42.

35. Chambers S. Facing the Tiger: A Survivorship Guide for Men with Prostate Cancer and their Partners. Brisbane, Australia: Australian Academic Press 2020.

36. Chambers SK, Zajdlewicz L, Youlden DR, et al. The validity of the distress thermometer in prostate cancer populations. Psycho-Oncology 2014;23(2):195-203.

37. Hayes SC, Newton RU, Spence RR, et al. The Exercise and Sports Science Australia position statement: Exercise medicine in cancer management. Journal of Science and Medicine in Sport 2019;22(11):1175-99.

38. Shapiro CL. Cancer Survivorship. The New England Journal of Medicine 2018;379(25):2438-50.

39. Chambers S, Hyde M, Smith D, et al. New Challenges in Psycho-Oncology Research III: A Systematic Review of Psychological Interventions for Prostate Cancer Survivors and Their Partners: Clincial and Research Implications. Psycho-Oncology 2017;26(7):873-913.

40. Weiner BJ, Lewis CC, Stanick C, et al. Psychometric assessment of three newly developed implementation outcome measures. Implementation Science 2017;12(1):108.

41. Hatcher RL, Gillaspy JA. Development and validation of a revised short version of the working alliance inventory. Psychotherapy Research 2006;16(1):12-25.

42. Cella DF, Tulsky DS, Gray G, et al. The Functional Assessment of Cancer Therapy scale: development and validation of the general measure. Journal of Clinical Oncology 1993;11(3):570-9.

43. Richardson J, Elsworth G, Iezzi A, et al. *Increasing the sensitivity of the AQoL inventory for evaluation of interventions affecting mental health*. Melbourne, Australia: Monash University, Business and Economics, Centre for Health Economics, 2011.

44. Ware JE Jnr, Gandek B. Overview of the SF-36 Health Survey and the International Quality of Life Assessment (IQOLA) Project. Journal of Clinical Epidemiology 1998;51(11):903-12.

45. Galvão DA, Taaffe DR, Spry N, et al. Combined resistance and aerobic exercise program reverses muscle loss in men undergoing androgen suppression therapy for prostate cancer without bone metastases: a randomized controlled trial. Journal of Clinical Oncology 2010;28(2):340-7.

46. Foster C, Breckons M, Cotterell P, et al. Cancer survivors' self-efficacy to self-manage in the year following primary treatment. Journal of cancer survivorship: research and practice 2015;9(1):11-9.

47. Spitzer RL, Kroenke K, Williams JB, et al. A brief measure for assessing generalized anxiety disorder: the GAD-7. Archives of Internal Medicine 2006;166(10):1092-7.

48. Kroenke K, Spitzer RL, Williams JB. The PHQ-9: validity of a brief depression severity measure. J Gen Intern Med 2001;16(9):606-13.

49. Bastien CH, Vallieres A, Morin CM. Validation of the Insomnia Severity Index as an outcome measure for insomnia research. Sleep Medicine 2001;2(4):297-307.

50. Stein KD, Jacobsen PB, Blanchard CM, et al. Further validation of the multidimensional fatigue symptom inventory-short form. Journal of Pain and Symptom Management 2004;27(1):14.

51. Godin G. The Godin-Shephard leisure-time physical activity questionnaire. The Health Fitness Journal of Canada 2011;4(1):18-22.

For peer review only

52. Galvão DA, Taaffe DR, Hayne D, et al. Weight loss for overweight and obese patients with prostate cancer: a study protocol of a randomised trial comparing clinic-based versus Telehealth delivered EXercise and nutrition intervention (the TelEX trial). BMJ Open 2022;12(6).

53. Faller H, Scuhler M, Richard M, et al. Effects of Psycho-Oncologic Interventions on Emotional Distress and Quality of Life in Adult Patients with Cancer: Systematic Review and Meta-Analysis. Journal of Clinical Oncology 2013;31(6):782-93.

54. Hsieh HF, Shannon SE. Three approaches to qualitative content analysis. Qualitative health research 2005;15(9):1277-88.

55. Guetterman TC, Fetters MD, Creswell JW. Integrating Quantitative and Qualitative Results in Health Science Mixed Methods Research through Joint Displays. Ann Fam Med 2015;13(6):554-61.

56. Grimm SE, Stevens JW, Dixon S. Estimating Future Health Technology Diffusion Using Expert Beliefs Calibrated to an Established Diffusion Model. Value Health 2018;21(8):944-50.

57. Bellg AJ, Borrelli B, Resnick B, et al. Enhancing treatment fidelity in health behaviour change studies: best practices and recommendations from the NIH Behaviour Change Consortium. Health Psychol 2004;23(5):443-51.

For peer review only

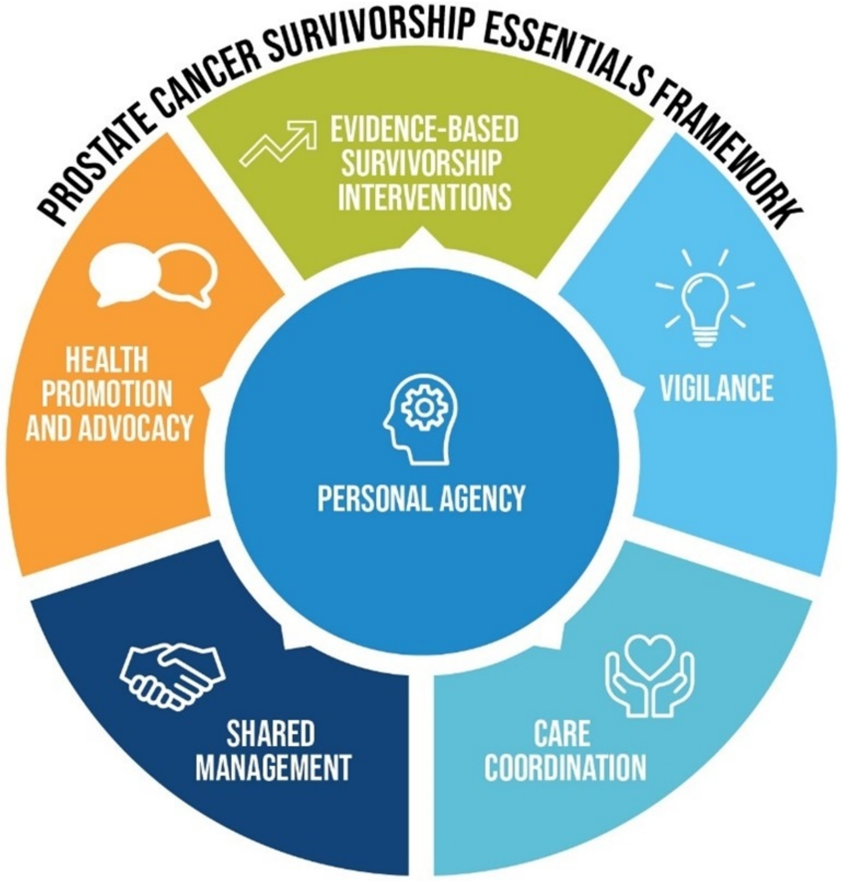

Figure 1. Prostate Cancer Survivorship Essentials Framework23

105x105mm (220 x 220 DPI)

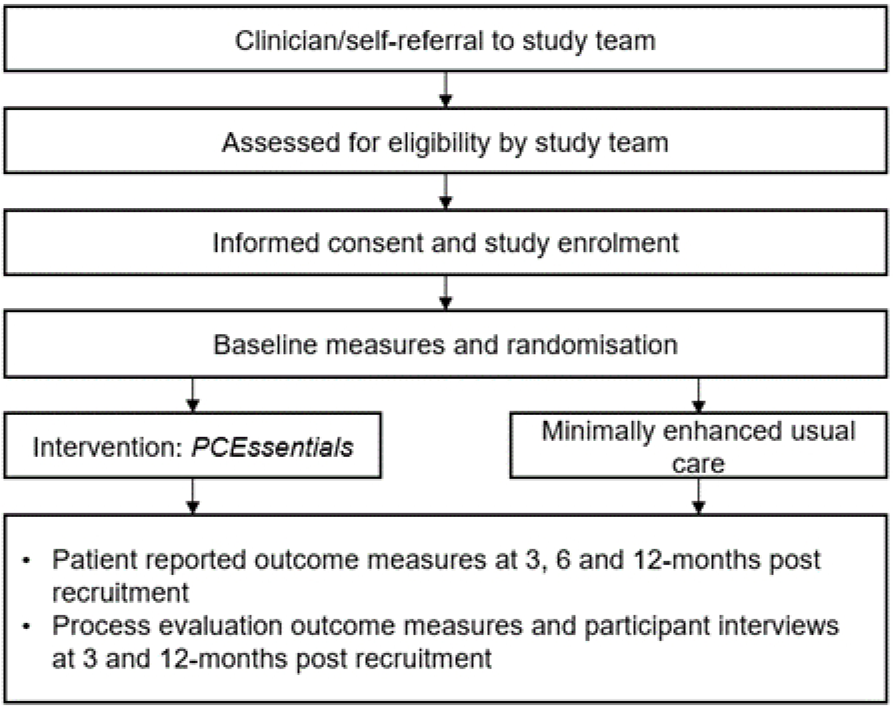

Figure 2. Study Diagram

322x243mm (38 x 38 DPI)

Supplementary File 1. Question route for interviews

Patient Question Route – Semi-Structured interviews; based on relevant Conceptual Framework for Implementation Outcomes[1]

Italics: Question prompts

**Construct/Outcome**	**Questions**
How an individual feels about taking part in an intervention	How did you feel about taking part in *PCEssentials*?<br>• *When you first heard about it*<br>• *While you were taking part*
The extent to which the participant understands the intervention, and how the intervention works	How would you describe what *PCEssentials* was about?
The participant's confidence that they can perform the behaviour(s) required to participate in the intervention	How confident were you that you could do what you needed to take part in *PCEssentials*?<br>• *Access and use resources*<br>• *Contact the intervention nurse*<br>• *Complete the homework*
The perceived amount of effort that is required to participate in the intervention	Do you think *PCEssentials* is easier or harder than coming to the hospital/clinic for care?<br>• *In what way is it easier/harder?*
The extent to which benefits, profits, or values must be given up to engage in an intervention	Did you feel you had to give anything up/miss out on anything to take part in *PCEssentials*? (out of pocket expenses, quality of care)<br>• *Can you give some examples?*<br>• *Do you think the quality of care you received/costs was the same as coming to the hospital/clinic for care?*
The extent to which the intervention has good fit with an individual's value system	Does this type of virtual care meet your needs?<br>• *Why or why not?*<br>• *What could be changed to meet your needs?*<br>• *What was it that really helped meet your needs?*
The extent to which the intervention is perceived as likely to achieve its purpose	Looking back at *PCEssentials* since you started, how effective do you think it is overall?<br>• *In what way is it effective/not effective?*<br>Can you give me an example of something you really liked/disliked about *PCEssentials*?<br>How could *PCEssentials* be improved?<br>Is there anything else you wanted to say about *PCEssentials*?

For peer review only

Clinical Stakeholder Question Route – Semi-Structured interviews; based on relevant Conceptual Framework for Implementation Outcomes[1]

Italics: Question prompts

Construct/Outcome	Questions
How an individual feels about conducting/taking part in an intervention	What were your thoughts about the *PCEssentials* study?<br>• *When you first heard about it*<br>• *While you were recruiting*
The extent to which the participant understands the intervention, and how the intervention works	What is your understanding about how *PCEssentials* works?
The participant's confidence that they can perform the behaviour(s) required to deliver/take part in the intervention	How confident were you that you could do what you needed to deliver/take part in *PCEssentials*?<br>• *Recruitment*<br>• *Conducting the intervention sessions*<br>• *Identifying triggers for care escalation/managing deterioration*
The perceived amount of effort that is required to deliver/take part in the intervention	How burdensome is *PCEssentials* to deliver/take part in compared to usual care?<br>• *In what way is it less/more burdensome?*
The extent to which benefits, profits, or values must be given up to deliver/take part in an intervention	As a clinician do you feel you had to give anything up to deliver/take part in *PCEssentials*?<br>• *Can you give some examples?*<br>Do you think the quality of care delivered in *PCEssentials* differs from usual care?<br>• *Can you give some examples?*<br>From a cost perspective to your service, are there any advantages/disadvantages to the *PCEssentials* model compared to usual care?
The extent to which the intervention has good fit with an individual's value system	Does *PCEssentials* meet your needs as a clinician?<br>• *Why or why not?*<br>• *What could be changed to meet your needs?*<br>• *What was it that helped meet your needs?*
The extent to which the intervention is perceived as likely to achieve its purpose	Looking back at the program since it started, how effective do you think *PCEssentials* is overall?<br>• *In what way is it effective/not effective?*<br>• *Clinician perspective*<br>• *Patient needs*

For peer review only

	Can you give me an example of something you really liked/disliked about *PCEssentials?*<br>How could *PCEssentials* be improved?<br>Is there anything else you wanted to say about *PCEssentials*?

1. Proctor E, Silmere H, Raghavan R, Hovmand P, Aarons G, Bunger A, et al. Outcomes for implementation research: conceptual distinctions, measurement challenges, and research agenda. Administration and policy in mental health. 2011;38(2):65-76.

For peer review only

SPIRIT
STANDARD PROTOCOL ITEMS: RECOMMENDATIONS FOR INTERVENTIONAL TRIALS

SPIRIT 2013 Checklist: Recommended items to address in a clinical trial protocol and related documents*

Section/item	Item No	Description	Y/N
**Administrative information**			
Title	1	Descriptive title identifying the study design, population, interventions, and, if applicable, trial acronym	Y
Trial registration	2a	Trial identifier and registry name. If not yet registered, name of intended registry	Y
	2b	All items from the World Health Organization Trial Registration Data Set	Y
Protocol version	3	Date and version identifier	Y
Funding	4	Sources and types of financial, material, and other support	Y
Roles and responsibilities	5a	Names, affiliations, and roles of protocol contributors	Y
	5b	Name and contact information for the trial sponsor	Y
	5c	Role of study sponsor and funders, if any, in study design; collection, management, analysis, and interpretation of data; writing of the report; and the decision to submit the report for publication, including whether they will have ultimate authority over any of these activities	Y
	5d	Composition, roles, and responsibilities of the coordinating centre, steering committee, endpoint adjudication committee, data management team, and other individuals or groups overseeing the trial, if applicable (see Item 21a for data monitoring committee)	Y
**Introduction**			
Background and rationale	6a	Description of research question and justification for undertaking the trial, including summary of relevant studies (published and unpublished) examining benefits and harms for each intervention	Y
	6b	Explanation for choice of comparators	Y
Objectives	7	Specific objectives or hypotheses	Y

For peer review only

Trial design	8	Description of trial design including type of trial (eg, parallel group, crossover, factorial, single group), allocation ratio, and framework (eg, superiority, equivalence, noninferiority, exploratory)	Y

Methods: Participants, interventions, and outcomes

Study setting	9	Description of study settings (eg, community clinic, academic hospital) and list of countries where data will be collected. Reference to where list of study sites can be obtained	Y
Eligibility criteria	10	Inclusion and exclusion criteria for participants. If applicable, eligibility criteria for study centres and individuals who will perform the interventions (eg, surgeons, psychotherapists)	Y
Interventions	11a	Interventions for each group with sufficient detail to allow replication, including how and when they will be administered	Y
	11b	Criteria for discontinuing or modifying allocated interventions for a given trial participant (eg, drug dose change in response to harms, participant request, or improving/worsening disease)	Y
	11c	Strategies to improve adherence to intervention protocols, and any procedures for monitoring adherence (eg, drug tablet return, laboratory tests)	Y
	11d	Relevant concomitant care and interventions that are permitted or prohibited during the trial	Y
Outcomes	12	Primary, secondary, and other outcomes, including the specific measurement variable (eg, systolic blood pressure), analysis metric (eg, change from baseline, final value, time to event), method of aggregation (eg, median, proportion), and time point for each outcome. Explanation of the clinical relevance of chosen efficacy and harm outcomes is strongly recommended	Y
Participant timeline	13	Time schedule of enrolment, interventions (including any run-ins and washouts), assessments, and visits for participants. A schematic diagram is highly recommended (see Figure)	Y
Sample size	14	Estimated number of participants needed to achieve study objectives and how it was determined, including clinical and statistical assumptions supporting any sample size calculations	Y
Recruitment	15	Strategies for achieving adequate participant enrolment to reach target sample size	Y

Methods: Assignment of interventions (for controlled trials)

Allocation:

Sequence generation	16a	Method of generating the allocation sequence (eg, computer-generated random numbers), and list of any factors for stratification. To reduce predictability of a random sequence, details of any planned restriction (eg, blocking) should be provided in a separate document that is unavailable to those who enrol participants or assign interventions	Y
Allocation concealment mechanism	16b	Mechanism of implementing the allocation sequence (eg, central telephone; sequentially numbered, opaque, sealed envelopes), describing any steps to conceal the sequence until interventions are assigned	Y
Implementation	16c	Who will generate the allocation sequence, who will enrol participants, and who will assign participants to interventions	Y
Blinding (masking)	17a	Who will be blinded after assignment to interventions (eg, trial participants, care providers, outcome assessors, data analysts), and how	Y
	17b	If blinded, circumstances under which unblinding is permissible, and procedure for revealing a participant's allocated intervention during the trial	N/A
**Methods: Data collection, management, and analysis**			
Data collection methods	18a	Plans for assessment and collection of outcome, baseline, and other trial data, including any related processes to promote data quality (eg, duplicate measurements, training of assessors) and a description of study instruments (eg, questionnaires, laboratory tests) along with their reliability and validity, if known. Reference to where data collection forms can be found, if not in the protocol	Y
	18b	Plans to promote participant retention and complete follow-up, including list of any outcome data to be collected for participants who discontinue or deviate from intervention protocols	Y
Data management	19	Plans for data entry, coding, security, and storage, including any related processes to promote data quality (eg, double data entry; range checks for data values). Reference to where details of data management procedures can be found, if not in the protocol	Y
Statistical methods	20a	Statistical methods for analysing primary and secondary outcomes. Reference to where other details of the statistical analysis plan can be found, if not in the protocol	Y
	20b	Methods for any additional analyses (eg, subgroup and adjusted analyses)	Y
	20c	Definition of analysis population relating to protocol non-adherence (eg, as randomised analysis), and any statistical methods to handle missing data (eg, multiple imputation)	Y

For peer review only - http://bmjopen.bmj.com/site/about/guidelines.xhtml

Methods: Monitoring

Data monitoring	21a	Composition of data monitoring committee (DMC); summary of its role and reporting structure; statement of whether it is independent from the sponsor and competing interests; and reference to where further details about its charter can be found, if not in the protocol. Alternatively, an explanation of why a DMC is not needed	Y
	21b	Description of any interim analyses and stopping guidelines, including who will have access to these interim results and make the final decision to terminate the trial	N/A
Harms	22	Plans for collecting, assessing, reporting, and managing solicited and spontaneously reported adverse events and other unintended effects of trial interventions or trial conduct	Y
Auditing	23	Frequency and procedures for auditing trial conduct, if any, and whether the process will be independent from investigators and the sponsor	Y

Ethics and dissemination

Research ethics approval	24	Plans for seeking research ethics committee/institutional review board (REC/IRB) approval	Y
Protocol amendments	25	Plans for communicating important protocol modifications (eg, changes to eligibility criteria, outcomes, analyses) to relevant parties (eg, investigators, REC/IRBs, trial participants, trial registries, journals, regulators)	Y
Consent or assent	26a	Who will obtain informed consent or assent from potential trial participants or authorised surrogates, and how (see Item 32)	Y
	26b	Additional consent provisions for collection and use of participant data and biological specimens in ancillary studies, if applicable	N/A
Confidentiality	27	How personal information about potential and enrolled participants will be collected, shared, and maintained in order to protect confidentiality before, during, and after the trial	Y
Declaration of interests	28	Financial and other competing interests for principal investigators for the overall trial and each study site	Y
Access to data	29	Statement of who will have access to the final trial dataset, and disclosure of contractual agreements that limit such access for investigators	Y
Ancillary and post-trial care	30	Provisions, if any, for ancillary and post-trial care, and for compensation to those who suffer harm from trial participation	Y

For peer review only - http://bmjopen.bmj.com/site/about/guidelines.xhtml

Dissemination policy	31a	Plans for investigators and sponsor to communicate trial results to participants, healthcare professionals, the public, and other relevant groups (eg, via publication, reporting in results databases, or other data sharing arrangements), including any publication restrictions	Y
	31b	Authorship eligibility guidelines and any intended use of professional writers	N/A
	31c	Plans, if any, for granting public access to the full protocol, participant-level dataset, and statistical code	Y
**Appendices**			
Informed consent materials	32	Model consent form and other related documentation given to participants and authorised surrogates	N/A
Biological specimens	33	Plans for collection, laboratory evaluation, and storage of biological specimens for genetic or molecular analysis in the current trial and for future use in ancillary studies, if applicable	N/A

*It is strongly recommended that this checklist be read in conjunction with the SPIRIT 2013 Explanation & Elaboration for important clarification on the items. Amendments to the protocol should be tracked and dated. The SPIRIT checklist is copyrighted by the SPIRIT Group under the Creative Commons "Attribution-NonCommercial-NoDerivs 3.0 Unported" license.

For peer review only

BMJ Open

Prostate Cancer Survivorship Essentials for men with prostate cancer on androgen deprivation therapy: Protocol for a randomised controlled trial of a tele-based nurse-led survivorship care intervention (PCEssentials Hormone Therapy Study)

Journal:	*BMJ Open*
Manuscript ID	bmjopen-2024-084412.R1
Article Type:	Protocol
Date Submitted by the Author:	05-Feb-2024

Complete List of Authors:	Green, Anna; University of Southern Queensland, Centre for Health Research<br>Newton, Robert; Edith Cowan University, Exercise Medicine Research Institute<br>Smith, David; The University of Sydney, The Daffodil Centre, a joint venture with Cancer Council NSW<br>Tuffaha, Haitham; The University of Queensland<br>Galvão, Daniel; Edith Cowan University, Exercise Medicine Research Institute<br>Heathcote, Peter; Brisbane Urology Clinic<br>Patel, Manish; University of Sydney<br>Christie, David; Genesiscare; Bond University<br>Egger, Sam; The University of Sydney, The Daffodil Centre, a joint venture with Cancer Council NSW<br>Sara, Sally; Prostate Cancer Foundation of Australia, Nursing Programs<br>Heneka, Nicole; University of Southern Queensland, Centre for Health Research<br>Chambers, Suzanne K; Australian Catholic University<br>Dunn, Jeff; University of Southern Queensland, Centre for Health Research; Prostate Cancer Foundation of Australia

**Primary Subject Heading**:	Oncology
Secondary Subject Heading:	Health economics, Mental health, Nursing, Patient-centred medicine
Keywords:	Prostatic Neoplasms, Randomized Controlled Trial, Health Education, Nursing Care, Quality of Life

SCHOLARONE™
Manuscripts

Title: Prostate Cancer Survivorship Essentials for men with prostate cancer on androgen deprivation therapy: Protocol for a randomised controlled trial of a tele-based nurse-led survivorship care intervention (PCEssentials Hormone Therapy Study)

ABSTRACT

Introduction

Androgen deprivation therapy (ADT) is commonly used to treat men with locally advanced or metastatic prostate cancer. Men receiving ADT experience numerous side effects and frequently report unmet supportive care needs. An essential part of quality cancer care is survivorship care. To date an optimal effective approach to survivorship care for men with prostate cancer on ADT has not been described. This protocol describes a randomised trial of tele-based nurse-led survivorship that addresses this knowledge gap:

1. Determine the effectiveness of a nurse-led survivorship care intervention (*PCEssentials*), relative to usual care, for improving health-related quality of life (HR-QoL) in men with prostate cancer undergoing ADT.
2. Evaluate *PCEssentials* implementation strategies and outcomes, including cost-effectiveness, compared to usual care.

Methods and analysis

This is an effectiveness-implementation hybrid (Type 1) trial with participants randomised to one of two arms: i) minimally enhanced usual care; and ii) nurse-led Prostate Cancer Survivorship Essentials (*PCEssentials*) delivered over four tele-based sessions, with a booster session five months after session one. Eligible participants are Australian men with prostate cancer commencing ADT and expected to be on ADT for a minimum of 12 months. Participants are followed-up at 3-, 6-, and 12-months post-recruitment. Primary outcomes are HR-QoL and self-efficacy. Secondary outcomes are psychological distress, insomnia, fatigue, and physical activity. A concurrent process evaluation with participants and study stakeholders will be undertaken to determine effectiveness of delivery of *PCEssentials*.

Ethics and dissemination

Ethics approval was obtained from the Metro South Health HREC (HREC/2021/QMS/79429). All participants are required to provide written informed consent. Outcomes of this trial will be published in peer-reviewed journals. The findings will be presented at conferences and meetings, local hospital departments, participating organisations/clinical services, and university seminars, and communicated at community and consumer-led forums.

Clinical Trial Registration: Australian New Zealand Clinical Trials Registry (ANZCTR) ACTRN12622000025730

Protocol version: 1.6

Protocol date: 16.08.2023

Funding: This work is supported by the National Health and Medical Research Council (NH&MRC) grant number APP2006528.

Word count: 4533

Key words: prostatic neoplasms; randomized controlled trial; health education; nursing care; quality of life

For peer review only - http://bmjopen.bmj.com/site/about/guidelines.xhtml

ARTICLE SUMMARY

Strengths and limitations of this study

- The effectiveness-implementation design allows for a concurrent process evaluation which will provide immediate implementation data.
- A cost-utility analysis will provide important economic evaluation data.
- Tele-based interventions are highly acceptable to men with prostate cancer and applicable to geographically dispersed and vulnerable populations.

  The pragmatic decision to exclude non-English speaking patients from the trial may influence the generalisability of study findings to patients from linguistically diverse backgrounds.

For peer review only

INTRODUCTION

Prostate cancer (PCa) is the most common cancer diagnosed in Australia (1). While men are living longer following diagnosis, longitudinal research has characterised a subgroup of 35%-40% of men who experience long-term decrements in health-related quality of life (HR-QoL) (2). In particular, men who are on androgen deprivation therapy (ADT) experience consistently poorer physical and mental HR-QoL over the long term (2-6).

While ADT is effective in treating PCa and increasing survival, it is associated with multiple, often debilitating side effects, which manifest as changes in physical, cognitive, social, and sexual functioning (3, 7-9). Iatrogenic effects may include mood disturbances, increased fat mass, body feminisation, cognitive decline, functional impairment, frailty, fatigue, and sexual dysfunction (3, 4, 6-10). ADT also increases the risk of developing new co-morbidities, including cardiovascular conditions, diabetes, sarcopenia, and osteoporosis (11). Compared with men receiving other treatments, those undergoing ADT report poorer HR-QoL and higher levels of psychological distress, including depression, anxiety, relationship changes, cognitive and affective symptoms, and sleep disturbances (3, 4, 6-9, 12). The prevalence of psychological distress in PCa survivors is reported to be between 11%-27% (13), and regardless of other treatments, receiving ADT is predictive of higher distress (12). Further, men undergoing ADT have an increased risk of suicide compared to those who do not, particularly in older men and in the first six-months post diagnosis (14). Unmet supportive care needs are highly prevalent in these men, with unmet physical, psychological, sexual, existential, and informational (12, 15) needs that persist at 15-years post-diagnosis (16). Over one-third (37%) of men with PCa will report at least one long-term unmet supportive care need particularly at the start of treatment when side effects are new or unknown and HR-QoL is first impacted (16). This is of particular concern for men receiving ADT who report feeling unprepared to manage substantial treatment side effects that impact on quality of life (17). Further, despite routine clinical follow-up, men receiving ADT rarely receive tailored person-centred interventions in a timely manner, adversely impacting HR-QoL with poor management of side effects and self-efficacy (12, 15). Men treated with ADT are a vulnerable high-need patient group for whom evidence-based survivorship care is crucial.

Preliminary research on survivorship care for men with PCa

Previous prostate cancer survivorship guidelines published by the American Cancer Society a decade ago (18) were limited by an over reliance on expert opinion and lack of a robust evidence-base (19). Existing survivorship guidelines have also been limited by lack of consumer involvement (20, 21). Our group has contextualised survivorship care for PCa (20, 22, 23) and produced a contemporary survivorship care framework for men with prostate cancer. The resulting *Survivorship Essentials Framework* (Figure 1) proposes holistic survivorship care for men with PCa and was developed by a uniquely inclusive expert clinical and community group (23). The framework has been widely endorsed by key PCa and urological groups in Australia and New Zealand. Based on our survivorship framework, we have developed a new model of care, Prostate Cancer Survivorship Essentials (*PCEssentials*), which integrates evidence-based strategies to improve men's quality of life outcomes after ADT in a men-centred approach, where personal agency intersects with all aspects of care.

We propose an Australian effectiveness-implementation hybrid (Type 1) randomised trial (24) of tele-based nurse-led survivorship care with 236 PCa survivors undergoing ADT. This is the

For peer review only

first such study internationally to address this problem. The proposed study will have two arms: i) minimally enhanced usual care; and ii) nurse-led Prostate Cancer Survivorship Essentials (*PCEssentials*) delivered over four tele-based sessions, with a subsequent booster session five months after the first session. In accordance with a Type 1 hybrid trial, a concurrent process evaluation, guided by the Conceptual Framework for Implementation Outcomes (25), will be undertaken to determine effectiveness of the *PCEssentials* intervention delivery, and the potential for implementation of the intervention at scale.

Aims

Aim 1: Determine the effectiveness of a nurse-led survivorship care intervention (*PCEssentials*), relative to usual care, for improving HR-QoL in men with PCa undergoing ADT.

Aim 2: Evaluate *PCEssentials* implementation strategies and outcomes, including cost-effectiveness of *PCEssentials*, with respect to usual care, as well as acceptability, adoption, appropriateness, feasibility, fidelity, penetration, and sustainability.

Primary hypothesis

We hypothesise that *PCEssentials* will be more cost-effective than usual care. Furthermore, relative to men receiving usual care at 3-, 6-, and 12-months after recruitment, men who receive *PCEssentials* will have: i) higher HR-QoL; ii) increased self-efficacy; iii) less psychological distress; and iv) improved sleep and lower fatigue.

METHODS AND ANALYSIS

Study design

A Type 1 effectiveness-implementation hybrid randomised trial (24) of a nurse-led survivorship care intervention (*PCEssentials*), relative to usual care, for improving HR-QoL in men with PCa undergoing ADT. A concurrent process evaluation will determine the effectiveness of intervention delivery, and the potential for implementation at scale. The study design has been guided by the CONSORT criteria (26).

There are four key study time-points:

- T1 – Baseline: prior to randomisation
- T2 – 3 months post-recruitment
- T3 – 6 months post-recruitment
- T4 – 12 months post-recruitment

This study will be undertaken in accordance with the National Statement on Ethical Conduct in Human Research (2007 – updated 2018) (27) and the Australian Code for the Responsible Conduct of Research (2018) (28). The study commenced in January 2022 upon receiving ethics approval, with a planned end date of August 2026.

Research population

There are two research populations for this study:

For peer review only

1. Patient participants (n=236): Australian men (aged 18 years or over) diagnosed with PCa commencing, or within 3 months of having commenced, ADT.

2. Process evaluation participants (n=148): Study stakeholders (n=30) who are directly involved in study delivery and/or translation into clinical practice, including participating service managers, recruiting clinicians, nurses delivering the intervention, health professionals; and patient participants in the intervention group (n=118). While all participants in the intervention group will complete program acceptability assessments at two study time-points (T1 and T3), approximately 20 of these patient participants will be purposively selected/invited to take part in a semi-structured interview (T3) to explore their experiences of the intervention. Purposive sampling will ensure a patient subgroup with maximum diversity (e.g., based on age, background, location, partnered or un-partnered). We anticipate reaching data saturation for the process evaluation with this number of participants.

Inclusion criteria

Men recruited to the study will: i) have been diagnosed with PCa and be commencing, or within 3 months of having commenced ADT, and expected (based on clinical information) to be on ADT for a minimum continuous period of 12 months; ii) are able to read and speak English; iii) are able to give written informed consent; iv) have no previous history of head injury, dementia, or psychiatric illness; v) have no other concurrent cancer; and vi) have mobile and/or landline phone access.

Exclusion criteria

Men with castrate resistant and confirmed metastatic disease are excluded on the basis of having progressive and incurable disease that may rapidly progress and the study doesn't meet their needs.

Research project setting/location

There are multiple recruitment settings through clinicians in major treatment centres across Australia and by patient self-referral. Study information for patient self-referral is disseminated through investigator networks.

Research project procedures

1. Intervention

Following referral (clinician or self) to the study team, research staff screen potential participants for eligibility and conduct an informed consent process (Figure 2). Once eligibility is confirmed, and written informed consent received, participants receive the baseline assessments (T1) via mail. Upon return of T1 assessments, the study team randomises participants into the intervention or minimally enhanced usual care ('usual care') group.

Men randomised to the intervention group commence the *PCEssentials* intervention, a five-session psychoeducation program delivered by trained Prostate Cancer Specialist Nurses via

For peer review only

mobile and/or landline telephone. This includes four sessions over three months and a booster session at five months after the first session. Men in the intervention group are also be offered a home-based exercise program and encouraged to seek at least one planning session with an Accredited Exercise Physiologist (AEP).

Men in the usual care group receive their standard management, minimally enhanced with a package of evidence-based resources.

Men in both groups will continue to attend their standard PCa related care, and complete study assessments at 3-, 6-, and 12-months post recruitment.

2. Process evaluation

A mixed methods approach will examine the elements of the Conceptual Framework for Implementation Outcomes (25) as they relate to the *PCEssentials* intervention, namely: acceptability, adoption, appropriateness, penetration, feasibility, fidelity, and sustainability. To assess program acceptability and feasibility, clinical stakeholders involved in the delivery or oversight of the program will be invited by the partner investigator at each site to participate in: i) a short online survey when recruitment commences and ends at the site; and ii) a semi-structured interview when recruitment ends. Invitations will be sent to eligible clinical stakeholders via email, with written informed consent sought prior to surveys/interviews being undertaken.

Recruitment

Recruitment is undertaken through clinicians in major treatment centres across Australia. With patient permission, clinicians are asked to directly refer eligible patients to the study team who then proceed with an informed consent process. A two-phase consent process is used for patient participants who are referred by a clinician: i) written, or verbal, where appropriate, permission to provide the patient's contact details to the study team for follow-up; and ii) written informed consent to take part in the study.

Additionally, men may self-refer having identified the study through media promotion and PCa support groups. In this case, potential participants contact the research team directly and provide written informed consent after being screened for eligibility.

Based on our experience with previous interventions in similar cohorts (29-31), and active participation of our project partners, we anticipate a recruitment period of 18 months to randomise 236 patients.

Randomisation

Randomisation to study group condition occurs following receipt of baseline assessments (Figure 2). Randomisation occurs in varying block sizes of four, six and eight (to ensure an unpredictable allocation sequence with equal numbers of men in each treatment group at the completion of each block) with no stratification factors. The randomisation sequence is undertaken by the project manager and concealed from investigators. Project staff tracking assessments (data analysts) will be blinded to condition.

For peer review only

Research project process

1. Patients

Patient-reported outcomes and experience assessments are completed at each study time-point (T1-T4). Following informed consent, participants are sent the T1 assessments for completion. Upon receipt of completed T1 assessments by the research team, participants are randomised into either i) minimally enhanced usual care (control); or ii) nurse-led survivorship care: *PCEssentials* (intervention group).

i) Minimally enhanced usual care
Standard management, minimally enhanced with evidence-based patient education materials about the use of ADT to treat PCa, and information about free telephone-based cancer information and support services in the participant's home state.

ii) Nurse-led survivorship care (*PCEssentials*)
The nurse-led intervention is telephone delivered over five sessions by trained Prostate Cancer Specialist Nurses, guided by manualised intervention protocols, and supervised by an experienced prostate cancer specialist nurse and a health psychologist with extensive experience in prostate cancer supportive care. The intervention includes five modules covering: psycho-education with tailored distress management strategies; decision support; treatment education with self-management and skills training for symptom effects, including exercise/physical activity resources and support; and communicating with health professionals including a referral pathway to their general practitioner for chronic disease management.

A problem solving approach that supports personal agency underpins each component (20), with the first four sessions to be delivered by telephone over three months, and an additional booster session five months after the initial session module has been completed. A problem-solving approach (32) that is responsive to masculine models of coping and life stage was chosen as the underlying mechanism of support to enhance personal agency.

Men with PCa experience improved psychological outcomes when they engage in approach coping that addresses the threats associated with their cancer (33), and active problem solving is consistent with male values around strength, self-reliance and action (34). Problem-solving therapy (PST) has been found to be effective in reducing depression and disability in older people ($\geq$60 years of age) with chronic illness (32). Our intervention targets include major challenges identified by men (e.g., psychological distress, disease and treatment effects, communicating with health professionals) and applies PST to enhance men's personal agency in defining and formulating the nature of their specific problems, generating potential solutions, systematically evaluating possible consequences of solutions and selecting an appropriate solution, and monitoring solution outcomes. A self-help survivorship resource that addresses key PCa-related challenges with evidence-based coping strategies is provided and this connects directly to the nurse-led intervention session content (35).

Distress screening and problem identification occurs at each session using the Distress Thermometer and is integrated with distress and symptom management strategies (36). The

For peer review only - http://bmjopen.bmj.com/site/about/guidelines.xhtml

booster session checks participant progress, reinforces self-management skills, and troubleshoots concerns that may have persisted.

A home-based physical exercise program is offered, where men are encouraged to seek at least one planning session with an Accredited Exercise Physiologist (AEP) within their treatment team, accessed by telephone or internet. The nurse specialist encourages exercise maintenance, including aerobic and resistance training as per the Australian Exercise Medicine for Cancer guidelines with referral to an AEP, if required (37).

Men have identified that the Prostate Cancer Specialist Nurse/clinical nurse is highly acceptable as the provider of survivorship care, an approach described as the most efficient in terms of use and resources and being suitable for most care settings (38). Tele-based interventions are also highly acceptable to men with PCa (85% consent rate (22)), are accessible for patients who are very unwell (39), have been shown to be an effective delivery method for problem solving therapy (32), and in advanced disease show low attrition rates compared to face-to-face delivery (20). This delivery method is also applicable to geographically dispersed and vulnerable populations with high potential for population-based translation.

2. Process evaluation

Process assessments are collected via: i) surveys using the Program Acceptability: Acceptability of Intervention Measure (AIM), Intervention Appropriateness Measure (IAM) and Feasibility of Intervention Measure (FIM) (40) at T1 and T3 (patient participants) and when recruitment commences and ends (other stakeholders), as well as the Working Alliance Inventory – Short Revised (WAI-SR) scale (41) at T3 (intervention group patient participants only); ii) semi-structured interviews with stakeholders at T3 (patient participants) and when recruitment ends (other stakeholders); and iii) intervention fidelity and adherence assessments at multiple study time-points, to identify barriers and facilitators to implementation, and determine if high intervention fidelity is achieved.

Research outcomes and measurement tools

Previously validated and reliable patient-reported outcome assessments are administered by mail to men at four-time points: baseline/recruitment (T1), 3 months (T2), 6 months (T3), and 12 months (T4) after recruitment. Primary outcomes are HR-QoL and self-efficacy. Secondary outcomes include global psychological distress, insomnia, fatigue, and life satisfaction. Demographic moderators/disease variables (e.g., cancer grade, stage, time since diagnosis, time since treatment) and a health service use diary are self-reported. Assessments are self-report pen and paper.

Primary outcomes

Health-Related Quality of Life: The *Functional Assessment of Cancer Therapy – Prostate (FACT-P)* (42) assesses men's disease-specific quality of life across five domains: physical, social/family, emotional, functional well-being, and PCa specific concerns (42). The *Assessment of Quality of Life* (AQoL-8D) instrument is used to derive health utility scores and general HR-QoL among patients. This tool has increased measurement sensitivity to

psychosocial elements of health compared to other instruments, since it comprises five psychosocial dimensions (mental health, happiness, coping, relationships, and self-worth) and three physical dimensions (independent living, pain, and senses) (43). The physical function subscale from the *Medical Outcomes Study Short-Form-36* (SF-36) questionnaire will be used as an indicator of patient-related physical functioning QoL (44). We recently reported improvements in physical function in PCa patients with advanced disease and bone metastases following an exercise intervention using this measure, and in those on ADT with localised disease (45).

Self-efficacy: The 11-item *Cancer Survivorship Self-Efficacy Scale* (CS-SES) (46) assesses self-efficacy to manage problems arising from cancer and its treatment specifically.

Secondary outcomes

Psychological distress: The Generalized Anxiety Disorder (GAD-7) scale (47) and the depression subscale of the Patient Health Questionnaire (PHQ-9) (48) will measure psychological distress. The seven item GAD-7 scale screens for, and assesses the severity of, generalised anxiety disorder in clinical practice and research. The nine item PHQ-9 scale screens for, and assesses the severity of, depression and includes a specific item on suicidal ideation.

Insomnia: The *Insomnia Severity Index* (ISI) is the worldwide standard, seven-item self-report measure to evaluate: (a) severity of sleep-onset, (b) sleep maintenance, (c) early morning awakening problems, (d) satisfaction with current sleep pattern, (e) interference with daily functioning, (f) noticeability of impairment attributed to the sleep problem, and (g) level of distress caused by the sleep problem (49).

Fatigue: The *Multidimensional Fatigue Symptom Inventory-Short Form* (MFSI-SF) (50) assesses general fatigue, physical fatigue, emotional fatigue, mental fatigue, and vigour.

Physical activity/exercise: Godin-Shephard Leisure-Time Physical Activity Questionnaire (GSLTPAQ) (51), modified to include questions on resistance training, reflecting current best practice in exercise intervention trials for men with PCa (52), will assess physical activity.

Process evaluation

Program acceptability: The Acceptability of Intervention Measure (AIM), Intervention Appropriateness Measure (IAM) and Feasibility of Intervention Measure (FIM) (33) is a short self-reported assessment that is collected at T1 and T3 (patient participants) to determine patients' experiences of the study from recruitment to six months post recruitment. For patient participants, this is included in the self-reported study assessments mailed to them at T1 and T3. The therapeutic alliance between patients in the intervention group and the nurses delivering the intervention will also be assessed by the 12 item Working Alliance Inventory – Short Revised (WAI-SR) (41). This will be included in the self-reported study assessments mailed to patient participants at T3.

All other study stakeholders receive the same assessments as an online survey when recruitment starts and ends to determine their study experience.

For peer review only

Interviews: Semi-structured interviews exploring the constructs of the Conceptual Framework for Implementation Outcomes (25) will be undertaken to determine effectiveness of the *PCEssentials* intervention delivery, and the potential for implementation of the intervention at scale. The interview question route informed by the literature is included in Supplementary File 1.

Statistical considerations and data analysis

Recent meta-analyses conclude that individually focussed psychological interventions should produce improvements in psychological distress of at least a medium effect size (d=0.40) that will be clinically meaningful (53). To see an effect of this size or greater in our primary outcome, psychological distress at 12 months, with 80% power and alpha=0.05, we will require 99 participants in each group to complete the intervention. Assuming 15% attrition, we will recruit 236 patients to the study (118 patients per group).

1. Intervention effectiveness

The study is a two-arm randomised controlled trial with repeated assessments across time and with continuous primary outcome variables. Recruitment bias will be assessed by comparing sociodemographic and clinical variables for consenters with non-consenters using t-tests (or Mann-Whitney U tests) for continuous variables and chi-square tests for categorical variables. Possible differential attrition will be assessed by comparing baseline characteristics of drop-outs and continuing participants using t-tests (or Mann-Whitney U tests if appropriate) for continuous variables and chi-square tests for categorical variables. Intention-to-treat analyses will be conducted. Between-group mean differences in change from baseline outcome scores at 3, 6 and 12 months will be analysed by fitting mixed effects regression models. Intervention (intervention/usual care) will be included as the main effect. Indicators for participants will be included as a random effect to account for the non-independence of repeated observations from the same individual. Sensitivity analysis will assess the effects of attrition. Mixed effects models with maximum likelihood estimation minimise bias that may arise from ignoring missing observations, and use all available data, thereby maximising statistical power to detect effects. The mean and 95% confidence interval will be calculated for satisfaction with the intervention. Missing data will be examined for patterns of missingness and addressed with the appropriate multiple imputation methods, if required. The investigator team includes a dedicated biostatistician who will undertake analyses.

2. Process evaluation

Process evaluation assessments will be analysed using a combination of descriptive statistics (measures of program acceptability), and deductive directed content analysis (semi-structured interviews) (54). Joint display tables will facilitate the data integration process and facilitate the drawing of inferences from the integrated data (55).

3. Cost-utility analysis

For peer review only - http://bmjopen.bmj.com/site/about/guidelines.xhtml

A cost-utility analysis of the intervention relative to minimally enhanced usual care from both healthcare payer and societal perspectives will be conducted alongside the *PCEssentials* trial. Costs will be obtained by identifying, measuring and valuing the health resources used. At baseline, participants are given a health service use diary to record direct health resources utilised (e.g., GP visits, treatments, and hospitalisations), as well as out-of-pocket expenses and indirect costs (e.g., productivity loss). The diaries will also be collected during the T2, T3 and T4 assessments. Healthcare resources will be valued using unit prices from standard costing resources such as the Medicare Benefits Schedule and relevant Australian award wages. Quality adjusted life years (QALY) gained will be estimated, which is a measure of a patient's life expectancy, weighted by his health-related quality of life (i.e., utility score) measured using the AQoL-8D at baseline, 3, 6 and 12 months. A multivariate generalised linear model will be used to adjust for differences in baseline AQoL-8D scores, demographics and disease classifications. The incremental cost-effectiveness ratio (ICER) will be calculated, which is the difference in mean costs divided by the difference in mean QALYs. Non-parametric bootstrapping will be used to characterise uncertainty around the ICER. If the intervention appears to be cost-effective, we will calculate the expected value of implementation, which is the net monetary benefit of the intervention (i.e., monetary benefits – costs) multiplied by the population of PCa patients expected to benefit from the intervention and adjusted by various patients' adherence and clinicians' uptake rates. Uptake rates will be obtained from a formal elicitation exercise and will inform a Bass model to forecast diffusion (i.e., implementation over time) (56).

Patient and public involvement statement

This research project was developed through a collaboration between the University of Southern Queensland and the Prostate Cancer Foundation of Australia as the co-lead organisations. The Prostate Cancer Foundation of Australia is a broad-based community organisation and the peak national body for PCa in Australia. Patient/public involvement in the research has been carried through the conceptualisation and design of the study and *PCEssentials* intervention, to recruitment and delivery of the intervention through this partnership. Consumer and clinical representatives have contributed to project steering committees and development of the intervention. The Prostate Cancer Foundation of Australia will assist with dissemination of study results through their consumer and clinical stakeholder network ensuring future patient/public engagement.

ETHICS AND DISSEMINATION

Ethics approval for this study was obtained from the Metro South Health Human Research Ethics Committee (HREC/2021/QMS/79429).

Safety considerations

Experienced Prostate Cancer Specialist Nurses ('intervention nurses') are responsible for the delivery of the intervention. Intervention nurses receive: i) additional training in the study-specific protocol and *PCEssentials* intervention; ii) an intervention manual detailing session content and activities; and iii) weekly supervision and debriefing by study investigators with extensive experience in the delivery of the prostate cancer supportive care. All other study staff will also receive protocol specific and research processes training.

For peer review only

Data management and monitoring

Written, informed consent is obtained from each patient and clinical stakeholder prior to study enrolment and any study activities being undertaken (Supplementary File 2 and Supplementary File 3). Patient participants are given a unique participant identification code (ID). This ensures that all identifying data can be removed before data analysis commences. This project ID enables the research team to manage the data in a confidential manner. The master list linking identifying participant information and ID number is maintained in a locked cabinet, separate from the participant database at the Prostate Cancer Foundation of Australia. All data collected for each participant is kept in a participant file (identified by ID number only) which contains the Case Report Forms, any corrected and amended data, copies of adverse event reports, file notes etc. All study files are stored in accordance with Good Clinical Practice guidelines.

Form tracking is via participant ID number only. The participant database is stored on a password-protected hard drive maintained by the study investigators. Data will be analysed by ID number only. All information presented in dissemination will be de-identified group data that will not allow the identification of individual participants.

Treatment fidelity

The intervention is manualised and intervention nurses complete a checklist of components delivered at each session. Throughout the study, sessions are audiotaped and 15% of sessions will be reviewed to assess adherence to protocol. The intervention nurses are supervised by an investigator who is a qualified psychologist with oversight on treatment fidelity monitoring according to NIH guidelines (57).

Ethical considerations

There are two potential risks for participants related to the intervention: (i) minor psychological distress may be experienced by some participants while discussing issues relating to treatment, side-effects, and psychosocial impact during the intervention; (ii) side-effects arising from changes in physical activity (such as muscle soreness) if participants choose to take part in the exercise component of the intervention. However, the psychological distress that may be experienced by some participants will be no greater than that experienced when discussing issues related to PCa management with their doctor. Similarly, the side-effects that may be experienced by some participants while in the process of the exercise component are likely to be no greater than the risks of day-to-day living as people can undertake changes in their level of physical activity.

Adverse events will be recorded by the research team immediately upon their notification. Should any adverse or serious adverse events occur, the research team will report to the governing ethics committee, review relevant risk assessments, aim to mitigate future risk of adverse events and provide the appropriate duty of care to the participant/s concerned.

Risk mitigation

For peer review only

Psychological distress will be minimised by identifying those individuals who are experiencing high distress and tailoring the intervention to specifically manage stress in these individuals. The intervention specialist nurses are trained to assess psychological distress and to manage this during the nurse-led intervention. Participants who request additional psychological support beyond the intervention will be referred to additional sources including the Prostate Cancer Foundation of Australia Telenursing Service (direct referral to the telenursing service manager who is not an intervention nurse), Beyond Blue, Lifeline and/or other relevant local services. Medical management of participants will be managed as per their usual care.

Dissemination

Outcomes of this trial will be published in peer-reviewed journals, and the findings presented at national and international conferences and meetings. Findings will also be communicated at community and consumer-led forums and presented at local hospital departments, participating organisations/clinical services, and university seminars. This study is designed so that outputs are translatable into practice to improve the health and well-being of men with PCa receiving ADT. Should it prove effective, our intervention may be utilised in a range of settings, including broad-reach tele-based support programs; and through support services across Australia that are conducted by state Cancer Councils and the Prostate Cancer Foundation of Australia, as well as through similar support service infrastructures internationally.

CONCLUSION

Men with PCa receiving ADT are a vulnerable high-need patient group. As yet an effective way to deliver holistic survivorship care to improve HR-QoL in this patient population has not yet been identified. The study will provide effectiveness and implementation data to address this knowledge gap and inform the potential for implementation of *PCEssentials* at scale.

For peer review only

Author contributions

JD, SC, NH, AG, RN, DS, HT, DG, PH, MP, DC, SE and SS contributed to study conception or study design. SS, SC, DG, RN, NH, JD and AG were on the steering committee that developed the intervention. HT designed the economic component of the study. JD, SC, NH, AG, RN, DS, HT, DG, PH, MP, DC, SE, and SS provided substantial input into the development of the protocol or revising it critically for important intellectual content. AG drafted the manuscript with contributions from RN, DS, HT, DG, PH, MP, DC, SE, SS, NH, SC, JD. All authors contributed to, read and approved the final manuscript.

Funding statement

This work is supported by the National Health and Medical Research Council (NH&MRC) grant number APP2006528.

Competing interests statement

No conflicts of interest.

For peer review only

Figure 1. Prostate Cancer Survivorship Essentials Framework(23)

Figure 2. Study Diagram

For peer review only

References

1. Australian Institute of Health and Welfare. Cancer data in Australia [online]. 2023. https://www.aihw.gov.au/reports/cancer/cancer-data-in-australia/contents/overview-of-cancer-in-australia-2023 (accessed 13 December 2023).

2. Ralph N, Ng SK, Zajdlewicz L, et al. Ten-year quality of life outcomes in men with prostate cancer. Psycho-Oncology 2020;29(2):444-9.

3. Chipperfield K, Fletcher J, Millar J, et al. Predictors of depression, anxiety and quality of life in patients with prostate cancer receiving androgen deprivation therapy. Psycho-Oncology 2013;22(10):2169-76.

4. Nguyen C, Lairson DR, Swartz MD, et al. Risks of Major Long-Term Side Effects Associated with Androgen-Deprivation Therapy in Men with Prostate Cancer. Pharmacotherapy 2018;38(10):999-1009.

5. Mazariego CG, Egger S, King MT, et al. Fifteen year quality of life outcomes in men with localised prostate cancer: population based Australian prospective study. BMJ 2020;371:m3503.

6. Smith DP, King MT, Egger S, et al. Quality of life three years after diagnosis of localised prostate cancer: population based cohort study. BMJ 2009;339:B4817.

7. Casey RG, Corcoran NM, Goldenberg SL. Quality of life issues in men undergoing androgen deprivation therapy: a review. Asian Journal of Andrology 2012;14(2):226-31.

8. Edmunds K, Tuffaha H, Galvão DA, et al. Incidence of the adverse effects of androgen deprivation therapy for prostate cancer: a systematic literature review. Supportive Care in Cancer 2020;28(5):2079-93.

9. Rhee H, Gunter JH, Heathcote P, et al. Adverse effects of androgen-deprivation therapy in prostate cancer and their management. BJU International 2015;115(Suppl 5):3-13.

10. Australian Cancer Network Management of Metastatic Prostate Cancer Working Party. *Clinical Practice Guidelines for the Management of Locally Advanced and Metastatic Prostate Cancer*. Sydney: Cancer Council Australia and Australian Cancer Network, 2010.

11. Ng HS, Koczwara B, Roder D, et al. Development of comorbidities in men with prostate cancer treated with androgen deprivation therapy: an Australian population-based cohort study. Prostate Cancer and Prostatic Diseases 2018;21(3):403-10.

12. Paterson C, Primeau C, Howard N, et al. Experiences of Unmet Need and Access to Supportive Care for Men Receiving Androgen Depriviation Therapy for Prostate Cancer: A Bi-national Study. Seminars in Oncology Nursing 2020;36(4):151049.

13. Watts S, Leydon G, Birch B, et al. Depression and anxiety in prostate cancer: a systematic review and meta-analysis of prevalence rates. BMJ Open 2014;4(3):e003901.

14. Carlsson S, Sandin F, Fall K, et al. Risk of suicide in men with low-risk prostate cancer. European Journal of Cancer 2013;49(7):1588-99.

15. Paterson C, Kata SG, Nandwani G, et al. Unmet Supportive Care Needs of Men With Locally Advanced and Metastatic Prostate Cancer on Hormonal Treatment: A Mixed Methods Study. Cancer Nursing 2017;40(6):497-507.

16. Mazariego CG, Juraskova I, Campbell R, et al. Long-term unmet supportive care needs of prostate cancer survivors: 15-year follow-up from the NSW Prostate Cancer Care and Outcomes Study. Supportive Care in Cancer 2020;28(11):5511-20.

For peer review only

17. Gray RE, Wassersug RJ, Sinding C, et al. The experiences of men receiving androgen deprivation treatment for prostate cancer: a qualitative study. The Canadian Journal of Urology 2005;12(4):2755-63.

18. Skolarus TA, Wolf AMD, Erb NL, et al. American Cancer Society prostate cancer survivorship care guidelines. CA Cancer J Clin 2014;64(4):225-49.

19. Narayan V, Harrison M, Cheng H, et al. Improving research for prostate cancer survivorship: A statement from the Survivorship Research in Prostate Cancer (SuRECaP) working group. Urol Oncol 2020;38(3):83-93.

20. Dunn J, Ralph N, Green A, et al. Contemporary consumer perspectives on prostate cancer survivorship: Fifty voices. Psycho-Oncology 2020;29(3):557-63.

21. Armstrong MJ, Bloom JA. Patient involvement in guidelines is poor five years after institute of medicine standards: review of guideline methodologies. Res Involv Engagem 2017;3:19.

22. Crawford-Williams F, March S, Goodwin BC, et al. Interventions for prostate cancer survivorship: A systematic review if reviews. Psycho-Oncology 2018;27(10):2339-48.

23. Dunn J, Green A, Ralph N, et al. Prostate Cancer Survivorship Essentials Framework: Guidelines for Practitioners. BJU Int 2021;128(Suppl 3):18-29.

24. Curran GM, Bauer M, Mittman B, et al. Effectiveness-implementation hybrid designs: combining elements of clinical effectiveness and implementation research to enhance public health impact. Med Care 2012;50(3):217-26.

25. Proctor E, Silmere H, Raghavan R, et al. Outcomes for implementation research: Conceptual distinctions, measurement challenges, and research agenda. Administration and Policy in Mental Health Services Research 2011;38(2):65-76.

26. Altman DG, Schulz KF, Moher D, et al. The Revised CONSORT Statement for Reporting Randomized Trials: Explanation and Elaboration. Annals of Internal Medicine 2001;134(8):663-94.

27. The National Health and Medical Research Council, The Australian Research Council, Universities Australia. *National Statement in Ethical Conduct in Human Research 2007*. Canberra: Commonwealth of Australia, 2007.

28. The National Health and Medical Research Council, The Australian Research Council, Universities Australia. *Australian Code for the Responsible Conduct of Research 2018*. Canberra: Commonwealth of Australia, 2018.

29. Chambers SK, Ferguson M, Gardiner R, et al. ProsCan for Men: Randomised controlled trial of a decision support intervention for men with localised prostate cancer. BMC Cancer 2008;8(1):207.

30. Chambers SK, Occhipinti S, Schover L, et al. A randomised controlled trial of a couples-based sexuality intervention for men with localised prostate cancer and their female partners. Psycho-Oncology 2015;24(7):748-56.

31. Zajdlewicz L, Hyde M, Lepore S, et al. Health-related quality of life after diagnosis of locally-advanced or advanced prostate cancer: A longitudinal study. Cancer Nursing 2017;40(5):412-9.

32. Kiosses DN, Alexopoulos GS. Problem-Solving Therapy in the Elderly. Current Treatment Options in Psychiatry 2014;1(1):15-26.

33. Roesch SC, Adams L, Hines A, et al. Coping with prostate cancer: a meta-analytic review. Journal of Behavioral Medicine 2005;28(3):281-93.

For peer review only

34. Chambers S, Hyde M, Oliffe J, et al. Measuring Masculinity in the Context of Chronic Disease. Psychology of Men & Masculinity 2015;17(3):228-42.

35. Chambers S. Facing the Tiger: A Survivorship Guide for Men with Prostate Cancer and their Partners. Brisbane, Australia: Australian Academic Press 2020.

36. Chambers SK, Zajdlewicz L, Youlden DR, et al. The validity of the distress thermometer in prostate cancer populations. Psycho-Oncology 2014;23(2):195-203.

37. Hayes SC, Newton RU, Spence RR, et al. The Exercise and Sports Science Australia position statement: Exercise medicine in cancer management. Journal of Science and Medicine in Sport 2019;22(11):1175-99.

38. Shapiro CL. Cancer Survivorship. The New England Journal of Medicine 2018;379(25):2438-50.

39. Chambers S, Hyde M, Smith D, et al. New Challenges in Psycho-Oncology Research III: A Systematic Review of Psychological Interventions for Prostate Cancer Survivors and Their Partners: Clincial and Research Implications. Psycho-Oncology 2017;26(7):873-913.

40. Weiner BJ, Lewis CC, Stanick C, et al. Psychometric assessment of three newly developed implementation outcome measures. Implementation Science 2017;12(1):108.

41. Hatcher RL, Gillaspy JA. Development and validation of a revised short version of the working alliance inventory. Psychotherapy Research 2006;16(1):12-25.

42. Cella DF, Tulsky DS, Gray G, et al. The Functional Assessment of Cancer Therapy scale: development and validation of the general measure. Journal of Clinical Oncology 1993;11(3):570-9.

43. Richardson J, Elsworth G, Iezzi A, et al. *Increasing the sensitivity of the AQoL inventory for evaluation of interventions affecting mental health*. Melbourne, Australia: Monash University, Business and Economics, Centre for Health Economics, 2011.

44. Ware JE Jnr, Gandek B. Overview of the SF-36 Health Survey and the International Quality of Life Assessment (IQOLA) Project. Journal of Clinical Epidemiology 1998;51(11):903-12.

45. Galvão DA, Taaffe DR, Spry N, et al. Combined resistance and aerobic exercise program reverses muscle loss in men undergoing androgen suppression therapy for prostate cancer without bone metastases: a randomized controlled trial. Journal of Clinical Oncology 2010;28(2):340-7.

46. Foster C, Breckons M, Cotterell P, et al. Cancer survivors' self-efficacy to self-manage in the year following primary treatment. Journal of cancer survivorship: research and practice 2015;9(1):11-9.

47. Spitzer RL, Kroenke K, Williams JB, et al. A brief measure for assessing generalized anxiety disorder: the GAD-7. Archives of Internal Medicine 2006;166(10):1092-7.

48. Kroenke K, Spitzer RL, Williams JB. The PHQ-9: validity of a brief depression severity measure. J Gen Intern Med 2001;16(9):606-13.

49. Bastien CH, Vallieres A, Morin CM. Validation of the Insomnia Severity Index as an outcome measure for insomnia research. Sleep Medicine 2001;2(4):297-307.

50. Stein KD, Jacobsen PB, Blanchard CM, et al. Further validation of the multidimensional fatigue symptom inventory-short form. Journal of Pain and Symptom Management 2004;27(1):14.

51. Godin G. The Godin-Shephard leisure-time physical activity questionnaire. The Health Fitness Journal of Canada 2011;4(1):18-22.

For peer review only

52. Galvão DA, Taaffe DR, Hayne D, et al. Weight loss for overweight and obese patients with prostate cancer: a study protocol of a randomised trial comparing clinic-based versus Telehealth delivered EXercise and nutrition intervention (the TelEX trial). BMJ Open 2022;12(6).

53. Faller H, Scuhler M, Richard M, et al. Effects of Psycho-Oncologic Interventions on Emotional Distress and Quality of Life in Adult Patients with Cancer: Systematic Review and Meta-Analysis. Journal of Clinical Oncology 2013;31(6):782-93.

54. Hsieh HF, Shannon SE. Three approaches to qualitative content analysis. Qualitative health research 2005;15(9):1277-88.

55. Guetterman TC, Fetters MD, Creswell JW. Integrating Quantitative and Qualitative Results in Health Science Mixed Methods Research through Joint Displays. Ann Fam Med 2015;13(6):554-61.

56. Grimm SE, Stevens JW, Dixon S. Estimating Future Health Technology Diffusion Using Expert Beliefs Calibrated to an Established Diffusion Model. Value Health 2018;21(8):944-50.

57. Bellg AJ, Borrelli B, Resnick B, et al. Enhancing treatment fidelity in health behaviour change studies: best practices and recommendations from the NIH Behaviour Change Consortium. Health Psychol 2004;23(5):443-51.

For peer review only

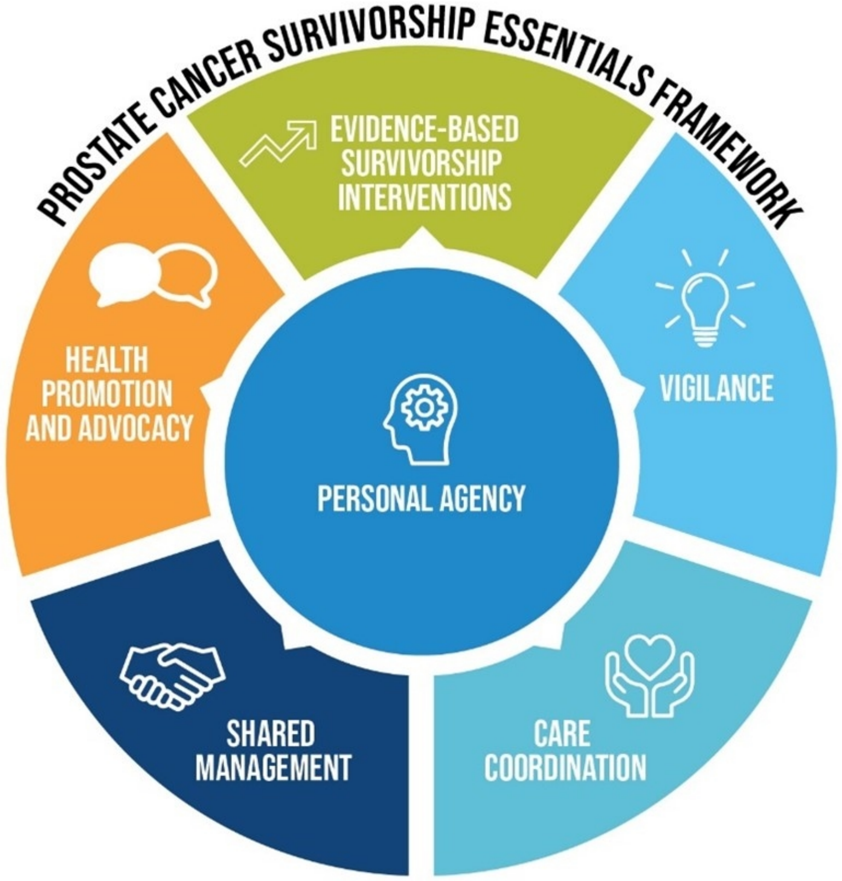

Figure 1. Prostate Cancer Survivorship Essentials Framework23

105x105mm (220 x 220 DPI)

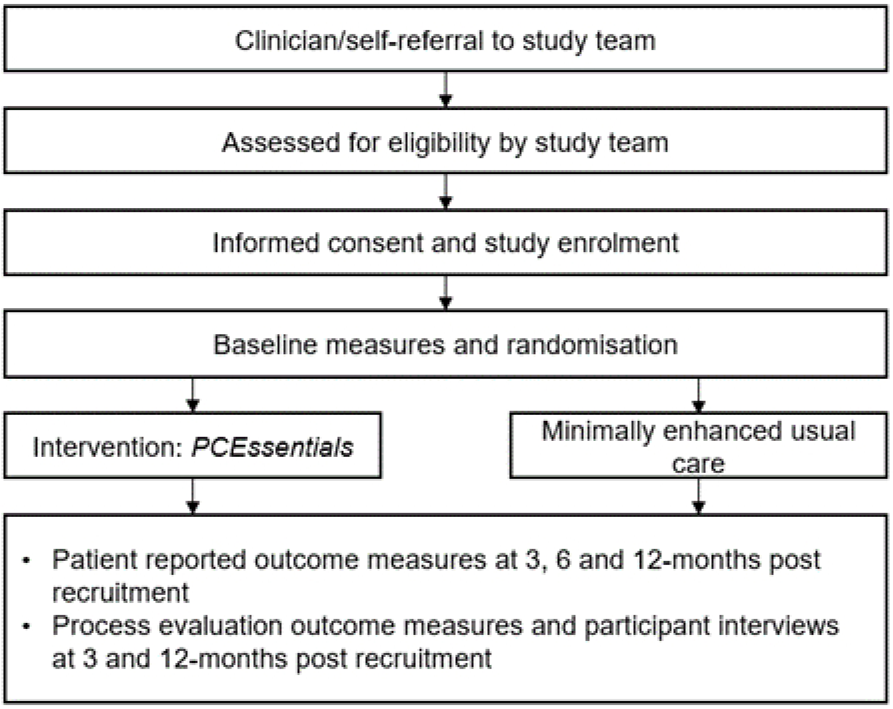

Figure 2. Study Diagram

322x243mm (38 x 38 DPI)

Supplementary File 1. Question route for interviews

Patient Question Route – Semi-Structured interviews; based on relevant Conceptual Framework for Implementation Outcomes[1]

Italics: Question prompts

Construct/Outcome	Questions
How an individual feels about taking part in an intervention	How did you feel about taking part in *PCEssentials*?<br>• *When you first heard about it*<br>• *While you were taking part*
The extent to which the participant understands the intervention, and how the intervention works	How would you describe what *PCEssentials* was about?
The participant's confidence that they can perform the behaviour(s) required to participate in the intervention	How confident were you that you could do what you needed to take part in *PCEssentials*?<br>• *Access and use resources*<br>• *Contact the intervention nurse*<br>• *Complete the homework*
The perceived amount of effort that is required to participate in the intervention	Do you think *PCEssentials* is easier or harder than coming to the hospital/clinic for care?<br>• *In what way is it easier/harder?*
The extent to which benefits, profits, or values must be given up to engage in an intervention	Did you feel you had to give anything up/miss out on anything to take part in *PCEssentials*? (out of pocket expenses, quality of care)<br>• *Can you give some examples?*<br>• *Do you think the quality of care you received/costs was the same as coming to the hospital/clinic for care?*
The extent to which the intervention has good fit with an individual's value system	Does this type of virtual care meet your needs?<br>• *Why or why not?*<br>• *What could be changed to meet your needs?*<br>• *What was it that really helped meet your needs?*
The extent to which the intervention is perceived as likely to achieve its purpose	Looking back at *PCEssentials* since you started, how effective do you think it is overall?<br>• *In what way is it effective/not effective?*<br>Can you give me an example of something you really liked/disliked about *PCEssentials*?<br>How could *PCEssentials* be improved?<br>Is there anything else you wanted to say about *PCEssentials*?

For peer review only

Clinical Stakeholder Question Route – Semi-Structured interviews; based on relevant Conceptual Framework for Implementation Outcomes[1]

Italics: Question prompts

Construct/Outcome	Questions
How an individual feels about conducting/taking part in an intervention	What were your thoughts about the *PCEssentials* study?<br>• *When you first heard about it*<br>• *While you were recruiting*
The extent to which the participant understands the intervention, and how the intervention works	What is your understanding about how *PCEssentials* works?
The participant's confidence that they can perform the behaviour(s) required to deliver/take part in the intervention	How confident were you that you could do what you needed to deliver/take part in *PCEssentials*?<br>• *Recruitment*<br>• *Conducting the intervention sessions*<br>• *Identifying triggers for care escalation/managing deterioration*
The perceived amount of effort that is required to deliver/take part in the intervention	How burdensome is *PCEssentials* to deliver/take part in compared to usual care?<br>• *In what way is it less/more burdensome?*
The extent to which benefits, profits, or values must be given up to deliver/take part in an intervention	As a clinician do you feel you had to give anything up to deliver/take part in *PCEssentials*?<br>• *Can you give some examples?*<br>Do you think the quality of care delivered in *PCEssentials* differs from usual care?<br>• *Can you give some examples?*<br>From a cost perspective to your service, are there any advantages/disadvantages to the *PCEssentials* model compared to usual care?
The extent to which the intervention has good fit with an individual's value system	Does *PCEssentials* meet your needs as a clinician?<br>• *Why or why not?*<br>• *What could be changed to meet your needs?*<br>• *What was it that helped meet your needs?*
The extent to which the intervention is perceived as likely to achieve its purpose	Looking back at the program since it started, how effective do you think *PCEssentials* is overall?<br>• *In what way is it effective/not effective?*<br>• *Clinician perspective*<br>• *Patient needs*

For peer review only

	Can you give me an example of something you really liked/disliked about *PCEssentials?*<br>How could *PCEssentials* be improved?<br>Is there anything else you wanted to say about *PCEssentials*?

1. Proctor E, Silmere H, Raghavan R, Hovmand P, Aarons G, Bunger A, et al. Outcomes for implementation research: conceptual distinctions, measurement challenges, and research agenda. Administration and policy in mental health. 2011;38(2):65-76.

For peer review only

Prostate Cancer Survivorship Essentials for Men with Prostate Cancer on Androgen Deprivation Therapy (PCEssentials Hormone Therapy Study)

Participant Information Sheet - Patients

**Title**	Prostate Cancer Survivorship Essentials for Men with Prostate Cancer on Androgen Deprivation Therapy: An Effectiveness-Implementation Hybrid (Type 1) Trial of a Tele-Based Nurse-Led Survivorship Care Intervention
**Short Title**	*PCEssentials Hormone Therapy Study*
**Coordinating Principal Investigator**	Professor Jeff Dunn AO

1. Would you like to take part in this study?

You are invited to take part in this research study because you have prostate cancer and are starting, or are planning to start, Androgen Deprivation Therapy (ADT)/hormone therapy. We want to implement and test a new survivorship care intervention delivered by a Prostate Cancer Specialist Nurse via tele-health to identify if it improves the quality of life for men on ADT/hormone therapy and their ability to support their own health and wellbeing. We have called this the *PCEssentials* Intervention. This Participant Information Sheet and Consent Form tells you about the research study to help you decide if you want to take part. It explains the *PCEssentials* Intervention, the study surveys, and the data collection involved. Knowing what is involved, and the potential benefits and risks to you, will help you decide if you want to take part in the research. Please read this information carefully. Ask questions about anything that you don't understand or want to know more about. You might also want to talk to a relative, a friend or your GP before you make up your mind. If you decide to go ahead, we will ask you to sign the 'Participant Consent Form' at the end of this document.

2. What is the purpose of this research?

In this study we test a Prostate Cancer Specialist Nurse-led survivorship intervention for men on ADT/hormone therapy called *PCEssentials*. The study has been designed to fill the survivorship care gap for men on ADT/hormone therapy. An essential part of any quality cancer care is survivorship care. Survivorship care starts at the time of cancer diagnosis and continues throughout the lifespan. The goal of survivorship care is to provide personalised care and support self-management with a strong focus on the patients' needs and experiences. This includes supporting a person through the cancer diagnosis, making decisions about treatment, managing side effects, and maintaining health and wellbeing during and after treatment. Unfortunately survivorship care is often not delivered well, or easily accessible, especially for people living in regional and remote areas, and there is currently no survivorship care model for men on ADT/hormone therapy.

The *PCEssentials* Intervention is a survivorship care model for men on ADT/hormone therapy that will provide one-to-one psychological support, treatment education, tailored strategies to help manage distress, decision making and self-management. It will also include a home-based exercise activity program. The research will identify if this way of providing survivorship care to men on ADT/hormone therapy improves their quality of life and ability to support their own health and wellbeing.

This research has been initiated by Professor Jeff Dunn AO –Chief of Mission and Head of Research, Prostate Cancer Foundation of Australia and Professor and Chair of Cancer Survivorship at the University of Southern Queensland, and

For peer review only - http://bmjopen.bmj.com/site/about/guidelines.xhtml

has been funded by the National Health and Medical Research Council (NHMRC) which is administered by the federal Department of Health.

3. Your participation is voluntary

Your participation in this study is completely voluntary. If you do not want to take part in this study, you do not have to. You should feel under no obligation to participate in this study. If you decide to take part and later change your mind, you are free to withdraw at any stage. Choosing not to take part in this study, or if you choose to take part and then later withdraw, will not affect your current and future medical care in any way. Your choice will not affect your relationship with those treating you, or with any institutions involved in this research.

4. Your withdrawal from the study

If you decide to withdraw from the study, you will be offered the usual care delivered by your specialist team. You can choose to withdraw from:

- the whole study: where we stop collecting any data about you **OR**
- part of the study involving your active participation (i.e., completing questionnaires, participating in the interview)

After you have started your participation in this research study, you are under no obligation to continue, and can change your mind at any time about participating in the research. People withdraw from studies for various reasons, and you do not need to provide a reason. You can withdraw at any time by contacting the research team or completing and signing the 'Participant Withdrawal of Consent Form'. This form is located at the end of this document. If you withdraw from the study, you will be able to choose whether the study will destroy or be-able to retain the information collected about you. You should only choose **one** of these options. Where both boxes are ticked in error or neither box is ticked, the study will destroy all information it has collected about you.

5. What does participation in this research involve?

Sometimes we need to compare different models of care to find out which is the best. To do this, we put people into groups and give each group a different model of care. We can then compare the groups to see if one model of care is better than the other. To make sure the groups are the same, each participant is put into a group by chance (random): like flipping a coin. This is called a randomised controlled study and it is designed to make sure we can interpret the results in a fair and appropriate way and to avoid doctors or participants jumping to conclusions about what is best.

In the *PCEssentials Hormone Therapy* Study, we will randomly allocate about 236 men with prostate cancer who are starting, or are planning to start, ADT/hormone therapy from treatment centres across Australia to receive the *PCEssentials* Intervention or usual care at the discretion of a patients specialist team (the Usual Care group). You will have a 50% chance of being in either the *PCEssentials* Intervention group or the Usual Care group. We will follow everyone up to 12 months after recruitment to the study.

If you decide you want to take part in the research study, you will be asked to sign the Consent Form. By signing it you are telling us that you:

- ✓ Understand what participation in *PCEssentials Hormone Therapy* Study will involve
- ✓ Consent to take part in the study as described
- ✓ Give permission for the *PCEssentials Hormone Therapy* Study team to access your personal information during the study
- ✓ Consent to the use of your personal and health information as described.

For peer review only - http://bmjopen.bmj.com/site/about/guidelines.xhtml

✓ Understand that you will be randomly allocated to one of the two models of survivorship care.

There are no costs associated with participating in this research study, nor will you be paid.

5.1 What do I need to do?

If you agree to participate, a member of the *PCEssentials Hormone Therapy* Study team will provide you with detailed information about the study. You will first be asked to complete the study surveys to tell us about yourself. You will then be randomly allocated to one of two study groups: *PCEssentials* Intervention (the new model of care) or specialist-led model of survivorship care (the current practice). It is important for you to understand that we do not know which model of care will be better for you or other men with prostate cancer on ADT/hormone therapy, which is the reason we are conducting this research, thus it is important to follow the model of care that you are randomly assigned. This will help us answer important research questions to improve cancer survivorship care for men with prostate cancer on ADT/hormone therapy across Australia.

If you are allocated to the *PCEssentials* Intervention, you will receive a five-session psychoeducation program delivered by a Prostate Cancer Specialist Nurse via tele-health which includes four sessions over three months and a booster session at six months after the first session. The nurse-led intervention will include five modules covering:

- ✓ Psycho-education with tailored distress management strategies;
- ✓ Decision support;
- ✓ Treatment education with self-management and skills training for symptom effects, including exercise/physical activity resources and support;
- ✓ Communicating with health professionals including a referral pathway to your general practitioner for a Chronic Disease Management plan (CDM)

You will also be provided with a home-based exercise activity program and be encouraged to seek at least one planning session with an Accredited Exercise Physiologist (AEP) within your treatment team, which may be by tele-health as appropriate.

If you are allocated to Usual Care, you will continue to be cared for by your specialist team as usual minimally enhanced by a package of resources containing patient education materials about the use of ADT/hormone therapy to treat prostate cancer; and advice about referral to support services.

5.2 Complete study surveys (20-30 minutes each)

Whether you receive the *PCEssentials* Intervention or Usual Care, a member of the *PCEssentials Hormone Therapy* Study team will contact you at four points during the active study period (12 months from when you begin the study) to ask you to complete the study surveys so that we can find out more about you, your health, and your healthcare experience before you start the study, and at 3 months, 6 months, and 12 months after you start the study. The *PCEssentials Hormone Therapy* Study team will send paper surveys delivered to you through Australia Post. The surveys will take about 20-30 minutes each to complete (depending on how you choose to complete them) and a little longer at the first time point. The *PCEssentials Hormone Therapy* Study team may send reminders to you via post, phone call, text, or email, as required.

5.3 Additional opportunity

There is an additional opportunity for involvement in this research study, <u>which is optional:</u>

- **Interview:** A member of the *PCEssentials Hormone Therapy* Study team may contact you during the research study to invite you to participate in a one-off, individual interview to find out about your experience of participating in this research study. This interview is completely voluntary and can be stopped at any time. It

For peer review only - http://bmjopen.bmj.com/site/about/guidelines.xhtml

will be audio-recorded to allow the research team to reflect and analyse the interview data later. The interview should take no longer than about 30 minutes.

6. What are the alternatives to participation?

You do not have to take part in this research to receive care. Other options are available. Whether or not you choose to participate in this research, you will still be offered the usual care delivered by your specialist team. The *PCEssentials Hormone Therapy* Study team will discuss these options with you before you decide whether to take part in this research study. You can also discuss the options with your local doctor.

7. What are the possible benefits of taking part?

We cannot guarantee that you will receive any benefits from this research; however, possible benefits may include an improvement in your health and experience of care from the survivorship care approach (i.e., the intervention we are trialling). Your taking part in this project will provide us with important information about survivorship care for men on ADT/hormone therapy, which will be helpful to patients in the future.

8. What are the possible risks and disadvantages of taking part?

There are minimal risks associated with your participation in this study. There is a very small possibility that you might experience some distress because the study surveys cover personal questions relating to possible symptoms, your cancer, and your experience of care. If you receive the *PCEssentials* Intervention, you may experience some distress while discussing issues relating to treatment, side-effects and psychosocial impact during the intervention. You may also experience side-effects arising from changes in physical activity (such as muscle soreness) if you choose to take part in the exercise component of the intervention. If you do become upset because of the research study, you should contact the *PCEssentials Hormone Therapy* Study team, or talk to your Prostate Cancer Specialist Nurse or doctors who will be able to arrange for counselling or other appropriate support.

9. How will you use any tissues or samples you take from me?

We will not collect any tissues or samples from you in this study.

9.1 Will you be doing any genetic tests?

There are no genetic tests in this study.

10. What if new information arises during this research study?

Sometimes during a research study, new information becomes available about the treatment that is being studied. If this happens, the *PCEssentials Hormone Therapy* Study team will tell you about it and discuss with you whether you want to continue in the research study. If you decide to withdraw, the *PCEssentials Hormone Therapy* Study team will arrange for your regular health care to continue. If you decide to continue in the research study, you will be asked to sign an updated consent form.

11. What happens when the research study ends?

We will not contact you again after your 12-month active participation period (which is the 12 months after you begin the study). If you would like to receive a copy of the results at the end of the *PCEssentials Hormone Therapy* Study, please indicate this on the Consent Form or contact the *PCEssentials Hormone Therapy* Study team and we will send this with our compliments.

12. Could the researchers stop the study early?

Yes, if it does, the *PCEssentials Hormone Therapy* Study team will let you know and explain the reason behind the decision. If the study stops early, you will continue to be cared for by your specialist team as usual.

For peer review only - http://bmjopen.bmj.com/site/about/guidelines.xhtml

Prostate Cancer Foundation of Australia

PROSTATE CANCER SURVIVORSHIP ESSENTIALS

University of Southern Queensland

13. Privacy, Confidentiality and Disclosure of Information

We will keep all personal information confidential and securely stored. The electronic data we collect about you will be stored on a secure server hosted by the University of Southern Queensland. Hard copies of research data will be stored securely at the Prostate Cancer Foundation of Australia (PCFA) St Leonards office. Any information obtained in connection with this project that could identify you will remain confidential. Only authorised study staff will have access to these materials. It will only be disclosed with your permission, except as required by law. In any publication, information will be provided in such a way that you cannot be identified. Data will be stored for 25 years in accordance with the National Statement (2007) and institutional policy.

Australian privacy law gives you the right to request access to your information that the researchers have collected and stored. The law also gives you the right to request corrections to any information about you that you disagree with. Please contact the study team (see page 5 of this document) if you would like to access your information.

So that we can contact you to take part in an interview, we will ask you to provide an email address or a phone number. This will not be linked to any information we have about you in connection to the project.

14. Who is organising and funding the research?

This research study is being conducted by Professor Jeff Dunn AO, University of Southern Queensland, in partnership with the Prostate Cancer Foundation Australia (PCFA), Cancer Council Queensland (CCQ), Australian Prostate Centre (APC), Ipswich West Moreton Hospital Health Service (WMHHS) GenesisCare, Icon Group, Healthy Male, and the Union for International Cancer Control (UICC). The University of Southern Queensland will receive a payment from the NHMRC administered by the federal Department of Health for undertaking this research. No member of the *PCEssentials Hormone Therapy* Study team will receive any financial benefit from your involvement in this research study (other than their ordinary wages).

15. Who has reviewed the research study?

All research in Australia involving humans is reviewed by an independent group of people called a Human Research Ethics Committee (HREC). The ethical aspects of this research study have been approved by the Metro South HREC. This research study will be carried out according to the National Statement on Ethical Conduct in Human Research (2007). This statement has been developed to protect the interests of people who agree to participate in human research studies.

16. What if I have a question or need to make a complaint?

We have included several contacts for you below. The person you may need to contact will depend on the nature of your query.

For questions about the *PCEssentials Hormone Therapy* Study, you can contact the *PCEssentials Hormone Therapy* Study team:

- Coordinating Principal Investigator: Professor Jeff Dunn AO, *[phone TBA]*
- Central Management Team: Dr Anna Green, *[phone, email TBA]*

To talk to someone at your treatment centre:

- Principal Investigator *[Study Site]*: *[Name & contact number]*

For peer review only - http://bmjopen.bmj.com/site/about/guidelines.xhtml

If you wish to discuss the study with someone who is not directly involved, particularly in relation to matters concerning complaints about the conduct of the study, or your rights as a participant, you can contact:

Lead HREC Office	Metro South Health and Hospital Services (MSHHS)
Contact Person	HREC Coordinator
Telephone	+61 7 3443 8049
Email	MSH-Ethics@health.qld.gov.au
HREC Reference Number	*[HREC approval number]*

Site HREC Office	*[Institute Ethics Office]*
Contact Person	*[Contact Person]*
Telephone	*[Telephone]*
Email	*[Email]*
HREC Reference Number	*[HREC approval number]*

17. The Participant Consent Form

Sign the consent form only after you have made up your mind to take part in this study. You must be provided with a signed and dated copy of the participant information and consent form for your personal record.

For peer review only

Prostate Cancer Survivorship Essentials for Men with Prostate Cancer on Androgen Deprivation Therapy (PCEssentials Hormone Therapy Study)

Participant Consent Form

**Title**	Prostate Cancer Survivorship Essentials for Men with Prostate Cancer on Androgen Deprivation Therapy: An Effectiveness-Implementation Hybrid (Type 1) Trial of a Tele-Based Nurse-Led Survivorship Care Intervention
**Short Title**	*PCEssentials Hormone Therapy Study*
**Coordinating Principal Investigator**	Professor Jeff Dunn AO

Declaration by Participant

I have read, or have had read to me, and I understand the Participant Information Sheet and Consent Form.

I understand the purposes, procedures and risks of the research described in the Participant Information Sheet.

I have had an opportunity to ask questions and I am satisfied with the answers I have received.

I freely agree to participate in this research study as described and understand that I am free to withdraw at any time during the study without affecting my future health care.

I consent to my treating doctor/s being notified of my participation in this study and any clinically relevant information noted by the *PCEssentials Hormone Therapy* Study team in the conduct of the study.

I understand that I will be given a signed copy of this document to keep for my own records. We may ask you to participate in a future related study, or to obtain additional information or clarification related to your participation in this study. **Please indicate below** whether you are willing to be contacted about any future research studies.

I agree to the research team using, reproducing, and disclosing audio-recordings as explained in the Participant Information Sheet/Consent Form for Patients.

I agree to be audio-recorded and understand that, subject to any constraints requested below, recordings may be used in presentations and publications for educational and research purposes.

30-minute Interview (Optional)

☐ Yes, I agree to be contacted and invited to participate in the interview

☐ No, I do not want to be contacted to be invited to participate in the interview

Future Studies

☐ Yes, I agree to be contacted about future research studies

☐ No, I do not want to be contacted about future research studies

For peer review only - http://bmjopen.bmj.com/site/about/guidelines.xhtml

Prostate Cancer Foundation of Australia
PROSTATE CANCER SURVIVORSHIP ESSENTIALS
University of Southern Queensland

Study Results

☐ Yes, I would like to receive a copy of the study results and acknowledge that these will be provided in aggregate as a summary (individual results will not be available)

☐ No, I do not want a copy of the study results

Participant

Signature ___________________________ Date ___________________________

Name (please print) ___________________________

For peer review only

Declaration by senior researcher

I have given a verbal explanation of the study, its procedures and risks and I believe that the participant has understood that explanation.

Signature ___________________________ Date ___________________________

Name (please print) ___________________________

For peer review only - http://bmjopen.bmj.com/site/about/guidelines.xhtml

Prostate Cancer Foundation of Australia PROSTATE CANCER SURVIVORSHIP ESSENTIALS University of Southern Queensland

Prostate Cancer Survivorship Essentials for Men with Prostate Cancer on Androgen Deprivation Therapy (PCEssentials Hormone Therapy Study)

Participant Information Sheet – Clinical Stakeholders

**Title**	Prostate Cancer Survivorship Essentials for Men with Prostate Cancer on Androgen Deprivation Therapy (ADT): An Effectiveness-Implementation Hybrid (Type 1) Trial of a Tele-Based Nurse-Led Survivorship Care Intervention
**Short Title**	*PCEssentials Hormone Therapy Study*
**Coordinating Principal Investigator**	Professor Jeff Dunn AO

1. Would you like to take part in this study?

You have been invited to take part in this study because you are part of the clinical and/or administrative teams involved in recruitment or delivery of the *PCEssentials* Intervention. We want to implement, test and evaluate a new survivorship care intervention delivered by a Prostate Cancer Specialist Nurse via tele-health (*PCEssentials)* to identify if it improves the quality of life for men on androgen deprivation therapy (ADT) and their ability to support their own health and wellbeing.

This Participant information and consent handout contains information about the *PCEssentials Hormone Therapy* study. Its purpose is to explain to you as openly and clearly as possible all the procedures involved in this project before you decide whether or not to take part.

Please read this Participant information and consent handout carefully. Please free to ask questions about any information in the handout or about the project. You will be asked to sign the Consent Form if you agree to participate. By signing the Consent Form, you indicate that you understand the information and that you give your consent to participate in the research project.

2. What is the purpose of this research?

In this study we test and evaluate a Prostate Cancer Specialist Nurse-led survivorship intervention for men on ADT (*PCEssentials*). The study has been designed to fill the survivorship care gap for men on ADT. An essential part of any quality cancer care is survivorship care. Survivorship care starts at the time of cancer diagnosis and continues throughout the lifespan. The goal of survivorship care is to provide personalised care and support self-management with a strong focus on the patients' needs and experiences. This includes supporting a person through the cancer diagnosis, making decisions about treatment, managing side effects, and maintaining health and wellbeing during and after treatment. Unfortunately survivorship care is often not delivered well, or easily accessible, especially for people living in regional and remote areas, and there is currently no survivorship care model for men on ADT.

The *PCEssentials* Intervention is a survivorship care model for men on ADT that will provide one-to-one psychological support, treatment education, tailored strategies to help manage distress, decision making and self-management. It will also include a home-based exercise activity program. The research will identify if this way of providing survivorship care to men on ADT improves their quality of life and ability to support their own health and wellbeing.

This research has been initiated by Professor Jeff Dunn AO –Chief of Mission and Head of Research, Prostate Cancer Foundation of Australia and Professor and Chair of Cancer Survivorship at the University of Southern Queensland, and has been funded by the National Health and Medical Research Council (NHMRC) which is administered by the federal Department of Health.

For peer review only - http://bmjopen.bmj.com/site/about/guidelines.xhtml

Prostate Cancer Foundation of Australia

PROSTATE CANCER SURVIVORSHIP ESSENTIALS

University of Southern Queensland

3. Your participation is voluntary

Your participation in this study is completely voluntary. If you do not want to take part in this study, you do not have to, and are not obliged to. If you decide to take part and later change your mind, you are free to withdraw at any stage. Choosing not to take part in this study, or if you choose to take part and then later withdraw, will not affect your current employment or relationship with any institutions involved in this research.

4. Your withdrawal from the study

After you have started your participation in this research study, you are under no obligation to continue, and can change your mind at any time about participating in the research. People withdraw from studies for various reasons, and you do not need to provide a reason. You can withdraw at any time by notifying the research team or completing and signing the 'Participant Withdrawal of Consent Form'. This form is located at the end of this document. If you withdraw from the study, you will be able to choose whether the study will destroy or be-able to retain the information collected about you. You should only choose **one** of these options. Where both boxes are ticked in error or neither box is ticked, the study will destroy all information it has collected about you.

5. What does participation in this research involve?

If you agree to participate in this study, you will be asked to complete two short online surveys (approximately five minutes to complete) and take part in a semi-structured interview. Interviews will be conducted by phone or videoconference by a member of the research team to explore your perceptions of the acceptability and feasibility of *PCEssentials*. It will be audio-recorded to allow the research team to reflect and analyse the interview data later. This interview will take 15-30 minutes to complete.

There are no costs associated with participating in this research study, nor will you be paid.

6. What are the possible benefits of taking part?

We cannot guarantee that you will receive any direct benefits from this research; however, your participation in this project will provide us with important information about survivorship care for men on ADT, which will be helpful to clinicians, health services and patients in the future.

7. What are the possible risks and disadvantages of taking part?

There are minimal risks associated with your participation in this study. The primary risk is the inconvenience related to the time it takes to complete the online surveys and take part in the interview.

8. Privacy, Confidentiality and Disclosure of Information

We will keep all personal information confidential and securely stored. The electronic data we collect about you will be stored on a secure server hosted by the University of Southern Queensland. Hard copies of research data will be stored securely at the Prostate Cancer Foundation of Australia (PCFA) St Leonards office. Any information obtained in connection with this project that could identify you will remain confidential. Only authorised study staff will have access to these materials. It will only be disclosed with your permission, except as required by law. In any publication, information will be provided in such a way that you cannot be identified. Data will be stored for 25 years in accordance with the National Statement (2007) and institutional policy.

So that we can contact you to take part in an interview, we will ask you to provide an email address or a phone number. This will not be linked to any information we have about you in connection to the project.

For peer review only - http://bmjopen.bmj.com/site/about/guidelines.xhtml

Prostate Cancer Foundation of Australia

PROSTATE CANCER SURVIVORSHIP ESSENTIALS

University of Southern Queensland

Australian privacy law gives you the right to request access to your information that the researchers have collected and stored. The law also gives you the right to request corrections to any information about you that you disagree with. Please contact the study team (see page 3 of this document) if you would like to access your information.

9. Who is organising and funding the research?

This research study is being conducted by Professor Jeff Dunn AO, University of Southern Queensland, in partnership with the Prostate Cancer Foundation Australia (PCFA), Cancer Council Queensland (CCQ), Australian Prostate Centre (APC), Ipswich West Moreton Hospital Health Service (WMHHS) GenesisCare, Icon Group, Healthy Male, and the Union for International Cancer Control (UICC). The University of Southern Queensland will receive a payment from the NHMRC administered by the federal Department of Health for undertaking this research. No member of the *PCEssentials Hormone Therapy* Study team will receive any financial benefit from your involvement in this research study (other than their ordinary wages).

10. Who has reviewed the research study?

All research in Australia involving humans is reviewed by an independent group of people called a Human Research Ethics Committee (HREC). The ethical aspects of this research study have been approved by the Metro South HREC. This research study will be carried out according to the National Statement on Ethical Conduct in Human Research (2007). This statement has been developed to protect the interests of people who agree to participate in human research studies.

11. What if I have a question or need to make a complaint?

We have included several contacts for you below. The person you may need to contact will depend on the nature of your query.

For questions about the *PCEssentials Hormone Therapy* Study, you can contact the *PCEssentials Hormone Therapy* Study team:

- Coordinating Principal Investigator: Professor Jeff Dunn AO, 02 9428 7060
- Central Management Team: Dr Anna Green, 02 9428 7060, pcessentials@pcfa.org.au

To talk to someone at your participating site:

- Principal Investigator *[Study Site]*: *[Name & contact number]*

If you wish to discuss the study with someone who is not directly involved, particularly in relation to matters concerning complaints about the conduct of the study, or your rights as a participant, you can contact:

Lead HREC Office	Metro South Health and Hospital Services (MSHHS)
Contact Person	HREC Coordinator
Telephone	+61 7 3443 8049
Email	MSH-Ethics@health.qld.gov.au
HREC Reference Number	HREC/2021/QMS/79429

12. The Participant Consent Form

Sign the consent form only after you have made up your mind to take part in this study. You must be provided with a signed and dated copy of the participant information and consent form for your personal record.

For peer review only - http://bmjopen.bmj.com/site/about/guidelines.xhtml

Prostate Cancer Foundation of Australia

PROSTATE CANCER SURVIVORSHIP ESSENTIALS

University of Southern Queensland

Prostate Cancer Survivorship Essentials for Men with Prostate Cancer on Androgen Deprivation Therapy (PCEssentials Hormone Therapy Study)

Participant Consent Form – Clinical Stakeholders

**Title**	Prostate Cancer Survivorship Essentials for Men with Prostate Cancer on Androgen Deprivation Therapy (ADT): An Effectiveness-Implementation Hybrid (Type 1) Trial of a Tele-Based Nurse-Led Survivorship Care Intervention
**Short Title**	*PCEssentials Hormone Therapy Study*
**Coordinating Principal Investigator**	Professor Jeff Dunn AO

Declaration by Participant

- I have read, or have had read to me, and I understand the Participant Information Sheet and Consent Form.
- I understand the purposes, procedures and risks of the research described in the Participant Information Sheet.
- I have had an opportunity to ask questions and I am satisfied with the answers I have received.
- I freely agree to participate in this research study as described and understand that I am free to withdraw at any time during the study without affecting my future health care.
- I understand that I will be given a signed copy of this document to keep for my own records. We may ask you to participate in a future related study, or to obtain additional information or clarification related to your participation in this study. **Please indicate below** whether you are willing to be contacted about any future research studies.
- I agree to the research team using, reproducing, and disclosing audio-recordings as explained in the Participant Information Sheet/Consent Form for Patients.
- I agree to be audio-recorded and understand that, subject to any constraints requested below, recordings may be used in presentations and publications for educational and research purposes.

30-minute Interview

☐ Yes, I agree to be contacted and invited to participate in the interview

☐ No, I do not want to be contacted to be invited to participate in the interview

Future Studies

☐ Yes, I agree to be contacted about future research studies

☐ No, I do not want to be contacted about future research studies

Study Results

☐ Yes, I would like to receive a copy of the study results and acknowledge that these will be provided in aggregate as a summary (individual results will not be available)

☐ No, I do not want a copy of the study results

Participant

Signature ___________________________________ Date ___________________________

Name (please print) ___________________________________

Declaration by senior researcher

I have given a verbal explanation of the study, its procedures and risks and I believe that the participant has understood that explanation.

Signature ___________________________________ Date ___________________________

Name (please print) ___________________________________

For peer review only

SPIRIT

STANDARD PROTOCOL ITEMS: RECOMMENDATIONS FOR INTERVENTIONAL TRIALS

SPIRIT 2013 Checklist: Recommended items to address in a clinical trial protocol and related documents*

Section/item	Item No	Description	Y/N
**Administrative information**			
Title	1	Descriptive title identifying the study design, population, interventions, and, if applicable, trial acronym	Y p1
Trial registration	2a	Trial identifier and registry name. If not yet registered, name of intended registry	Y p1
	2b	All items from the World Health Organization Trial Registration Data Set	Y p1-14
Protocol version	3	Date and version identifier	Y p1
Funding	4	Sources and types of financial, material, and other support	Y p1
Roles and responsibilities	5a	Names, affiliations, and roles of protocol contributors	Y author page & p 14
	5b	Name and contact information for the trial sponsor	Y author page & p11
	5c	Role of study sponsor and funders, if any, in study design; collection, management, analysis, and interpretation of data; writing of the report; and the decision to submit the report for publication, including whether they will have ultimate authority over any of these activities	N/A
	5d	Composition, roles, and responsibilities of the coordinating centre, steering committee, endpoint adjudication committee, data management team, and other individuals or groups overseeing the trial, if applicable (see Item 21a for data monitoring committee)	Y p11
**Introduction**			
Background and rationale	6a	Description of research question and justification for undertaking the trial, including summary of relevant studies (published and unpublished) examining benefits and harms for each intervention	Y p3-4
	6b	Explanation for choice of comparators	Y p4

For peer review only

Objectives	7	Specific objectives or hypotheses	Y p4
Trial design	8	Description of trial design including type of trial (eg, parallel group, crossover, factorial, single group), allocation ratio, and framework (eg, superiority, equivalence, noninferiority, exploratory)	Y p4

Methods: Participants, interventions, and outcomes

Study setting	9	Description of study settings (eg, community clinic, academic hospital) and list of countries where data will be collected. Reference to where list of study sites can be obtained	Y p5
Eligibility criteria	10	Inclusion and exclusion criteria for participants. If applicable, eligibility criteria for study centres and individuals who will perform the interventions (eg, surgeons, psychotherapists)	Y p5
Interventions	11a	Interventions for each group with sufficient detail to allow replication, including how and when they will be administered	Y p5-8
	11b	Criteria for discontinuing or modifying allocated interventions for a given trial participant (eg, drug dose change in response to harms, participant request, or improving/worsening disease)	Y p12-13
	11c	Strategies to improve adherence to intervention protocols, and any procedures for monitoring adherence (eg, drug tablet return, laboratory tests)	Y p11-12
	11d	Relevant concomitant care and interventions that are permitted or prohibited during the trial	Y p12-13
Outcomes	12	Primary, secondary, and other outcomes, including the specific measurement variable (eg, systolic blood pressure), analysis metric (eg, change from baseline, final value, time to event), method of aggregation (eg, median, proportion), and time point for each outcome. Explanation of the clinical relevance of chosen efficacy and harm outcomes is strongly recommended	Y p8-9
Participant timeline	13	Time schedule of enrolment, interventions (including any run-ins and washouts), assessments, and visits for participants. A schematic diagram is highly recommended (see Figure)	Y p7
Sample size	14	Estimated number of participants needed to achieve study objectives and how it was determined, including clinical and statistical assumptions supporting any sample size calculations	Y p10
Recruitment	15	Strategies for achieving adequate participant enrolment to reach target sample size	Y p6

Methods: Assignment of interventions (for controlled trials)

Allocation:

Sequence generation	16a	Method of generating the allocation sequence (eg, computer-generated random numbers), and list of any factors for stratification. To reduce predictability of a random sequence, details of any planned restriction (eg, blocking) should be provided in a separate document that is unavailable to those who enrol participants or assign interventions	Y p6
Allocation concealment mechanism	16b	Mechanism of implementing the allocation sequence (eg, central telephone; sequentially numbered, opaque, sealed envelopes), describing any steps to conceal the sequence until interventions are assigned	Y p6
Implementation	16c	Who will generate the allocation sequence, who will enrol participants, and who will assign participants to interventions	Y p6
Blinding (masking)	17a	Who will be blinded after assignment to interventions (eg, trial participants, care providers, outcome assessors, data analysts), and how	Y p6
	17b	If blinded, circumstances under which unblinding is permissible, and procedure for revealing a participant's allocated intervention during the trial	N/A
**Methods: Data collection, management, and analysis**			
Data collection methods	18a	Plans for assessment and collection of outcome, baseline, and other trial data, including any related processes to promote data quality (eg, duplicate measurements, training of assessors) and a description of study instruments (eg, questionnaires, laboratory tests) along with their reliability and validity, if known. Reference to where data collection forms can be found, if not in the protocol	Y p8-10
	18b	Plans to promote participant retention and complete follow-up, including list of any outcome data to be collected for participants who discontinue or deviate from intervention protocols	Y p8
Data management	19	Plans for data entry, coding, security, and storage, including any related processes to promote data quality (eg, double data entry; range checks for data values). Reference to where details of data management procedures can be found, if not in the protocol	Y p12
Statistical methods	20a	Statistical methods for analysing primary and secondary outcomes. Reference to where other details of the statistical analysis plan can be found, if not in the protocol	Y p10-11
	20b	Methods for any additional analyses (eg, subgroup and adjusted analyses)	Y p10-11
	20c	Definition of analysis population relating to protocol non-adherence (eg, as randomised analysis), and any statistical methods to handle missing data (eg, multiple imputation)	Y p10-11

Methods: Monitoring

Data monitoring	21a	Composition of data monitoring committee (DMC); summary of its role and reporting structure; statement of whether it is independent from the sponsor and competing interests; and reference to where further details about its charter can be found, if not in the protocol. Alternatively, an explanation of why a DMC is not needed	Y p12
	21b	Description of any interim analyses and stopping guidelines, including who will have access to these interim results and make the final decision to terminate the trial	N/A
Harms	22	Plans for collecting, assessing, reporting, and managing solicited and spontaneously reported adverse events and other unintended effects of trial interventions or trial conduct	Y p12
Auditing	23	Frequency and procedures for auditing trial conduct, if any, and whether the process will be independent from investigators and the sponsor	Y p12

Ethics and dissemination

Research ethics approval	24	Plans for seeking research ethics committee/institutional review board (REC/IRB) approval	Y p11
Protocol amendments	25	Plans for communicating important protocol modifications (eg, changes to eligibility criteria, outcomes, analyses) to relevant parties (eg, investigators, REC/IRBs, trial participants, trial registries, journals, regulators)	Y p12
Consent or assent	26a	Who will obtain informed consent or assent from potential trial participants or authorised surrogates, and how (see Item 32)	Y p6
	26b	Additional consent provisions for collection and use of participant data and biological specimens in ancillary studies, if applicable	N/A
Confidentiality	27	How personal information about potential and enrolled participants will be collected, shared, and maintained in order to protect confidentiality before, during, and after the trial	Y p12
Declaration of interests	28	Financial and other competing interests for principal investigators for the overall trial and each study site	Y p14
Access to data	29	Statement of who will have access to the final trial dataset, and disclosure of contractual agreements that limit such access for investigators	Y p11-12
Ancillary and post-trial care	30	Provisions, if any, for ancillary and post-trial care, and for compensation to those who suffer harm from trial participation	Y p12-13

For peer review only

Dissemination policy	31a	Plans for investigators and sponsor to communicate trial results to participants, healthcare professionals, the public, and other relevant groups (eg, via publication, reporting in results databases, or other data sharing arrangements), including any publication restrictions	Y p13
	31b	Authorship eligibility guidelines and any intended use of professional writers	N/A
	31c	Plans, if any, for granting public access to the full protocol, participant-level dataset, and statistical code	Y p13
**Appendices**			
Informed consent materials	32	Model consent form and other related documentation given to participants and authorised surrogates	Y
Biological specimens	33	Plans for collection, laboratory evaluation, and storage of biological specimens for genetic or molecular analysis in the current trial and for future use in ancillary studies, if applicable	N/A

*It is strongly recommended that this checklist be read in conjunction with the SPIRIT 2013 Explanation & Elaboration for important clarification on the items. Amendments to the protocol should be tracked and dated. The SPIRIT checklist is copyrighted by the SPIRIT Group under the Creative Commons "Attribution-NonCommercial-NoDerivs 3.0 Unported" license.

For peer review only
