# Supplementary material for: Prostate Cancer Survivorship Essentials for men with prostate cancer on androgen deprivation therapy: protocol for a randomised controlled trial of a tele-based nurse-led survivorship care intervention (PCEssentials Hormone Therapy Study)
Source: BMJ Open. 2024 Mar 23;14(3):e084412. doi: 10.1136/bmjopen-2024-084412 (PMC10961537; doi:10.1136/bmjopen-2024-084412)
Supplement: Reviewer comments [file bmjopen-2024-084412.reviewer_comments.pdf]

PEER REVIEW HISTORY

BMJ Open publishes all reviews undertaken for accepted manuscripts. Reviewers are asked to complete a checklist review form (**http://bmjopen.bmj.com/site/about/resources/checklist.pdf**) and are provided with free text boxes to elaborate on their assessment. These free text comments are reproduced below.

ARTICLE DETAILS

**TITLE (PROVISIONAL)**	Prostate Cancer Survivorship Essentials for men with prostate cancer on androgen deprivation therapy: Protocol for a randomised controlled trial of a tele-based nurse-led survivorship care intervention (PCEssentials Hormone Therapy Study)
**AUTHORS**	Green, Anna; Newton, Robert; Smith, David; Tuffaha, Haitham; Galvão, Daniel; Heathcote, Peter; Patel, Manish; Christie, David; Egger, Sam; Sara, Sally; Heneka, Nicole; Chambers, Suzanne K; Dunn, Jeff

This article was not externally reviewed at BMJ Open. Protocols that have been independently assessed prior to submission to BMJ Open are usually fast-tracked to publication on the grounds that further substantial changes will not be possible. This independent assessment will usually be external, independent review for both a substantial grant award from a non-commercial or government funder and ethics approval.
